# Discovery of a functionally selective serotonin receptor (5-HT_1A_R) agonist for the treatment of pain

**DOI:** 10.1126/sciadv.adv9267

**Published:** 2025-06-18

**Authors:** Annika Ullrich, Johannes Schneider, João M. Braz, Eduard Neu, Nico Staffen, Markus Stanek, Jana Bláhová, Tamsanqa Hove, Tamara Albert, Anni Allikalt, Stefan Löber, Karnika Bhardwaj, Sian Rodriguez-Rosado, Elissa Fink, Tim Rasmussen, Harald Hübner, Asuka Inoue, Brian K. Shoichet, Allan I. Basbaum, Bettina Böttcher, Dorothee Weikert, Peter Gmeiner

**Affiliations:** ^1^ Department of Chemistry and Pharmacy, Friedrich-Alexander-Universität Erlangen-Nürnberg, Erlangen, Germany.; ^2^ Department of Anatomy, University of California, San Francisco, CA, USA.; ^3^ Rudolf-Virchow-Center, Julius-Maximilians-Universität, Würzburg, Germany.; ^4^ Department of Pharmaceutical Chemistry, University of California, San Francisco, CA, USA.; ^5^ Graduate Program in Biophysics, University of California, San Francisco, CA, USA.; ^6^ Graduate School of Pharmaceutical Sciences, Tohoku University, Sendai, Japan.; ^7^ Graduate School of Pharmaceutical Sciences, Kyoto University, Kyoto, Japan.; ^8^ FAU NeW, Erlangen, Germany.

## Abstract

The heterotrimeric G protein–coupled serotonin receptor 5-HT_1A_ receptor (5-HT_1A_R) mediates antinociception and may serve as a valuable target for the treatment of pain. Starting from a chemical library, we evolved ST171, a bitopic 5-HT_1A_R agonist that revealed highly potent and functionally selective G_i/o_ signaling without G_s_ activation and marginal β-arrestin recruitment. ST171 is effective in acute and chronic pain models. Cryo–electron microscopy structures of ST171 bound to 5-HT_1A_R in complex with the G_i_ protein compared to the canonical agonist befiradol bound to complexes of 5-HT_1A_R with G_i_ or G_s_ revealed that the ligands occupy different exo-sites. The individual binding poses are associated with ligand-specific receptor conformations that were further studied by molecular dynamics simulations, allowing us to better understand ligand bias, a phenomenon that may be crucial to the discovery of more effective and safe G protein–coupled receptor drugs.

## Teaser

Cryo-EM reveals the structure of serotonin receptor 5-HT_1A_R bound to ST171, a G_i/o_-biased agonist with antinociceptive activity.

## INTRODUCTION

The chronic treatment of pain by opioids is limited by common adverse side effects, including addiction, constipation, and respiratory depression (*1*). Efforts have been dedicated to the discovery of improved opioids, including opioid receptor partial agonists (*2*), and functionally selective ligands that are devoid of these side effects (*3*, *4*). Other approaches sought analgesics that target non–opioid-sensitive links in the pain processing circuitry.

**Fig. 1.**
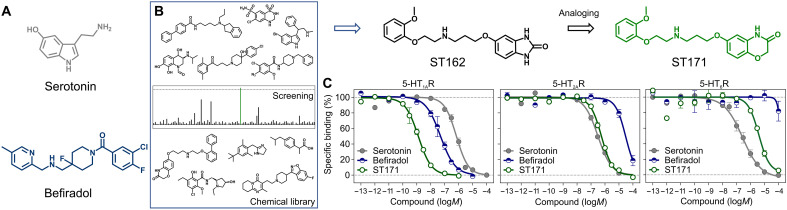
Discovery of ST171. (**A**) Chemical structures of serotonin and befiradol. (**B**) In-house library investigation of 5-HT_1A_R affinity using radioligand binding studies revealing ST162 that was further developed into its analog ST171. (**C**) Comparison of the binding affinities of serotonin, befiradol, and ST171 at the 5-HT_1A_R {displacement of [^3^H]WAY100635}, 5-HT_2A_R {displacement of [^3^H]ketanserin}, and 5-HT_6_R {displacement of [^3^H]LSD}. Data are shown with ±SEM of 4 to 11 independent experiments.

Of interest in this approach is the serotonin 5-HT_1A_ receptor (5-HT_1A_R), which is a Gα_i/o_-coupled receptor belonging to the family of serotonin receptors that comprises in total 12 heterotrimeric GTP-binding protein (G protein)–coupled receptors (GPCRs) and one ion channel (5-HT_3_R) (*5*). The 5-HT_1A_R is located pre- and postsynaptically in the brain (*5*) and is a clinically relevant target for the treatment of neuropsychiatric disorders (*6*), being the main target of the approved anxiolytic buspirone and the antidepressant vilazodone (*7*). Recently, structural studies applying cryo–electron microscopy (cryo-EM) revealed the molecular structure of the 5-HT_1A_R bound to buspirone, vilazodone, and other agonists (*7*–*9*). Notably, the 5-HT_1A_R is also highly expressed and can inhibit nociceptive processing circuits at different levels of the neuraxis (*10*, *11*), but its overall contribution to nociceptive processing and/or analgesia is not straightforward (*11*–*13*). Not only can serotonin exert both pro-inflammatory (*14*, *15*) and anti-inflammatory effects (*16*), but both pronociceptive (*17*, *18*) and antinociceptive actions have also been reported (*19*–*21*). Paradoxical actions at the G_i/o_-coupled 5-HT_1A_R subtype have also been reported (*22*–*25*), possibly due to differences in pain assays (*26*, *27*) or the mode of drug administration (*24*, *28*, *29*). The latter influence is relevant because the 5-HT_1A_R is widely expressed (*30*, *31*), including primary sensory neurons of the dorsal root ganglia and the spinal cord (*32*, *33*). In addition to having traditional analgesic properties, binding to the 5-HT_1A_R can provoke analgesia with inverse tolerance (*34*), a decrease in opioid-induced rewarding effects (*35*), and reduce the respiratory depression caused by fentanyl (*36*). Of particular interest is befiradol (Fig. 1A), a selective 5-HT_1A_R agonist and drug candidate that has antinociceptive properties comparable to clinically used opioids (*37*, *38*). Although befiradol can also reverse opioid-induced respiratory depression in rats, this reversal often occurs with concurrent hyperalgesia (*36*) and sedation (*39*). To leverage the potential of the 5-HT_1A_R as an antinociceptive target, here, we sought previously unkown chemotypes for the 5-HT_1A_R with functional selectivity for G_i/o_ signaling. It has been previously shown that new chemistry can lead to other biological properties, as different scaffolds may engage previously unoccupied subpockets, thereby stabilizing distinct conformational ensembles of the receptor in its active state. These distinct states may induce preferential activation of some signaling pathways over others, thus inducing functional selectivity (*3*, *40*). This way, ligands with antinociceptive activity and a preference for G_i/o/z_ signaling were successfully developed that target the μ-opioid (*3*) and α_2A_ adrenergic (*41*) receptors. Of note, functional selectivity at the 5-HT_1A_R has been associated with the preferential activation of either pre- or postsynaptic receptors and receptor subpopulations in distinct brain areas, allowing for a separation from desired and off-target effects (*42*). Yet, the contribution of the individual G protein subtypes and the recruitment of β-arrestins to the in vivo effects remain only partially understood (*43*).

Our objective is to find efficacious non-opioid analgesics without the hyperalgesic and sedative side effects of befiradol. Starting from a chemical library hit, we found that ST171, a ligand that shows high affinity for the 5-HT_1A_R, selectively activates 5-HT_1A_R–mediated G_i/o_ signaling and demonstrates antinociceptive activity in mouse models of inflammatory and neuropathic pain. Cryo-EM of active-state 5-HT_1A_R-G protein complexes and accompanying molecular dynamics (MD) simulations compared to the canonical 5-HT_1A_R partial agonist befiradol also provided an important structural understanding of the intriguing pharmacology of ST171.

## RESULTS

### Discovery of ST171

We have recently shown that the search for previously unidentified chemotypes is a very powerful strategy to find receptor ligands with preferential G_i/o_ signaling and promising in vivo antinociceptive activity (*3*, *41*). Hence, we could generate lead compounds for the development of effective and safe therapeutics. To identify previously unknown chemotypes for the 5-HT_1A_R, we inspected our in-house library, with ~10,000 compounds, for potential binders at the 5-HT_1A_R (Fig. 1B). The library included ~2000 Food and Drug Administration (FDA)–approved drugs and ~8000 bioactive compounds generated in our recent GPCR projects. In a screening of the approved drugs and a sublibrary that includes a set of structurally diverse aminergic receptor ligands, the 5-HT_1A_R affinity of ST162 (Fig. 1B; also see fig. S1 and Supplementary Text for the chemical synthesis), a compound that was part of an earlier dopamine receptor program, attracted our attention. Evaluation of 60 structural analogs of ST162 for their ability to displace [^3^H]WAY100,635 from the 5-HT_1A_R led us to ST171 (Fig. 1B), which displaced more than 80% of the radiolabeled ligand at a concentration of 10 nM. Compared to serotonin and befiradol, ST171 showed superior binding affinity for the 5-HT_1A_R in concentration-dependent competition assays [*K*_i_ (inhibition constant) = 0.41 nM] with 680-fold subtype selectivity over the 5-HT_2A_R and 8000-fold selectivity over the 5-HT_6_R (Fig. 1C and table S1). These latter receptors are representatives of G_q_-coupled and G_s_-coupled serotonin receptor subtypes (*5*), respectively, that are known to exhibit pronociceptive effects and are therefore regarded as off-targets for the development of antinociceptive agents activating the 5-HT_1A_R (*44*).

### ST171 is a functionally selective agonist of the 5-HT_1A_R

**Fig. 2.**
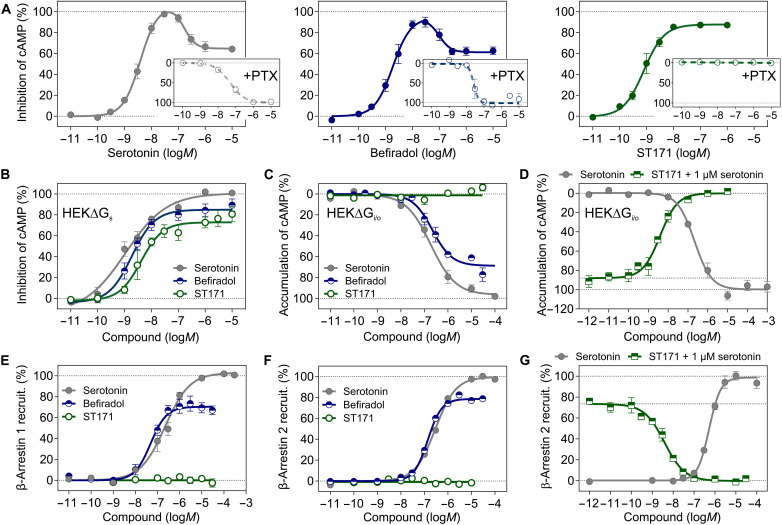
ST171 selectively activates G_i/o_ proteins via the 5-HT_1A_R. (**A**) An inhibition of 5-HT_1A_R–mediated cAMP accumulation is observed for serotonin, befiradol, and ST171. The stimulation of cAMP accumulation in the presence of PTX suggests an additional G_s_ response for befiradol and serotonin (serotonin: EC_50_ = 52 ± 6 nM; befiradol: EC_50_ = 25 ± 11 nM) but not for ST171. Data were obtained in three to five independent experiments with a CAMYEL assay in CHO-K1 cells. (**B**) Inhibition of cAMP accumulation by serotonin, befiradol, and ST171 in HEK293A cells deficient of G_s_ proteins and (**C**) accumulation of cAMP by serotonin and befiradol but not ST171 in HEK293A cells deficient of G_i/o_ proteins. Data were obtained with a GloSensor in *n* = 6 experiments, each performed in triplicates. (**D**) ST171 inhibits cAMP accumulation caused by 1 μM serotonin in HEK293AΔG_i/o_ cells and thus demonstrates functional antagonism for the activation of G_s_ proteins (IC_50_ = 3.9 ± 0.8 nM). Data were obtained with the GloSensor in *n* = 3 independent experiments, each performed as triplicates. (**E**) β-Arrestin 1 and (**F**) β-arrestin 2 recruitment is monitored by bystander BRET in HEK293T cells with elevated GRK2 levels [*n* = 4 to 6 (β-arrestin 1) or 11 to 14 (β-arrestin 2) independent experiments]. (**G**) ST171 is a functional antagonist (IC_50_ = 3.9 ± 0.5 nM) for 5-HT_1A_R–mediated β-arrestin 2 recruitment stimulated with serotonin (1 μM, ~EC_80_). Data were obtained with the PathHunter assay in the absence of GRK2 in three independent experiments, each performed in triplicates. All data were normalized to the maximum response of serotonin and are indicated as the means ± SEM of *n* independent experiments.

In vitro functional studies were initiated to explore the signaling profile of ST171 compared to serotonin and befiradol at the predominantly G_i/o_-coupled 5-HT_1A_R (*45*). Bioluminescence resonance energy transfer (BRET)–based sensing of adenosine 3′,5′-cyclic monophosphate (cAMP; CAMYEL biosensor) in CHO-K1 cells showed inhibition of cAMP formation in response to treatment with all three ligands. ST171 inhibited cAMP accumulation with high efficacy and subnanomolar potency [Fig. 2A; EC_50_ (mean effective concentration) = 0.88 ± 0.30 nM, *E*_max_ = 87 ± 1%]. We observed bell-shaped concentration-response curves for befiradol and serotonin (Fig. 2A), indicating reduced inhibition of adenylyl cyclase at higher ligand concentrations. G_s_-coupling in competition with G_i/o_-coupling for befiradol and serotonin was also suggested by further experiments including cAMP accumulation assays in the presence of pertussis toxin (PTX) (Fig. 2A) (*46*), the variation of the cAMP biosensor (GloSensor) and the cell line (fig. S2A and table S2), the effect of the knockout (KO) of Gα_i/o_ or Gα_s_ subunits (Fig. 2, B and C, and table S3), and inositol phosphate (IP) accumulation experiments with hybrid G_qi/s_ proteins (fig. S2B). Substantial activation of G_s_ in parallel to G_i/o_ was also observed for the well-known 5-HT_1A_R agonist 5-carboxytryptamine, while (*S*)-(−)-lisuride, 8-hydroxy-2-(di-*N*-propylamino)tetralin (8-OH-DPAT), and lysergic acid diethylamide-d3 (LSD-d3) acted as strong partial agonists for G_i/o_-coupling and moderate partial agonists for G_s_-coupling (fig. S3, A and B, and table S3). In contrast, the concentration-response curve for cAMP inhibition by ST171 was sigmoidal and PTX addition completely blocked the effect (*E*_max_ < 5%), indicating selective signaling through G_i/o_ proteins (Fig. 2A). ST171 did not elicit an effect in human embryonic kidney (HEK) 293A cells lacking Gα_i/o_ (*47*), while the deletion of G_s_ (*48*) had no effect on ligand efficacy [Fig. 2, B and C, and table S3; *E*_max_(ΔG_i/o_) < 5%, EC_50_(ΔG_s_) = 6.4 nM, and *E*_max_(ΔG_s_) = 73%]. Furthermore, intracellular IP accumulation in the presence of the hybrid Gα_qs_ protein was not observed for ST171, confirming its functional selectivity for G_i/o_ over G_s_ [fig. S2B; *E*_max_(G_qs_) < 5%]. In HEKΔG_i/o_ cells, ST171 potently inhibited the accumulation of cAMP elicited by 1 μM serotonin, confirming the de facto antagonism of ST171 for G_s_-mediated signaling by the 5-HT_1A_R [Fig. 2D; IC_50_ (mean inhibitory concentration) = 3.9 ± 0.8 nM]. Selective activation of the G_i/o_ pathway was also observed for the clinically used atypical neuroleptics ziprasidone and aripiprazole, albeit at more than 70-fold lower potency compared to ST171 (fig. S3, A and B, and table S3).

Overexpression of the different Gα_i/o_ subtypes Gα_i1_, Gα_i2_, Gα_i3_, Gα_z_, or Gα_oA_ rescued the inhibition of cAMP formation by ST171 in the HEK293AΔG_i/o_ cells and revealed a higher efficacy for the activation of Gα_oA_ (*E*_max_ = 100% relative to serotonin) and Gα_z_ (*E*_max_ = 93%) compared to the other Gα_i_ subtypes (Gα_i1–3_; *E*_max_ = 57 to 80%; fig. S2C and table S4). Concentration-response curves for ST171 were again sigmoid, while we observed partly bell-shaped profiles for serotonin and befiradol, indicating competition of G_s_ activation with G_i/o_ activation at higher concentrations of these two ligands (fig. S2C).

In β-arrestin KO HEK293A cells (*49*) (*E*_max_ = 60%) and the IP accumulation assay in the presence of Gα_qi_ (*E*_max_ = 43%), ST171 showed partial agonist activity (fig. S2, A and B, and table S3). We confirmed the partial agonist effect of ST171 in a BRET biosensor–based Gα_i1_ activation assay at different receptor expression levels. While serotonin and befiradol showed almost identical efficacies and only a minor reduction in potency at low receptor levels, the intrinsic activity of ST171 diminished gradually, from 77% to about 32% by decreasing 5-HT_1A_R expression toward the detection limit (fig. S2, D and E, and table S4). These data suggest that in vivo, ST171 likely acts as a partial agonist because endogenous expression levels are expected to be generally lower than those obtained in recombinant expression systems.

ST171 did not stimulate the recruitment of β-arrestin 1 or 2 to the 5-HT_1A_R, which was determined by enhanced bystander BRET using *Renilla* luciferase–tagged β-arrestin 2 and membrane-anchored *Renilla* green fluorescent protein (GFP-CAAX) (*50*) with cotransfected G protein–coupled receptor kinase 2 (GRK2) (Fig. 2, E and F; *E*_max_ < 5%) and only induced minor β-arrestin 2 recruitment in the presence of elevated GRK2 levels in an enzyme complementation–based assay [fig. S2F; *E*_max_(native GRK2) < 5% and *E*_max_(elevated GRK2) = 15%]. In contrast, stimulation of the 5-HT_1A_R with serotonin and befiradol robustly promoted the recruitment of β-arrestin 1 and 2 under the same conditions (Fig. 2, E and F, and table S3) and even in the absence of GRK2 (fig. S2F). ST171 potently inhibited serotonin-stimulated β-arrestin 2 recruitment, demonstrating a de facto antagonism of ST171 for this 5-HT_1A_R signaling pathway (IC_50_ = 3.9 ± 0.5 nM; Fig. 2G). Similar to the G protein activation properties, 5-carboxytryptamine was found to be a full agonist and showed the highest potency and efficacy among the tested ligands, while LSD-d3, (*S*)-(−)-lisuride, and 8-OH-DPAT were confirmed to be partial agonists. Ziprasidone and buspirone did not lead to substantial recruitment of β-arrestin 2, and aripiprazole only induced slight recruitment at concentrations >10 μM (fig. S3C and table S3).

Together, we identified ST171 as a highly potent 5-HT_1A_R agonist with functional selectivity for activation of G_i/o_ proteins over G_s_ and recruitment of β-arrestin. To determine ST171’s selectivity profile, we measured its affinity in ligand binding assays with a set of 23 other class A GPCRs (table S5). We found low to moderate affinity, e.g., for the opioid receptor family (*K*_i_ > 5 μM) and α_2A_ adrenergic receptors (*K*_i_ = 260 nM), which are known targets for pain alleviation (*3*, *41*). In contrast, ST171 has a high affinity for dopamine D_4_ and α_1A_ adrenergic receptors (*K*_i_ = 0.17 and 0.49 nM, respectively) and considerable affinity for the D_2S_ and α_1B_ receptor subtypes (*K*_i_ = 8.5 and 6.7 nM, respectively). As α_1_R agonism may have an influence on blood pressure and heart rate (*51*), we investigated ST171 in an IP accumulation assay. The results indicated that ST171 is devoid of intrinsic activity at α_1A_R and α_1B_R (fig. S4, A and B), as well as the related α_2A_R, α_2B_R, and α_2C_R subtypes (fig. S4, C to E). The putative α_1_ receptor antagonism of ST171 was further confirmed in inhibition studies with α_1A_R and norepinephrine (IC_50_ = 12 nM; fig. S4A). In contrast, we measured partial agonist activity for D_4_R in a BRET Gα_i1_ dissociation assay (EC_50_ = 1.6 nM and *E*_max_ = 42%) and for D_2S_R in an IP accumulation assay with a hybrid G_qi_ protein (EC_50_ = 2.5 nM and *E*_max_ = 75%). The experiments with the dopamine D_2S_ and D_4_ receptors also indicated a bias for G_i/o_ proteins because ST171 showed only weak efficacy in β-arrestin 2 recruitment assays with these two receptors in the absence of GRK2 (fig. S4, F to I). Similar to the observations at the 5-HT_1A_R, overexpression of GRK2 enhanced D_4_R-mediated β-arrestin 2 recruitment (fig. S4G). Functional studies with further 5-HT_1_ (1B, 1D, 1E, and 1F) and 5-HT_2_ (2A and 2C) receptor subtypes (fig. S4, J to O) revealed that ST171 potently activates the closely related Gα_i/o_-coupled 5-HT_1B_R (EC_50_ = 3.7 nM and *E*_max_ = 94%) and is a potent partial agonist for the 5-HT_1D_R (EC_50_ = 5.7 nM and *E*_max_ = 54%) but has no agonist activity at the 5-HT_1E_R and 5-HT_1F_R subtypes (*E*_max_ < 10%). The 5-HT1_B/D/F_ subtypes are the primary targets of triptans, which are well-established antimigraine drugs, because agonism at these 5-HT_1_R subtypes leads to vasoconstriction of painfully dilated cerebral blood vessels (*5*). Moreover, ST171 has substantial selectivity over the Gα_q_-coupled receptors of the 5-HT_2_ family, as illustrated by the low-potency and low-efficacy partial agonist activity that we observed with the 5-HT_2A_R (EC_50_ = 1500 nM and *E*_max_ = 12%) and 5-HT_2C_R (EC_50_ = 730 nM and *E*_max_ 26%). 5-HT_2_ receptors are known to mediate the hallucinogenic effects of diverse psychedelics (*7*) and are furthermore associated with fibrosis, a severe adverse effect, e.g., in the heart and lungs (*5*).

In vitro stability assays with ST171 and rat liver microsomes revealed favorable metabolic stability with more than 70% of intact substrate remaining after 1 hour of incubation (fig. S5A). Moreover, pharmacokinetic studies in mice demonstrated that ST171 is able to penetrate rapidly into the brain (fig. S5, B and C).

### ST171 reduces hypersensitivity in mouse models of chronic neuropathic and inflammatory pain

**Fig. 3.**
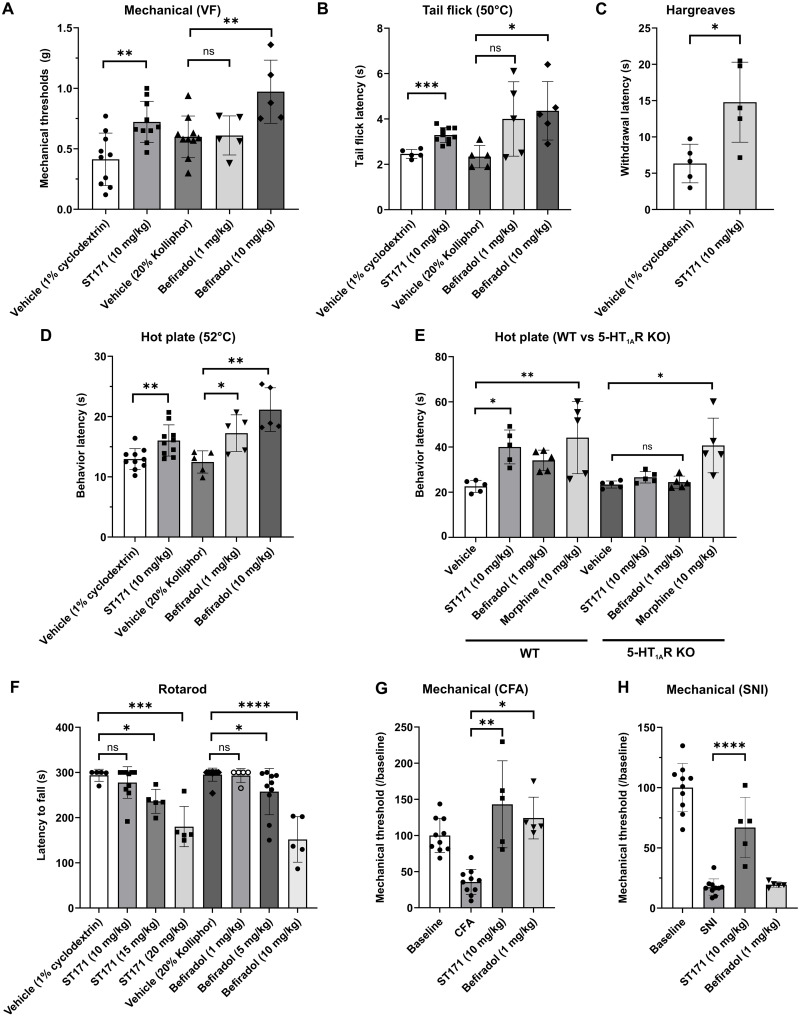
ST171 is analgesic in acute pain tests and antihyperalgesic in mouse models of chronic (neuropathic and inflammatory) pain. (**A**) In vivo efficacy of ST171 and befiradol in the von Frey assay. (**B** to **D**) Antinociceptive effects of ST171 and befiradol in different thermal assays: 50°C tail flick (B), Hargreaves (C), and 52°C hot plate (D). (**E**) ST171 and befiradol have no significant antinociceptive effect in 5-HT_1A_R KO mice in the hot plate test. (**F**) Effect of increasing doses of ST171 and befiradol on motor performances in the rotarod assay. (**G**) Antihyperalgesic effects of ST171 and befiradol in the CFA-induced inflammatory pain model. (**H**) Antiallodynic effects of ST171 and befiradol in the SNI model of neuropathic pain. All data represent the means ± SD of 5 to 10 animals. The effects of ST171 and befiradol were compared to their respective vehicle in (A) to (D) and compared to SNI in (H) using Student’s *t* test with *P < 0.05, **P < 0.01, ***P < 0.005, and ****P < 0.001, ns not significant. Differences between groups and doses were measured using the one-way ANOVA in (F) (Tukey’s post hoc test) and two-way ANOVA in (E) (Sidak’s post hoc test) and (G) (Tukey’s post hoc tests).

We tested the antinociceptive properties of ST171 under both acute and chronic (neuropathic and inflammatory) pain conditions. We used a 10 mg/kg intraperitoneal dose of ST171, which showed favorable pharmacokinetics (fig. S5, B and C), and compared the results to the action of befiradol, also at 10 mg/kg (*25*). A systemic injection of both ST171 and befiradol was antinociceptive in acute mechanical and thermal pain assays, significantly increasing mechanical thresholds (Fig. 3A) as well as the response latency to thermal stimuli in three different tests of “heat pain” (Fig. 3, B to D). In the Hargreaves test, we also observed a dose dependency for ST171, as lower doses (1 and 5 mg/kg) were not as antinociceptive as the 10 mg/kg dose. Despite a significant result by the analysis of variance (ANOVA), the post hoc analysis did not reveal statistical differences for the lower doses (fig. S6A). In line with a 5-HT_1A_R–mediated antinociceptive effect, in the hot plate test, neither ST171 (10 mg/kg) nor befiradol (1.0 mg/kg) treatment was antinociceptive in 5-HT_1A_R KO mice. In contrast, morphine (10 mg/kg) significantly increased the response latency in both wild-type (WT) and KO animals (Fig. 3E). When tested for sedative effects using an accelerating rotarod, mice injected with ST171 (10 mg/kg) did not differ from control mice injected with vehicle (Fig. 3F). Only at a 15 mg/kg dose of ST171 did we observe a presumptive sedative effect on the rotarod. In contrast, all doses >1 mg/kg of befiradol significantly reduced the ability of the mouse to continue on the rotarod.

We next assessed the efficacy of ST171 and befiradol in two models of chronic pain, the spared nerve injury (SNI) model (*52*) of neuropathic pain and the complete Freund’s adjuvant (CFA)–induced inflammatory pain model (*41*). The 10 mg/kg intraperitoneal dose of ST171 significantly increased the mechanical thresholds of the injured hind paw in both the inflammatory (Fig. 3G) and neuropathic (Fig. 3H) models. Mechanical thresholds returned to baseline levels in the CFA but not the SNI model. In contrast, the nonsedating dose of befiradol (1 mg/kg) only reduced the mechanical sensitivity in the CFA-induced pain model. Together, our results demonstrate that a systemic administration of ST171 has broad therapeutic potential across multiple modalities of acute and chronic pain and is clearly superior to befiradol.

### The 5-HT_1A_R agonist ST171 is inherently rewarding

Although pain relief is rewarding per se, it is essential to determine whether a compound is intrinsically rewarding, even in the absence of pain relief, as this property could limit its usage for pain management. As there are reports that 5-HT_1A_R agonists, including 8-OH-DPAT (*53*), buspirone, and gepirone (*54*), have dose-dependent reinforcing effects, we investigated the rewarding properties of ST171. Here, we used the conditioned place preference (CPP) assay, in which mice learn to associate one chamber of the CPP apparatus with a particular compound. If mice show a preference for the compound-paired chamber, we conclude that the compound is inherently rewarding. Here, we found that mice injected with ST171 (10 mg/kg) spent more time in the ST171-paired chamber than in the vehicle-injected chamber (fig. S6B), indicating that ST171 induces CPP, which is consistent with previous reports describing the rewarding effects of 5-HT_1A_R agonists.

### The 5-HT_1A_R agonist ST171 has anxiolytic properties at low doses

Whether 5-HT_1A_R agonists can reduce anxiety is still a matter of debate. For example, both 5-HT_1A_R agonists and antagonists have been reported to exert anxiolytic effects (*55*, *56*), no effect (*57*), or even anxiogenic effects (*58*). These differences are likely due to variations in experimental procedures, including the route of administration, the assays performed, whether the compounds tested are full or partial agonists, and/or the doses used. As the 5-HT_1A_R agonist befiradol has been reported to increase exploratory behavior in an open field and the time spent in open arms in the plus maze assay (*59*), suggesting an anxiolytic action, we also investigated whether ST171 has any anxiolytic potential. Here, mice received a single dose of ST171 or vehicle 30 min before they were placed in the plus maze apparatus, and we recorded the time spent in each compartment over 5 min. Compared to vehicle control, ST171 significantly increased the time mice spent exploring the center and the open arms (fig. S6C). Conversely, ST171-injected mice spent less time in the closed arms. The effect was unusually dose dependent. At the analgesic dose of ST171, the anxiolytic effects of the drug disappeared. We conclude that ST171 dose-dependently produces an anxiolytic-like effect in the plus maze assay but that separation of the analgesic and anxiolytic effects is possible.

### Structural studies reveal distinct binding modes of ST171 and befiradol in complexes with 5-HT_1A_R-G_i1_ or 5-HT_1A_R-G_s_

**Fig. 4.**
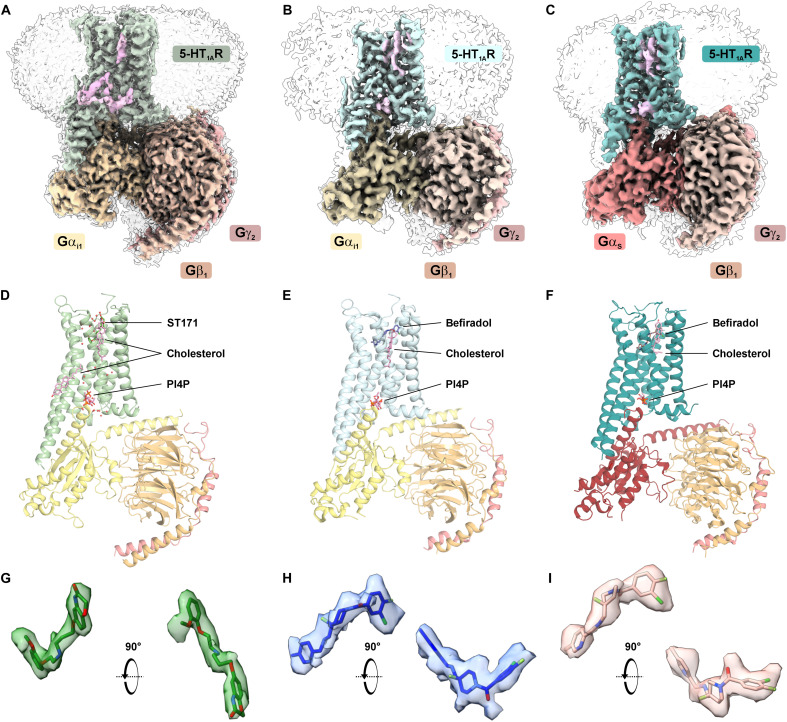
Cryo-EM structures reveal the overall architecture of ST171 bound to 5-HT_1A_R-G_i1_ and befiradol bound to 5-HT_1A_R-G_i1_ and 5-HT_1A_R-G_s_ protein complexes. Cryo-EM maps (**A** to **C**) and models (**D** to **F**) of [(A) and (D)] the ST171-bound 5-HT_1A_R-G_i1_ protein complex with two cholesterol molecules and PI4P (light pink), [(B) and (E)] the befiradol-bound 5-HT_1A_R-G_i1_ protein complex, and [(C) and (F)] the befiradol-bound 5-HT_1A_R-G_s_ protein complex, each with one cholesterol molecule and PI4P (light pink). (**G** to **I**) Zoom-in of the densities corresponding to the ligands viewed from the side [left, as in (D) to (F)] and top of the receptor (right, 90° rotation) view for (G) ST171, (H) befiradol in the 5-HT_1A_R complex with G_i_, and (I) befiradol in the complex of 5-HT_1A_R with G_s_.

We determined cryo-EM structures of 5-HT_1A_R-G_i1_ protein complexes bound to ST171 and befiradol at nominal resolutions of 2.4 and 2.9 Å, respectively, and performed unbiased MD simulations (10 μs for each ligand-bound protein complex), mutagenesis, and structure-activity-relationship (SAR) studies. In addition, we solved a structure of befiradol engaging the 5-HT_1A_R in complex with the G_s_ protein at a nominal resolution of 3.2 Å. As we did not observe evidence supporting ST171-induced coupling of G_s_, we did not attempt to obtain a structure of the 5-HT_1A_R-G_s_ complex in the presence of ST171. The cryo-EM maps enabled the modeling of the active-state receptor-G protein complexes and the placement of the ligands in the orthosteric binding pockets (Fig. 4, A to I, and fig. S7).

Our agonist-bound 5-HT_1A_R-G_i1_ protein complexes show the typical hallmarks of active-state complexes (*60*), including a downward orientation of the toggle switch residue W358^6.48^ (numbers in superscript correspond to Ballesteros-Weinstein numbering), the conformation of I124^3.40^ and an outward swing of F354^6.44^ in the PIF motif and NPxxY motif, and a break of the ionic lock formed by R134^3.50^ and D133^3.49^ (DRY motif) by a rotation of R134^3.50^. R134^3.50^ is further stabilized by a phosphatidylinositol-4-phosphate (PI4P) molecule located at the interface with the G_i1_ protein. PI4P has consistently been observed in 5-HT_1A_R-G_i_ structures and has previously been shown to act as a positive modulator for 5-HT_1A_R signaling (*7*, *8*). All these conformational properties contribute to an active state–like inward shift of transmembrane helix 7 (TM7) by ~3 Å and an outward movement of TM6 by ~9 Å to facilitate G_i_ protein binding (fig. S8A) (*61*). The complex of befiradol with 5-HT_1A_R and the G_s_ protein revealed minor changes at the macromolecular and microswitch level, leading to similar orientations of the toggle switch residue W358^6.48^, the PIF and NPxxY motifs, and the break of the ionic lock. Again, we observed a density that could correspond to a PI4P molecule, which thus seems to generally interact with the 5-HT_1A_R. The conformational changes cumulate in an outward movement of TM6 that is highly similar to our G_i1_-bound complexes (fig. S8B). The densities for the 5-HT_1A_R-G_s_ complex suggest an extension of TM5 by one helical turn and a slight kink of TM6, even though cryo-EM data indicate variability at the intracellular ends of TM5 and TM6. However, the overall complex architecture is most similar to our 5-HT_1A_R-G_i_ protein–coupled structures (fig. S8B). Comparable observations have been described for the β_2_ adrenergic receptor when the structure of the receptor bound to the secondary coupling partner (G_i_) was found to be highly similar to the structure of the receptor in complex with the preferred G protein (G_s_) (fig. S8C) (*62*). In conclusion, the overall structure of the receptor-G protein complex seems to be determined by the nature of the receptor rather than by the subtype of the G protein.

**Fig. 5.**
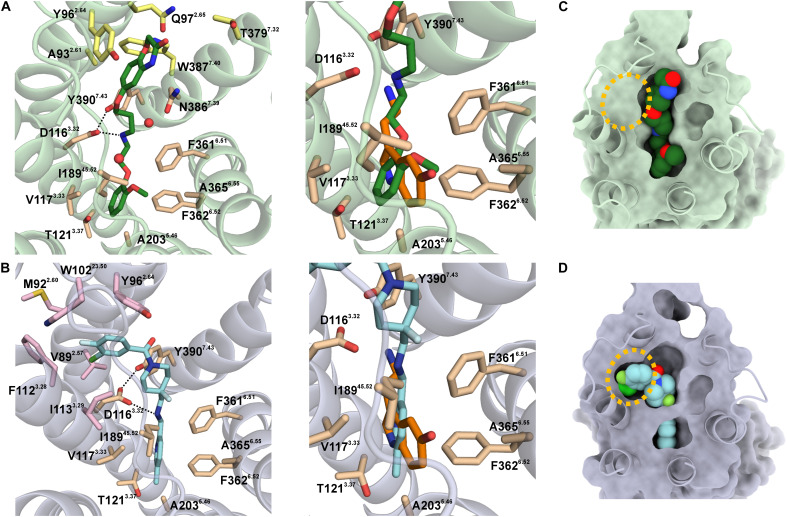
ST171 and befiradol engage the 5-HT_1A_R-G_i1_ complex via distinct extended binding pockets located close to TM2 and TM7. Top view of (**A**) ST171 and (**B**) befiradol binding poses and close-up comparison to the serotonin binding mode (PDB: 7E2Y; orange) in the orthosteric pocket. (**C** and **D**) Surface representation of ST171-bound (light green) and befiradol-bound (gray) 5-HT_1A_R illustrates the different extended binding pockets addressed by the ligands.

We observed several differences at the extracellular portions and in the binding poses of befiradol and ST171 in the 5-HT_1A_R-G_i_ complexes, which may explain their distinct signaling signatures. Both agonists show a bitopic binding mode occupying the conserved orthosteric pocket but addressing distinct allosteric sites of the 5-HT_1A_R (Fig. 5). The methoxyphenyl-substituted aminoethoxy group of ST171 and the methyl pyridinyl–substituted aminomethyl moiety of befiradol occupy a similar space in the orthosteric binding pocket, which endogenously engages serotonin and mainly interacts with V117^3.33^, I167^4.56^, I189^45.52^, A203^5.46^, F361^6.51^, F362^6.52^, and A365^6.55^ through hydrophobic interactions. Neither befiradol nor ST171 engages T121^3.37^, a residue that has been observed to interact with the indole NH of serotonin (Fig. 5, A and B) (*8*). Both ligands ST171 and befiradol share one hydrophilic key interaction with D116^3.32^, to which their secondary amines form a charge-reinforced hydrogen bond. This interaction is commonly seen in aminergic GPCRs, including the serotonin-bound 5-HT_1A_R (*8*). The distances between the secondary amines and D116^3.32^ suggest notable discrepancies, measuring 3.4 Å for ST171 and 4.0 Å for befiradol (distance between the secondary amine of the ligand and Cγ of D116^3.32^; fig. S9A). This disparity is particularly intriguing, given that D116^3.32^ generally serves as a critical anchor for basic moieties of aminergic ligands (*63*). MD simulations of both G_i_ complexes substantiated the persistence of this distinction (average distances: 3.1 and 3.8 Å for ST171 and befiradol, respectively) and showed higher fluctuation for the befiradol head group (fig. S9, B to E). Insertion of a CH_2_ unit between the head group and the secondary amine of ST171 as part of a SAR study led to an attenuation of binding and receptor activation (compound TA12; fig. S1C), further confirming the importance of the charge-reinforced hydrogen bond. In addition, we detected a water molecule in the vicinity of the ether group of the methoxyphenol ring of ST171 in the cryo-EM structure, which is likely involved in a water-mediated hydrogen bond with D116^3.32^. In accordance, a replacement of ST171’s ether function with a thioether unit, a weaker hydrogen bond acceptor (*64*), showed a considerable reduction of potency and efficacy (compound TA48; fig. S1C), indicating a contribution of the methoxy group to the binding energy. The cryo-EM structure also revealed the presence of a water-mediated hydrogen bond between the secondary amine of ST171 and the backbone carbonyl of N386^7.39^.

**Fig. 6.**
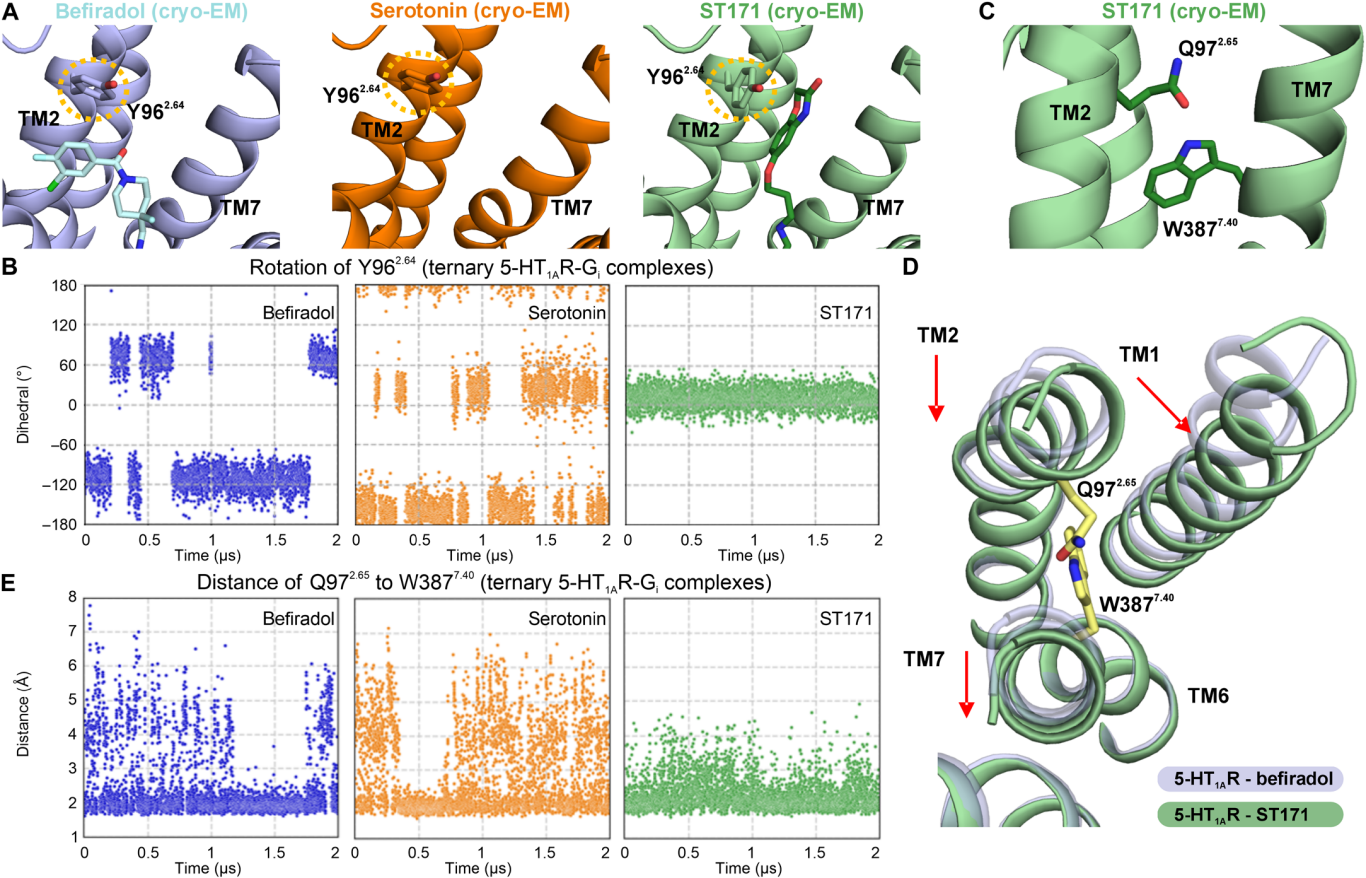
The benzoxazinone of ST171 contributes to the ligand-specific stabilization of the 5-HT_1A_R. (**A**) Conformation of Y96^2.64^ in the experimental cryo-EM structures of the befiradol-bound (PDB: 8PKM), serotonin-bound (PDB: 7E2Y), and ST171-bound (PDB: 8PJK) 5-HT_1A_R-G_i_ complexes. (**B**) Progression of the torsion angle -C_α_-C_β_-C_γ_-C_δ_- of the Tyr96^2.64^ side chain over the course of 2 μs for one of five independent simulations of the respective ligand-bound ternary 5-HT_1A_R complexes (simulation restraint on the G_i_ protein interface). (**C**) The cryo-EM structure of 5-HT_1A_R in complex with ST171 suggests the presence of a hydrogen bond between the amide group of Q97^2.65^ and the NH group of W387^7.40^. (**D**) Inward shift of TM2 and outward shift of TM1 and TM7 of the ST171-bound 5-HT_1A_R-G_i_ structure (light green) in comparison to the befiradol-bound structure (light gray) (extracellular view) with highlighted residues Q97^2.65^ and W387^7.40^. (**E**) Progression of the distance between Q97^2.65^ and W387^7.40^ is shown over the course of 2 μs for one of five independent simulation runs of the respective ligand-bound 5-HT_1A_R as a ternary complex (restraint on the G_i_ protein interface).

Although both ligands overlap in the orthosteric binding pocket, befiradol and ST171 address different extended binding sites of the 5-HT_1A_R-G_i_ complexes (Fig. 5, C and D). The benzoxazinone moiety of ST171 reaches a cleft formed by TM2 and TM7 where it interacts with A93^2.61^ and Y96^2.64^ through hydrophobic interactions, with Q97^2.65^ and W387^7.40^ through polar interactions, and with N386^7.39^ through water-mediated interactions (Fig. 5A). Notably, Y96^2.64^ adopts a conformation in which its phenyl moiety aligns nearly parallel to TM2, facilitating a π-π interaction between ST171’s benzoxazinone ring and Y96^2.64^. This conformation deviates from the orientation of Y96^2.64^ observed in the serotonin and befiradol G_i_ complexes (Fig. 6A). MD simulations with the ST171-bound complex corroborated a conformational restriction of the Y96^2.64^ side chain. Simulations for befiradol and serotonin showed freely rotatable side-chain orientations, with a main conformation of Y96^2.64^ that was nearly identical to the one observed with ST171 for serotonin, while befiradol exhibited a preference for an almost perpendicular orientation of Y96^2.64^ (Fig. 6B and fig. S10, A to D). Mediated by its engagement in π-π stacking with Y96^2.64^, ST171’s benzoxazinone can stabilize an interhelical hydrogen bond that we observed between the amide group of Q97^2.65^ and the NH group of W387^7.40^ (Fig. 6C). This interhelical hydrogen bond is an indicator of a closed arrangement of the extracellular side of the receptor with a reduced TM2 and TM7 distance, a higher distance between TM1 and TM2, and a disposition of TM7 away from TM6 (Fig. 6D), as revealed by the comparison of the ST171- and befiradol-bound 5-HT_1A_R complexes. In agreement with its extended-pocket binding pose, ST171’s affinity decreased more than 150-fold when Y96^2.64^ and Q97^2.65^ were replaced by alanine (*K*_i_ = 63 nM for Y96^2.64^A and Q97^2.65^A and *K*_i_ = 0.41 nM for WT). In contrast, the affinities of serotonin and befiradol were only affected to a minor extent (2.4- to 3.6-fold for serotonin and 4.2- to 13.3-fold for befiradol; table S1). Alanine mutation of residue W387^7.40^ markedly affected 5-HT_1A_R expression, precluding the reliable determination of binding affinities but highlighting the importance of this residue for proper 5-HT_1A_R trafficking and function. Additional SAR studies further confirmed the crucial role of ST171’s benzoxazinone moiety. Modifying the position of the benzoxazinone by shortening or extending the linker unit of ST171 resulted in a substantial decrease in both potency and efficacy (compounds TA13 and TA14; fig. S1C). Although the presence of a cholesterol molecule at the interface of TM2 and TM7 hampered the exact modeling of the side chain of Q97^2.65^, the distance of the respective side chains of Q97^2.65^ and W387^7.40^ observed in the befiradol-bound G_i_ complex confers an open state. To confirm the influence of ST171 and befiradol on this intrahelical interaction, MD simulations were performed (Fig. 6E and fig. S10, E to H). The data for the befiradol-bound receptor indicate an equilibrium between a closed state and an open state of the two residues in the ternary 5-HT_1A_R-G_i_ complex model (restraint G_i_ protein interface), while the open state without a hydrogen bond is favored in the binary complex model (unrestraint G protein interface). In contrast, MD simulations with the ST171-bound receptor generally show the high stability of the hydrogen bond between Q97^2.65^ and W387^7.40^ and, thus, the closed state. We propose that this interaction may contribute to the G_i/o_-biased signaling of ST171. Despite their strong impact on ST171’s 5-HT_1A_R affinity, the single-point mutations Y96^2.64^A and Q97^2.65^A only caused a minor reduction of ST171’s potency for Gα_i/o_ signaling (two- to threefold) and failed to enable the ST171-mediated stabilization and signaling of the Gα_s_-coupled 5-HT_1A_R in the manner of befiradol or serotonin (fig. S10, I and J, and table S3).

In contrast to ST171, the allosteric appendage of befiradol, a 3-chloro-4-fluorobenzene moiety, occupies a hydrophobic binding site between TM2, TM3, and extracellular loop 1 (ECL1) built from V89^2.57^, M92^2.60^, Y96^2.64^, W102^23.50^, F112^3.28^, and I113^3.29^. This cavity is not open for ligand binding in the ST171-bound and other 5-HT_1A_R structures (*7*–*9*) and becomes accessible for befiradol upon movement of the side chains of F112^3.28^ and M92^2.60^ outward of the TM bundle (Fig. 5B). The importance of F112^3.28^ for the binding mode and 5-HT_1A_R selectivity of befiradol was further evaluated by MD simulations and residue substitution. In MD simulations starting from the befiradol-bound 5-HT_1A_R-G_i_ complex (fig. S11A), this hydrophobic cavity closed within 150 ns after the removal of the ligand by a conformational change of F112^3.28^ and M92^2.60^. A spontaneous formation of the pocket could not be observed throughout our simulations. These data suggest that the hydrophobic pocket is formed by an induced fit mechanism in which the 3-chloro-4-fluorobenzene moiety of befiradol forces entry into the newly formed hydrophobic pocket rather than by conformational selection. This hypothesis is confirmed by metadynamics simulations indicating that an energy barrier of ~7.5 kcal/mol has to be overcome to facilitate the opening of the hydrophobic pocket (fig. S11B). Instead of F112^3.28^, this cavity is closed by a larger tryptophan residue in more than 70% of the aminergic GPCRs. Mutation of F112^3.28^ to tryptophan resulted in a 16-fold decrease in the binding affinity of befiradol [*K*_i_ (5-HT_1A_R-F112^3.28^W) = 240 nM], whereas it slightly increased the binding affinities of serotonin [*K*_i_ (5-HT_1A_R-F112^3.28^W) = 140 nM] and ST171 [*K*_i_ (5-HT_1A_R-F112^3.28^W) = 0.16 nM; table S1]. Additional mutation of M92^2.60^ to phenylalanine, which is present in more than 30% of the aminergic GPCRs, further closed the pocket and intensified the decrease in the affinity of befiradol (*K*_i_ = 330 nM) and the increase observed for ST171 (*K*_i_ = 0.044 nM) and serotonin (*K*_i_ = 53 nM; table S1), highlighting the importance of this subpocket for the 5-HT_1A_R selectivity of befiradol.

**Fig. 7.**
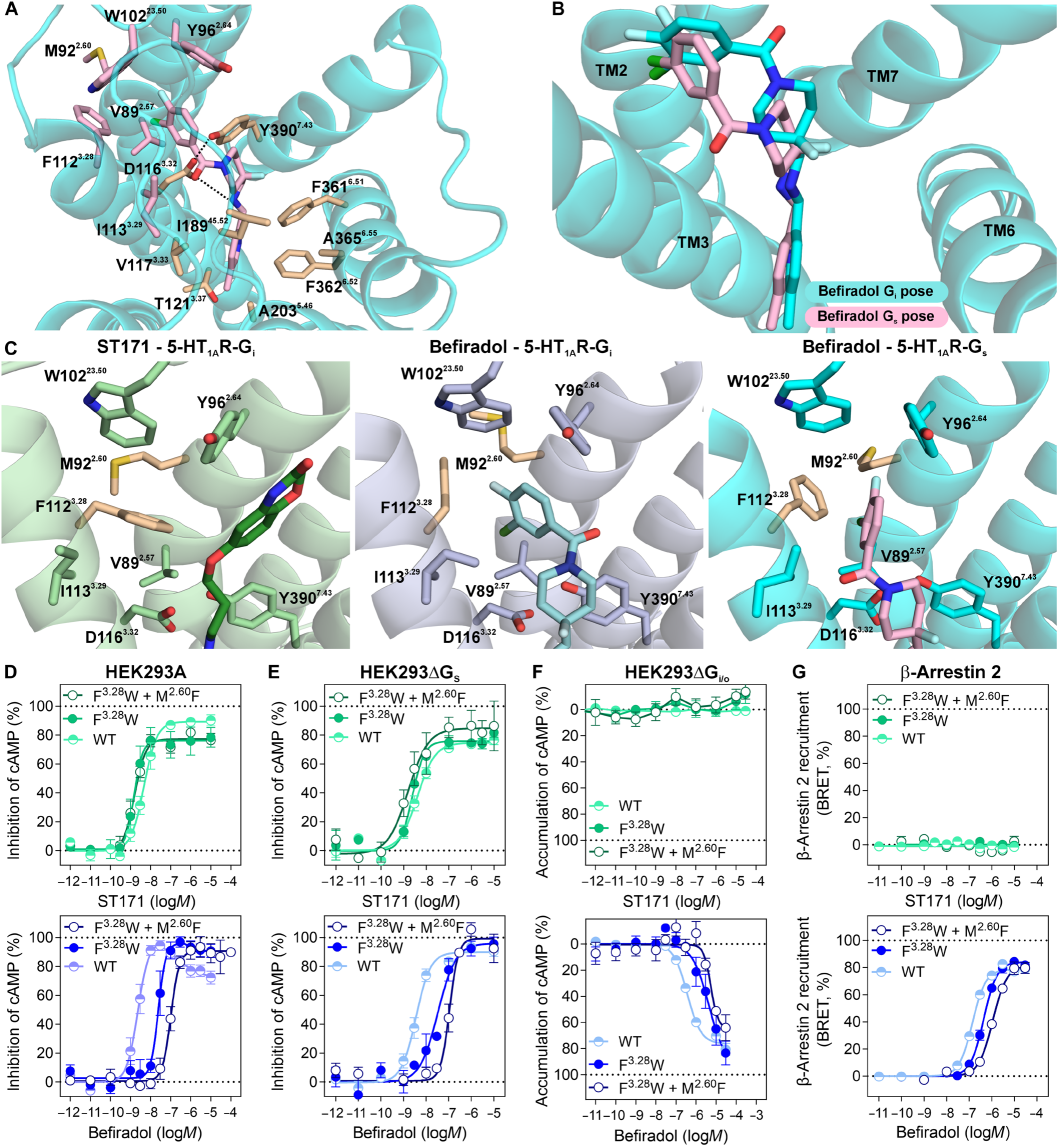
Befiradol distinctly engages the extended binding pocket depending on the coupling partner. (**A**) Top view of the befiradol binding pose within the G_s_ protein–bound 5-HT_1A_R complex and (**B**) comparison to the binding pose in the 5-HT_1A_R-G_i1_ protein complex. (**C**) The extended binding pocket residues F112^3.28^ and M92^2.60^ exhibit different conformations in the cryo-EM structure with ST171 compared to the structures with befiradol bound to 5-HT_1A_R in complex with the G_i_ and G_s_ proteins, respectively. (**D** to **G**) Mutation F112^3.28^W alone and in combination with M92^2.60^F substantially decreases befiradol’s potency for the activation of G_i_- and G_s_-mediated signaling and β-arrestin 2 recruitment at the 5-HT_1A_R, while the signaling profile of ST171 is not affected. For both ligands, the relative efficacies remain unchanged compared to serotonin. Data were obtained with the cAMP GloSensor measuring the inhibition of forskolin-promoted cAMP accumulation in (D) parental HEK293A and (E) HEK cells deficient of G_s_ proteins. (F) In HEK cells deficient in G_i/o_ proteins, an accumulation of cAMP was observed with befiradol. (G) β-Arrestin 2 recruitment was monitored by BRET in HEK293T cells with elevated GRK2 levels. Data are normalized to the maximal response of serotonin (100%) and shown with ±SEM of *n* = 3 to 14 independent experiments.

According to our cryo-EM data, befiradol also adopts a bitopic binding mode in the complex of 5-HT_1A_R with the G_s_ protein, occupying the aforementioned orthosteric and allosteric binding pockets (Fig. 4, F and I). Again, the methyl pyridinyl-substituted aminomethyl moiety of befiradol engages residues V117^3.33^, I167^4.56^, I189^45.52^, A203^5.46^, F361^6.51^, F362^6.52^, and A365^6.55^ in the orthosteric pocket through hydrophobic interactions (Fig. 7A). The distance of the charge-reinforced hydrogen bond between befiradol’s secondary amine and the Cγ of D116^3.32^ remained at 4.0 Å. At the extracellular side, the comparison of the befiradol-bound 5-HT_1A_R-G_i_ and 5-HT_1A_R-G_s_ complexes revealed a 1.8-Å outward movement of TM7, leading to a rearrangement of ECL3 and an increased distance between the carbonyl group of Q97^2.65^ and the indole nitrogen of W358^6.48^. As observed for the befiradol-bound 5-HT_1A_R complex with G_i_, the relative positioning of these residues is unlikely to allow hydrogen bond formation. This is in accordance with this hydrogen bond serving as an indicator of G_i/o_-biased signaling at the 5-HT_1A_R. Substantial differences were observed in how befiradol’s 3-chloro-4-fluorobenzene group reaches the allosteric binding pocket in the complex of 5-HT_1A_R with the G_s_ protein. The carbonyl group shows a flipped orientation (Fig. 7B), now pointing toward TM3 instead of TM7, which requires a rotation of the side chain of I113^3.29^. These changes lead to a differently angled engagement of the 3-chloro-4-fluorobenzene moiety with the allosteric site formed by TM2, TM3, and ECL1. Nevertheless, this moiety addresses the cavity created by the outward movement of the gatekeeper residue F112^3.28^ (Fig. 7C). MD simulations with the befiradol-bound complex of the 5-HT_1A_R coupled to the G_s_ protein (5 × 2 μs) showed higher fluctuations of the ligand (fig. S11C). Furthermore, cluster analysis of the ligand pose throughout the simulations revealed the occurrence of a second binding mode oriented toward TM2 and TM7 in about 16% of the simulation frames and the closure of the cavity by the rotation of the gatekeeper residue F112^3.28^ (fig. S11D), along with the main frames (70%) that are similar to the cryo-EM pose (fig. S11E). These results suggest that the ligand’s pose is not as efficiently stabilized in the complex of the receptor with G_s_ as its less preferred coupling partner. However, most of the conformations still address the befiradol-specific allosteric pocket between TM2, TM3, and ECL1. Additional functional studies with the F112^3.28^W and M92^2.60^F 5-HT_1A_R mutants confirmed the relevance of this subpocket for befiradol’s activity. In agreement with the effects observed in the binding studies, the potency and efficacy relative to sereotonin were highly similar between the 5-HT_1A_R WT, the F112^3.28^W mutant, and the F112^3.28^W + M92^2.60^F mutant for ST171. In contrast, the potency of befiradol substantially decreased for all signaling pathways upon closure of the extended binding pocket by mutation (Fig. 7, D to G, and table S2). The signaling efficacy compared to WT also substantially decreased for serotonin, although this ligand does not address the extended binding pocket (fig. S11, G to I). Moreover, the concentration-response curves for serotonin and befiradol obtained with the GloSensor cAMP assay in the HEK293A cells were almost sigmoid (Fig. 7D and fig. S11F) and we noticed a strong reduction of G_s_-mediated cAMP accumulation elicited by serotonin for the F112^3.28^W and F112^3.28^W + M92^2.60^F mutants when we compared G_s_ signaling to WT 5-HT_1A_R in the HEK293ΔG_i/o_ cells (fig. S11H). These data suggest an overall importance of the extended pocket for 5-HT_1A_R signaling.

## DISCUSSION

Analysis of our in-house library of about 10,000 compounds identified the bitopic ligand ST162. Analoging led us to ST171, which binds to the 5-HT_1A_R with an affinity in the subnanomolar range. Functional and structural investigation of this ligand in complex with the 5-HT_1A_R led to some unanticipated but yet highly interesting findings.

First, ST171 is a strong partial agonist for the activation of Gα_i/o_ that induces inhibition of cAMP accumulation but has only a minimal effect on the recruitment of β-arrestin 2. Intriguingly, 5-HT_1A_R activation by ST171 is functionally selective for the Gα_i/o_ family, with the highest efficacies observed for the Gα_oA_ and Gα_z_ subtypes. In contrast, the earlier drug candidate befiradol and the endogenous agonist serotonin induce accumulation of cAMP at higher ligand concentrations, suggesting competing coupling of Gα_s_ to the 5-HT_1A_R. Preferred coupling to Gα_i/o/z_ proteins over Gα_s_ proteins and/or β-arrestin recruitment was also reported for the promising antinociceptive agents mitragynine pseudoindoxyl and ‘9087, which bind the μ-opioid and the α_2A_ adrenergic receptor, respectively (*41*, *65*). This observation suggests that a preferred activation of the Gα_i/o_ family is particularly relevant to developing both safe and effective pain management drugs in these classes.

Second, our in vivo analyses revealed that ST171 has broad therapeutic potential in both acute and chronic pain mouse models while also showing in vivo activity as an anxiolytic. ST171 is superior, from a therapeutic window perspective, to other 5-HT_1A_R agonists, such as befiradol (*42*). Targeting the 5-HT_1A_R for the treatment of pain has a strong therapeutic rationale, particularly via action at the spinal cord, where its activation is antinociceptive (*27*, *29*). Because of widespread 5-HT_1A_R expression, generating highly selective 5-HT_1A_R agonists devoid of adverse side effects is challenging. Our analysis suggests that motor impairment contributed to befiradol’s antinociceptive effects at higher doses. At lower, nonsedating doses, befiradol did not reverse the mechanical allodynia in a neuropathic pain model. This finding is consistent with previous studies showing a poor efficacy of befiradol against the mechanical hypersensitivity that develops in diabetic or oxaliplatin-induced neuropathy (*25*, *66*). In contrast, we showed that nonsedating doses of ST171 retained strong antinociceptive effects across multiple modalities (heat and mechanical) and in both inflammatory (CFA) and neuropathic (SNI) pain models. We conclude that selective and potent 5-HT_1A_R agonists can be developed to treat pain conditions with different etiologies and good therapeutic windows.

Third, cryo-EM structural studies of the ligand-receptor complexes in the presence of G_i_ or G_s_ proteins revealed that ST171 and befiradol engage the 5-HT_1A_R in different ways and are even dependent on an intracellular coupling partner, which likely accounts for their distinct pharmacology. Although both ligands fill the orthosteric binding pocket as does the endogenous ligand serotonin, these ligands extend further into different exo-sites of the receptor. The binding pose of ST171’s benzoxazinone moiety enables π-π stacking with Y96^2.64^ and stabilizes an interhelical hydrogen bond between the amide group of Q97^2.65^ and the NH group of W387^7.40^, which we did not observe in the befiradol-bound complexes. Our cryo-EM structures, complemented by MD simulations, residue substitutions, and SAR studies, provide insight into how ST171 may stabilize the receptor and limit its flexibility in favor of a conformation that enables G_i/o_ protein binding while showing negligible activation of G_s_ proteins and minimal recruitment of β-arrestin.

Several cautions merit reemphasis: (i) The cryo-EM structures of ST171 and befiradol bound to 5-HT_1A_R in complex with the G_i_ or G_s_ proteins represent only a snapshot of a conformational ensemble that may be differentially stabilized by the individual ligands and signal transducers. Although we identified several differences between the structures and confirmed their stability and relevance in MD simulations and by residue substitutions, it is likely that intermediate states not amenable to structural studies substantially contribute to the receptor/signal transducer recognition process that is critical for the functional selectivity of ST171. (ii) Although ST171 demonstrates efficacious antinociceptive activity in acute and chronic mouse models for pain, a more comprehensive assessment of potential adverse effects (e.g., cardiovascular studies) will be required before the possibility of introducing compounds into the clinic can be considered. (iii) Moreover, while ST171 has a high affinity for the 5-HT_1A_R, it is not entirely (subtype) selective. Specifically, ST171 also engages α_1A_ adrenergic receptors with high affinity and potently activates the 5-HT_1B_R and 5-HT_1D_R as well as dopamine D_2_ and D_4_ receptors. The observed antagonism at adrenergic α_1_ receptors could cause side effects owing to the relaxation of smooth muscles, which could lead to cardiovascular complications, nasal congestion, and miosis (*51*). Therefore, the in vivo α_1_ receptor activity needs to be assessed and reduced during future lead compound optimization. On the other hand, as there is evidence that agonism at the α_1A_ receptor can counteract α_2A_ receptor–mediated analgesia (*67*), antagonism at the α_1A_ receptor would increase ST171-induced analgesia. Furthermore, as triptans that target the 5-HT_1B/D_R are effective in the management of migraine, an action of ST171 at these receptors may have additional benefits. It will therefore be important to understand the contributions of these receptors to the in vivo actions of ST171. The rewarding effects of pain relief are well established and involve a dopaminergic action at the D_2_ receptor in the nucleus accumbens. Conceivably, therefore, the observed D_2_/D_4_ receptor agonism of ST171 may also contribute to its analgesic effects (*68*). Together, these observations highlight the potential very powerful polypharmacological effects of ST171 in the management of pain.

## MATERIALS AND METHODS

### Compounds

Serotonin (catalog no. H9523) and befiradol (catalog no. SML2324) were purchased as hydrochlorides from Sigma-Aldrich (Steinheim, Germany). The Discovery Probe FDA-approved Drug Library (APExBIO, 1971 compounds, 10 mM) was purchased from BioTrend, Cologne, Germany. ST162, ST171, and analogs thereof were synthesized as shown in fig. S1 and described below. Stock solutions for befiradol, ST171, its structural analogs, and the in-house library were prepared in dimethyl sulfoxide at a concentration of 10 mM. The stock solution of serotonin was prepared in water with a concentration of 10 mM. Dilutions were prepared in the buffers indicated for each assay.

#### Chemical synthesis of ST162, ST171, and analogs thereof

General conditions, detailed procedures, and analytical data for the chemical synthesis of ST162, ST171, and analogs thereof are described within the Supplementary Text and fig. S1.

### Plasmids and mutagenesis

If not noted otherwise, the human 5-HT_1A_R and human isoforms of the other GPCRs in pcDNA3.1 (cDNA.org) were used for in vitro experiments. The 5-HT_1A_R mutants Y96^2.64^A, Q97^2.65^A, F112^3.28^W, F112^3.28^W/M92^2.60^F, and W387^7.40^A were generated by polymerase chain reaction using the Quikchange method. For the enzyme fragment complementation–based arrestin recruitment assay, the ARMS2-PK2 sequence was C-terminally fused to the human WT 5-HT_1A_R using the Gibson-assembly method and a Nhe I-HF– and Hind III-HF–digested pCMV-ARMS2-PK2 vector (Eurofins DiscoverX Products). Sequence identity was confirmed by DNA sequencing (Eurofins Genomics).

### Cell lines and cell culture

HEK293T cells were a gift from the Chair of Physiology, FAU Erlangen-Nürnberg. HEK293 cells stably expressing β-arrestin 2 labeled with the enzyme acceptor (HEK293 β-arrestin 2-EA) were obtained from DiscoverX. HEK293A parental cells and KO cell lines HEK293AΔG_s_, HEK293AΔG_i/o_, and HEK293AΔARRB1/B2 have been described previously (*47*–*49*). All cells were cultured in Dulbecco’s modified Eagle’s medium: Nutrient mixture F-12 supplemented with 10% fetal bovine serum, penicillin (100 μg/ml), streptomycin (100 μg/ml), and l-glutamine (0.5 mg/ml) at 37°C and 5% CO_2_ on 10-cm culture dishes. For HEK293AΔG_i/o_, dishes were coated with type I bovine collagen (Corning). For HEK293 β-arrestin 2-EA cells, hygromycin B (150 μg/ml) was added. All cell lines were regularly confirmed to be free of mycoplasma contamination using the MycoAlert Plus detection kit (Lonza).

### Radioligand binding

#### High-throughput screening with radioligand displacement

The starting point for the screening for previously unkown 5-HT_1A_R chemotypes was our in-house library containing ~8000 bioactive compounds generated in our recent GPCR projects and 2000 FDA-approved drugs for addressing targets beyond GPCRs. To create a screening library that comprises a broad variety of different chemotypes, we carefully selected 100 compounds from our GPCR library, representing a set of structurally diverse ligands. The second part of our test library constituted 1971 samples of the DiscoveryProbe FDA-approved drug library (L1021-100, APExBIO, US, purchased from Biotrend, Cologne, Germany) consisting of FDA-approved drugs with known bioactivity and safety data in humans. These test libraries were subjected to an affinity screen for the 5-HT_1A_R, applying a radioligand displacement assay.

In detail, the library samples were provided as 10 mM stocks in dimethyl sulfoxide and were diluted to a 10-fold working solution with binding buffer (50 mM tris, 5 mM MgCl_2_, 1 mM EDTA, and 100 μg/ml bacitracin at pH 7.4). Membranes from HEK293T cells transiently transfected with den cDNA of the human 5-HT_1A_R (cDNA Center, Bloomsburg, PA) and expressing the receptor with a density of *B*_max_ = 2300 fmol/mg protein were incubated at 3 μg protein per well with 0.2 nM [^3^H]WAY600,135 [*K*_D_ (dissociation constant) = 0.070 nM; specific activity: 83 Ci/mmol; Novandi, Södertäljie, Sweden] and the test compounds at a final concentration of 10 nM for 60 min at 37°C. Receptor-bound radioactivity was isolated by filtration and subsequently measured in a scintillation counter as described (*69*). For each compound, two independent single-point measurements were conducted. Specific binding of the radioligand was calculated by normalization of the radioactive counts to the total binding in the presence of buffer (100%) and nonspecific binding (0%) evaluated in the presence of 5 μM unlabeled WAY600,135. The mean displacement of the radioligand as the parameter for the binding affinity of the test compounds was calculated by applying the following equation: Displacement [%] = 100 − Specific binding remaining [%]. In a second round, the screening was continued with 60 analogs of the initial hit ST162 involving the concentration-dependent investigation of ligand binding and the determination of *K*_i_ values as described below for the 5-HT_1A_R, leading to the identification of ST171 based on its high affinity.

#### Radioligand competition

To determine the binding affinities of ST171 and the references serotonin and befiradol to the serotonin receptor subtypes 5-HT_1A_, 5-HT_2A_, and 5-HT_6_, dose-response curves were measured using membranes from HEK293T cells transiently transfected with the appropriate cDNA (all purchased from cDNA Center, Bloomsburg, PA) as described previously (*4*, *69*). Briefly, membranes were incubated with the radioligand (at a concentration similar to the *K*_D_; see Table S5) in binding buffer at final protein concentrations of 2 to 10 μg per well (measured with the method of Lowry) and varying concentrations of the competing ligands for 60 min at 37°C. Nonspecific binding was determined in the presence of the unlabeled ligand (for 5-HT_1A_R and 5-HT_2A_R) or serotonin (5-HT_6_R) at 10 μM. The resulting competition curves were analyzed by nonlinear regression using the algorithms implemented in Prism 9.0 (GraphPad Software, San Diego, CA) to provide IC_50_ values, which were subsequently transformed into *K*_i_ values, applying the equation of Cheng and Prusoff. Mean *K*_i_ values were derived from 3 to 11 experiments, each performed in triplicates. Binding affinities to GPCRs of the dopamine, adrenergic, muscarinergic, opioid, orexin, and neurotensin receptor families were evaluated similarly according to the details listed in table S5.

#### Radioligand saturation

The evaluation of the receptor density in 5-HT_1A_R–expressing HEK293T cells was performed with radioligand saturation experiments using whole cells. Cells were transfected as described for the functional experiments (see the “BRET-based Gα_i1_ activation and β-arrestin 1/2 recruitment” section). After 24 hours, cells were split to be transferred to microplates for functional testing or maintaining cell growth in culture dishes for saturation binding experiments. Cells were harvested at the same time as for the functional tests and resuspended and diluted in binding buffer (see above) to get 30,000 cells per well. Radioligand saturation binding was performed by incubating the cells with [^3^H]WAY600,135 at a concentration in the range of 1 to 2 nM, which represents the saturation concentration for 5-HT_1A_R binding, and was worked up as described above. Receptor density was calculated by transforming the values for specific binding, number of cells, and specific activity of the radioligand to *B*_max_ in [receptors/cell]. Mean values were derived from four to eight single experiments, each done in triplicates.

### In vitro functional studies

#### BRET-based cAMP assay (CAMYEL biosensor)

Activation of G_i/o_ and G_s_ proteins was determined by the detection of intracellular cAMP levels with the BRET-based CAMYEL biosensor (*70*). CHO-K1 cells were transiently transfected with 2 μg of 5-HT_1A_R and 1.0 μg of biosensor per 10-cm culture dish using Mirus TransIT-2020 (Peqlab, Erlangen, Germany) as a transfection reagent. After 24 hours, cells were transferred into white 96-well half-area or 384-well plates (Greiner) in culture medium at a density of 15,000 cells per well (96 well) or 10,000 cells per well (384 well) and maintained at 37°C and 5% CO_2_ for further 24 hours. The medium was exchanged with 20 μl of Dulbecco’s phosphate-buffered saline in which cells were incubated for 30 min before the buffer was exchanged with fresh Dulbecco’s phosphate-buffered saline, followed by an additional incubation for 30 min at 37°C. Coelenterazine h (10 µl, Promega, 5 μM final concentration) was added. Following incubation for 15 min, ligand dilutions and forskolin (3 μM final concentration) were added. After an additional 15 min, the BRET ratio was determined from light emission at 475 and 530 nm with the CLARIOstar plate reader (BMG, Ortenberg, Germany). All responses were normalized to serotonin and analyzed using the algorithms for nonlinear regression (four parameters) in Prism 6.0. For bell-shaped curves, two nonlinear regressions were fit from the global minimum to the global maximum and from the global maximum to the local minimum at high ligand concentrations. To investigate the solely G_s_ protein–mediated response, PTX (25 ng/ml, Sigma-Aldrich, Taufkirchen, Germany) was added during cell seeding on assay plates.

#### GloSensor cAMP assay

G_i/o_ and G_s_ activation by 5-HT_1A_R and the Gα_i_ subunit coupling profile was investigated using the GloSensor cAMP assay and KO cell lines HEK293AΔG_s_, HEK293AΔG_i/o_, and HEK293AΔARRB1/B2 (*47*–*49*) and HEK293T and HEK293A cells. Cells with a confluency of 60 to 80% (10-cm culture dish) were transfected with 0.5 μg (HEK293A, HEK293T, and HEK293AΔARRB1/B2) or 1 μg of 5-HT1_A_R plasmid DNA (HEK293AΔG_s_ and HEK293AΔG_i/o_), 0.5 μg (HEK293A, HEK293T, and HEK293AΔARRB1/B2) or 2 μg (HEK293AΔG_s_ and HEKS293AΔG_i/o_) of Glo-22F cAMP plasmid DNA (Promega), and 2 μg of MOCK DNA (HEK293A, HEK293T, and HEK293AΔARRB1/B2). For the investigation of the Gα_i_ subunit coupling profile, HEK293AΔG_i/o_ cells were cotransfected with 0.5 μg of the plasmid encoding the respective Gα_i_ subunit (Gα_i1_, Gα_i2_, Gα_i3_, Gα_z_, or Gα_oA_; www.cdna.org). All transfections were carried out using Mirus TransIT-293 as the transfection reagent in a 3:1 reagent-to-DNA ratio. After incubation for 24 hours, cells were detached and transferred into CP4 medium (Eurofins DiscoverX Products) at a density of 15,000 cells per well (HEK293T) or 10,000 cells per well (HEK293A, HEK293AΔG_s_, HEK293AΔG_i/o_, and HEK293AΔARRB1/B2) into white-bottom 384-well plates (Greiner) coated with poly-d-lysine (Sigma-Aldrich; for HEK293T, HEK293A, HEK293AΔG_s_, and HEK293AΔARRB1/B2) or type I bovine collagen (HEK293AΔG_i/o_). Plates were incubated at 37°C and 5% CO_2_ for further 24 hours. After incubation, CP4 medium was exchanged with Hanks’ balanced salt solution [with added glucose (1 g/liter) and NaHCO_3_ (0.35 g/liter)] containing GloSensor cAMP Reagent (3%, Promega) and plates were incubated for 60 min. Afterward, ligand dilutions were added, and plates were incubated for 15 min (HEK293T, HEK293A, HEK293AΔG_i/o_, with or without cotransfected Gα_i/o_ subunits, and HEK293AΔARRB1/B2) or 10 min (HEK293AΔG_s_) at room temperature in the dark. For assays in antagonist mode, cells were preincubated with the antagonist for 30 min before agonist solution (~EC_80_) was added and incubation was continued, as described above. Forskolin was added (10 μM final concentration: HEK293T, HEK293A, HEK293AΔARRB1/B2, and HEK293AΔG_i/o_ cotransfected with Gα_i_ subunits; 5 μM final concentration with the additional phosphodiesterase inhibitor RO-20-1724; 100 μM final concentration: HEK293AΔG_s_), and the plates were incubated for further 15 min (HEK293T, HEK293A, HEK293AΔARRB1/B2, and HEK293AΔG_i/o_ cotransfected with Gα_i_ subunits) or 20 min (HEK293AΔG_s_) at room temperature in the dark. Luminescence was determined with the CLARIOstar plate reader (BMG LabTech), or the luminescence was directly measured after incubation with ligand dilutions (HEK293AΔG_i/o_). All responses were normalized to the minimal and maximal effects of serotonin and analyzed using nonlinear regression in Prism 6.0. For bell-shaped curves, two nonlinear regressions were fit from the global minimum to the global maximum and from the global maximum to the local minimum at high ligand concentrations.

#### IP-One accumulation

Determination of Gα_i/o_ and Gα_s_ selective signaling at the 5-HT_1A_R was performed, applying an IP accumulation assay (IP-One HTRF, PerkinElmer, Rodgau, Germany) according to the manufacturer’s protocol and in analogy to previously described protocols (*41*, *71*). HEK293T cells were transiently cotransfected with the cDNA for the 5-HT_1A_R and the hybrid G protein Gα_qi_ or Gα_qs_ (Gα_q_ proteins with the last five amino acids at the C terminus replaced by the corresponding sequence of Gα_i_ or Gα_s_; gift from The J. David Gladstone Institutes, San Francisco, CA) at a ratio of 1:2. On the next day, cells were transferred to 384-well microplates (Greiner, Frickenhausen, Germany) and incubated for further 24 hours. On the day of the experiment, test compounds with concentrations from 0.01 to 100 μM were added to the cells and incubated for 90 min. Accumulation of the second messenger was stopped by the addition of the detection reagents (IP1-d2 conjugate and Anti-IP1cryptate TB conjugate) for 60 min. To measure Gα_qi_-mediated signaling of D_2S_R and Gα_q_-mediated signaling of the 5-HT_2A_R, 5-HT_2C_R, or α_1A_ and α_1B_ adrenergic receptors, HEK293T cells were transfected with the cDNA for the respective receptor (cDNA Center, www.cdna.org) and worked up as described above. For α_1A_ receptor assays in antagonist mode, an additional preincubation step with the antagonist (30 min) was included before the addition of the agonist (30 nM norepinephrine). Time-resolved Förster resonance energy transfer (FRET) was monitored with a CLARIOstar plate reader. FRET ratios were calculated as the ratio of emission intensity of the FRET acceptor (665/10 nm) divided by the FRET donor intensity (620/10 nm). Raw FRET ratios were normalized to buffer conditions (0%) and the maximum effect of norepinephrine or serotonin (100%). Data analysis was performed using the equation for sigmoid concentration-response curves (four-parameter) implemented in GraphPad Prism 9.3 to derive the maximum effect (*E*_max_) and the ligand potency (EC_50_). Three to 11 repeats in duplicate were performed for each test compound.

#### BRET-based Gα_i1_ activation and β-arrestin 1/2 recruitment

For the determination of G protein signaling relative to the receptor density of the 5-HT_1A_R, a BRET biosensor–based assay using Gα_i1-RLucII_ together with Gβ_1_ and Gγ_2-GFP10_ (*41*, *72*) was performed. HEK293T cells were cotransfected with 400, 200, 100, 40, or 10 ng of cDNA for the 5-HT_1A_R and plasmids for G protein activation (100 ng of Gα_i1_, 200 ng of Gβ_1_, and 800 ng of Gγ_2_) using the Mirus TransIT-293 reagent (Peqlab). DNA was complemented to a total amount of 2 μg of DNA per dish with single-stranded DNA (Sigma-Aldrich). After 24 hours, cells were transferred in 96-well plates (Greiner) and grown for further 24 hours. For the experiment, the cell medium was exchanged with phosphate-buffered saline and cells were stimulated with ligands at 37°C for 10 min. Coelenterazine 400a (abcr GmbH, Karlsruhe, Germany) at a final concentration of 2.5 μM was added 5 min before measurement. Gα_i_-mediated signaling by serotonin receptor subtypes 5-HT_1B_R, 5-HT_1D_R, 5-HT_1E_R, and 5-HT_1F_R; α_2A_, α_2B_, and α_2C_ adrenergic receptors; and the dopamine D_4_ receptor was monitored with cells cotransfected with 200 ng (400 ng for 5-HT_1D_R and 5-HT_1F_R) of the appropriate receptor DNA (α_2A_R gift of Y. Du, University of Hong Kong, Shenzhen, CN; others: cDNA Center, www.cdna.org) and the BRET sensor (ratio of receptor/Gα/Gβ/Gγ: 4/1/2/8 or 8/1/2/8 for 5-HT_1D_R and 5-HT_1F_R) using linear polyethyleneimine (PEI; Polysciences, 3:1 PEI-to-DNA ratio) (*73*) according to the protocol described above. 5-HT_1A_R–mediated β-arrestin 2 or β-arrestin 1 recruitment was measured by applying enhanced bystander BRET when cotransfecting 100 ng of 5-HT_1A_R together with 20 ng of β-arrestin 2-_RLucII_ or β-arrestin 1-_RLucII_, respectively, 300 ng of the bystander protein CAAX_rGFP_, and 100 ng of GRK2 with linear PEI in HEK293T cells (*41*, *50*). The assay conditions were identical to the protocol for monitoring Gα_i_ signaling. BRET was monitored on a CLARIOstar plate reader with the appropriate filter sets (donor: 410/80 nm; acceptor: 515/30 nm) and was calculated as the ratio of acceptor emission to donor emission. The BRET ratio was normalized to the effect of buffer (0%) and the maximum effect (100%) of serotonin for 5-HT_1A_R, norepinephrine for the α_2_ adrenoceptors, and quinpirole for D_4_. For each compound, 4 to 17 individual experiments were performed when investigating receptor density–dependent 5-HT_1A_R activation, 3 to 6 for other 5-HT_1_R subtypes, 3 to 14 for α_2_AR activation, 7 to 8 repeats for D_4_R activation, and 4 to 6 experiments when measuring β-arrestin 1 and 2 recruitment upon 5-HT_1A_R stimulation, each done in duplicates.

#### Complementation-based β-arrestin 2 recruitment PathHunter assay

HEK 293 β-arrestin 2-EA cells were transiently transfected with 5-HT_1A_R-ARMS2-PK2 (2 μg/10-cm cell culture plate) using Mirus TransIT-293. After 24 hours, cells were transferred into white 384-well plates (Greiner) in CP4 assay medium at a density of 5000 cells per well and maintained at 37°C and 5% CO_2_ for further 24 hours. On the day of the assay, 5 μl per well of the compounds diluted in phosphate-buffered saline was added and plates were incubated at 37°C for 180 min. For inhibition assays, cells were preincubated with the antagonist for 30 min before the addition of serotonin (~EC_80_). After adding a detection mix (10 μl per well; 3.5% substrate reagent 2, 18% substrate reagent 1, and 78.5% cell assay buffer), plates were incubated at room temperature in the dark for 1 hour. Chemiluminescence was detected with the CLARIOstar plate reader (BMG LabTech). All responses were normalized to the minimal and maximal effects of serotonin and analyzed using the algorithms for nonlinear regression in Prism 6.0.

### Metabolic stability

Microsomal biotransformation reactions were carried out at 37°C in a total volume of 500 μl. The incubation mixture contained 71 μM of compound (rotigotine or ST171) and pooled liver microsomes from male Sprague-Dawley rats (Sigma-Aldrich, M9066) in tris-MgCl_2_ buffer (47 mM tris and 4.8 mM MgCl_2_, pH 7.4; 0.5 mg microsomal protein/ml incubation mixture). Reactions were initiated by the addition of 50 μl of cofactor solution [NADPH (reduced form of nicotinamide adenine dinucleotide phosphate), 1 mM final concentration]. After 0, 15, 30, and 60 min (rotigotine) or 0, 60, 120, and 180 min (ST171), 100 μl of the sample was precipitated by addition to 500 μl of ice-cooled acetonitrile containing 71 μM of an internal standard. After centrifugation (15,000*g*, 3 min), the supernatant was analyzed by high-performance liquid chromatography (HPLC) and HPLC mass spectrometry. Per compound, three individual experiments were performed and nonspecific protein binding was determined in a control reaction without NADPH. Quantification via HPLC was performed using an Agilent 1200 series HPLC system equipped with a diode array detector and a ZORBAX Eclipse XDB-C8 (4.6 by 150 mm, 5 μm) column. A binary solvent system consisting of MeOH/H_2_O + 0.1% HCOOH was used: flow rate of 0.5 ml/min; gradient of 10% MeOH for 3 min, 10 to 100% MeOH in 15 min, 100% MeOH for 6 min, 100 to 10% MeOH in 3 min, and 10% MeOH for 3 min. Liquid chromatography–mass spectrometry analysis of potential metabolites was carried out with a Thermo Scientific Dionex Ultimate 3000 HPLC system (diode array detector) equipped with a Kinetex 2.6u mesh C8 100A (2.1 by 75 mm, 2.6 μm) column using an eluent system consisting of CH_3_CN/0.1% aq. formic acid and 10 to 40% CH_3_CN in 8 min to 90% CH_3_CN in 1 min with a flow rate of 0.3 ml/min. Mass spectrometry was performed in positive mode with a BRUKER amaZon SL spectrometer using electrospray ionization as an ionization source.

### In vivo studies

#### Antinociceptive activity

Animal experiments were approved by the UCSF Institutional Animal Care and Use Committee and were conducted in accordance with the NIH Guide for the Care and Use of Laboratory Animals (protocol no. AN195657). Adult (8 to 10 weeks old) male C56BL/6 mice (stock no. 664) and 5-HT_1A_R KO mice (stock no. 29608) were purchased from the Jackson Laboratory. Mice were housed in cages on a standard 12:12-hour light/dark cycle with food and water ad libitum. Sample sizes were modeled on our previous studies and on studies using a similar approach, which was able to detect significant changes. For all behavioral tests, the experimenter was always blind to treatment. Animals were habituated for 60 min in Plexiglas cylinders and then tested 30 min after intraperitoneal injection of the compounds. ST171 was dissolved in 2HPβCD-saline (1%:99%) and befiradol in Kolliphor HS15-saline-water (20%:40%:40%). For 10 mg/kg doses, compounds were prepared in 2.5 mg/ml solutions of which 100 μl was administered to mice (average weight, 25 g). Hind paw mechanical thresholds were determined with von Frey filaments using the up-down method (*74*). Hind paw thermal sensitivity was measured with a radiant heat source (Hargreaves) or by placing the mice on a 52°C hot plate. For the tail flick assay, sensitivity was measured by immersing the tail into a 50°C water bath. For the ambulatory (rotarod) test, mice were first trained on an accelerating rotating rod, three times for 5 min, before testing with any compound. On the test day, latency to fall from the rod was measured 30 min after injection of the compound. The cutoff was 300 s. Inflammation-induced hyperalgesia was generated by a unilateral intraplantar injection of CFA (20 μl of 50% solution in saline; Sigma-Aldrich) and tested 3 days post-CFA injection. Peripheral nerve injury–induced allodynia was generated by ligating and transecting two of the three branches of the sciatic nerve, leaving the sural nerve intact (*52*). Hypersensitivity was tested 7 days after injury. Statistical tests were performed with GraphPad Prism 9.0.

#### Elevated plus maze

To test the anxiolytic properties of ST171, we used the plus maze assay as previously described (*75*). Briefly, mice received 100 μl of ST171 (10 mg/kg; intraperitoneally) or vehicle (1% cyclodextrin; intraperitoneally) and placed in a Plexiglas cylinder. Thirty minutes later, the mice were placed into the center area of the plus maze, facing a closed arm, and we recorded the time each mouse spent in each arm (closed, center, and open) over a period of 5 min. All statistical analyses were performed with Prism (GraphPad). Data are reported as the means ± SEM. In all experiments, a one-way ANOVA was used to compare the effect of the compounds with their vehicle control.

#### Conditioned place preference

To determine whether ST171 is inherently rewarding, we used the CPP as described previously (*76*). Briefly, on two consecutive days, the mice were placed in the CPP apparatus and allowed to roam freely between the three compartments of the apparatus for 15 min, after which we recorded the time spent in each chamber over the next 30 min (pretest). On days 3 and 4, the mice received an intraperitoneal injection of the vehicle (1% cyclodextrin) or ST171 (10 mg/kg), and then they were placed in a separate cylinder for 30 min and then placed for 30 min in the preferred (vehicle) or nonpreferred (compound) chamber (two conditioning days). On the 5th day (test day), the mice were allowed to again roam freely between the three chambers, and we recorded the time spent in each chamber over 30 min. The CPP score was determined by subtracting the time spent in each chamber on the pretest day from that on the test day (CPP score = Test − Pretest).

### Structural studies

#### Protein engineering

The sequence coding for the human 5-HT_1A_R with an N-terminal hemagglutinin signal sequence (HA), a FLAG tag (DYKDDDDA), an AAA-linker, a 10× histidine tag, and a tobacco etch virus protease cleavage site was codon optimized for expression in insect cells, synthesized by Eurofins Genomics Germany, and subcloned into the pFastBac vector. An L125W^3.41^ point mutation was introduced to increase the expression and stability of the protein. A dominant-negative human Gα_i1_ (dnGα_i1_ with S47N, G203A, E245A, and A326S) and a dominant-negative human Gα_s_ (dnGα_s_ with N54S, A226G, A268E, K271N, D274K, K280R, D284T, and T285I) were used to improve the stability of the nucleotide-free G protein (*77*). Nb35 was gifted by B. K. Kobilka and was modified with an N-terminal pelB sequence and a C-terminal 6× histidine tag followed by an EPEA tag.

#### Expression and purification of the 5-HT_1A_R-G_i1_ protein complex

Recombinant baculoviruses encoding the 5-HT_1A_R construct, dnG_αi1_, human 8× HIS-GSSG-G_β1_, and human G_γ2_ were generated with the Bac-to-Bac baculovirus expression system (Invitrogen). *Spodoptera frugiperda* Sf9 cells (Invitrogen) at a density of ~4 × 10^6^ cells/ml were coinfected with all complex components (receptor construct 1:100, each G protein subunit 1:200 virus–to–cell culture volume) in the presence of 1 μM ST171 or 1 μM befiradol. To increase the receptor expression, cell culture–saturated cholesterol in 5% methyl-β-cyclodextrin was added after 24 hours. Cells were harvested by centrifugation 48 hours postinfection and stored at −80°C. If not mentioned otherwise, the following steps were performed at 4°C. Cell pellets from 2 liters of cell culture were thawed in hypotonic buffer [20 mM Hepes, 10 mM MgCl_2_, 20 mM KCl, and 10 μM ST171 or befiradol, supplemented with a protease inhibitor cocktail (Roche)]. After homogenization with a potter, cells were centrifuged at 25,000*g* for 20 min. Cell pellets were resuspended in hypertonic buffer [20 mM Hepes, 10 mM MgCl_2_, 20 mM KCl, 100 mM NaCl, 5 mM CaCl_2_, and 10 μM ST171 or befiradol, supplemented with a protease inhibitor cocktail (Roche)] and incubated with apyrase (25 mU/ml) for 1 hour at room temperature. The complex was solubilized with 0.5% lauryl-maltose-neopentyl-glycol (LMNG; Anatrace) and 0.01% cholesteryl hemisuccinate (CHS; Anatrace) for 2 hours. Unsolubilized material was removed by centrifugation at 25,000*g* for 20 min before the complex was immobilized by on-column binding to an anti-FLAG antibody resin (Sigma-Aldrich). After washing the resin with 9 column volumes of wash buffer (20 mM Hepes, 5 mM MgCl_2_, 100 mM NaCl, 0.01% LMNG, 0.0025% CHS, and 10 μM ST171 or befiradol), the complex was eluted with a FLAG peptide (200 μg/ml) in wash buffer. The complex was further purified by size exclusion chromatography (SEC) at room temperature using an Äkta go FPLC with a Superdex 200 Increase 10/300 GL column (Cytiva) in SEC buffer (20 mM Hepes, 100 mM NaCl, 0.002% LMNG, 0.0005% CHS, and 10 μM ST171 or befiradol). Eluted fractions were analyzed by SDS–polyacrylamide gel electrophoresis. Fractions containing the complex were pooled and concentrated with a VivaSpin 2 (Cytiva; molecular weight cutoff: 100 kDa) before the sample was aliquoted and flash frozen in liquid nitrogen for storage at −80°C.

#### Expression and purification of Nb35

*Escherichia coli* BL21 (DE3) bacteria were transformed with DNA encoding Nb35 with a C-terminal 6× His tag, and individual clones were selected and transferred to a ventilated culture tube containing 2 ml of LB medium supplemented with ampicillin (50 μg/ml). These cultures were allowed to grow overnight. The subsequent day, the bacterial suspension was transferred to 1 liter of prewarmed terrific broth medium supplemented with 0.4% (v/v) glycerol and grown at 37°C until an optical density at 600 nm value between 0.7 and 1.2 was reached. Induction of bacterial protein expression was then initiated by adding 1 mM isopropyl-β-d-thiogalactopyranoside, followed by overnight incubation at 28°C in the presence of 1 mM MgCl_2_. Cells were harvested by centrifugation and lysed in cold tris-sucrose buffer. After lysis, cell debris was removed by centrifugation at 25,000*g* for 20 min, the supernatant was collected, and the ion concentration was adjusted to 150 mM NaCl, 2 mM MgCl_2_, and 20 mM imidazole. For protein binding, nickel-NTA agarose resin was added to the supernatant for batch binding for 1 hour while stirring gently. After binding, the resin was placed in a chromatography column and washed for 10 column volumes. The nanobody was eluted with buffer containing 250 mM imidazole. Eluted protein was concentrated at 4°C and subjected to SEC. Subsequently, the sample was concentrated, frozen, and stored at −80°C for further usage.

#### Expression and purification of the 5-HT_1A_R-G_s_ protein complex

The purification of the 5-HT_1A_R-G_s_ protein complex was performed similarly to the G_i1_ protein complex with the following exceptions: (i) Buffers used during purification contained 100 μM befiradol; (ii) during apyrase treatment, an estimated 1:1 M ratio of Nb35 was added to the suspension; and (iii) after FLAG elution, the volume was concentrated to ~225 μl and Nb35 was added again in a 1:1.5 M ratio and was incubated overnight before subsequent application to SEC.

#### Western blotting

Protein samples were separated through SDS–polyacrylamide gel electrophoresis without staining. A polyvinylidene difluoride membrane was prepared by activation and equilibration. A wet blotting method was used for protein transfer, and electrophoretic transfer was carried out at a constant voltage of 60 V for 50 min followed by an increase to 90 V for 10 min. The membranes were washed thrice for 5 min with tris-buffered saline with Tween 20 (TBS-T), blocked withTBS-T buffer containing 3% skim milk powder for 1 hour, and then washed again for 5 min with TBS-T buffer to remove excess blocking. The membranes were incubated with mouse anti-His (1:2000, Invitrogen, cat. no. MA121315) and rabbit anti-Gα_s_ (1:6000, Proteintech, cat. no. 10150-2-AP) antibodies in blocking buffer for 1 hour at room temperature to detect proteins containing the respective epitope. Following three 5-min washing steps, membranes were further incubated with a 1:10,000 dilution of horseradish peroxidase–labeled anti-mouse IgG (immunoglobulin G; Proteintech, cat. no. SA00001-1) or anti-rabbit IgG (Proteintech, cat. no. SA00001-2) secondary antibody in blocking buffer for 1 hour at room temperature. Excess antibody was removed by washing the membrane twice for 5 min. After final washing, membranes were incubated with Clarity Western ECL substrate (Bio-Rad) in the dark. Analysis, assessment, and imaging were done using the ChemiDoc gel documentation system with Image Lab software.

#### Cryo-EM sample preparation

Quantifoil grids (UltraAuFoil R 0.6/1, Au 300 mesh, Quantifoil MicroTools, Jena, Germany) were treated by plasma cleaning (Harrick Plasma, model PDC-002, Ithaca, NY) at an air pressure of 29 Pa for 2 min at medium power. For ST171, plasma-cleaned grids were washed with sample buffer to enhance the distribution of particle orientations. The samples were vitrified with a Vitrobot Mark IV (FEI): The chamber of the Vitrobot was equilibrated to a temperature of 4°C with 100% humidity, and 3.5 μl of the sample (2 to 6 mg/ml) was applied to the plasma-cleaned grid mounted in the chamber. This was followed by blotting off excess liquid for 5 s with an uncalibrated blot force of 20 before plunging the grid into liquid ethane. For each sample, batches with four to six grids were vitrified.

#### Cryo-EM imaging and image processing

One to two grids from each batch of 5-HT_1A_R-G_i1_ + ST171 or befiradol were screened in the cryo-EM facility of the Julius-Maximilians-Universität Würzburg (Titan-Krios G3, X-FEG, 300 kV, Falcon III direct electron detector): Movies were acquired with EPU at a magnification of 75,000× (pixel size at the specimen level: 1.06 Å) in counting mode. The total exposure was 80 e^−^/Å^2^ fractionated over 40 frames (exposure time: 80 s). The movie frames were motion corrected, dose weighted, and averaged with MotionCor2 (*78*) as part of the preprocessing during the data acquisition. The averages were imported to CryoSPARC version 3.5-4.1 (*79*) and further processed to obtain two-dimensional (2D) class averages, 3D maps, and variability components. The samples were selected for full-data acquisition on the basis of particle density, 2D class averages showing secondary structure elements, and low heterogeneity in the variability analysis.

The full datasets for the 5-HT_1A_R-G_i1_ complexes were obtained from different grids of the same batches with a Titan Krios3 G4 with C-FEG, Selectris X energy filter and Falcon IVi direct electron detector at the EMBL Imaging Center in Heidelberg (Germany): Movies were recorded in counting mode at a magnification of 165,000× (pixel size at the specimen level: 0.73 Å) with zero-loss imaging (energy filter slit width: 10 eV) and a total exposure of 80 e^−^/Å^2^ (exposure time: 3.7 s). Semiautomated data acquisition was done with SerialEM with one movie per hole and up to nine holes per stage position with a targeted underfocus between 0.6 and 1.6 μm. The movies were recorded in EER format and were motion corrected, dose weighted, and averaged with MotionCor2 parallel to the image acquisition with the “relion_it.py” script of Relion 4 (*80*). The movie averages were imported to CryoSPARC 4.0-4.1 for further processing. The final maps were autosharpened in Phenix (*81*) for subsequent model building and structural refinement.

The datasets of the befiradol-bound 5-HT_1A_R in complex with G_s_ were acquired in the cryo-EM facility of the Julius-Maximilians-Universität Würzburg (Titan-Krios G3, X-FEG, 300 kV, Falcon IVi, Selectris energy filter) with EPU in EER format at a magnification of 130,000× (pixel size at the specimen level: 0.95 Å) with zero-loss imaging (slit width: 5 eV). The total exposure was 70 e^−^/Å^2^. The movies were preprocessed in a life session with CryoSPARC, and processing was continued with CryoSPARC. The final sorted particle stack was exported to Relion 5 using pyem. Particle orientations of this subset were iteratively refined with 3D autorefine using blush regularization.

#### Structure determination and refinement

The initial 5-HT_1A_R model [Protein Data Bank (PDB): 7E2Z] (*8*) was a rigid body fit into cryo-EM maps using Chimera (*82*) software. The 5-HT_1A_R-ST171 model was manually adjusted in COOT (*83*) and paired with iterative real-space refinement in Phenix (*84*). Model quality was evaluated using MolProbity. Figures of the structure and model were prepared in Chimera (*82*) and Pymol (https://pymol.org/2/).

### MD simulations

The simulations are based on the here-reported cryo-EM structures of the 5-HT_1A_R in complex with ST171 and befiradol or based on the 5-HT_1A_R in complex with serotonin (PDB: 7E2Y) (*8*). Coordinates were prepared by removing the Gα/β/γ subunits of the heterotrimeric G protein. The cholesterol between TM1 and TM7 was retained for all complexes because previous studies indicated its effect on the regulation of serotonin receptors (*8*). Because of the importance of water-mediated interactions in the binding mode of ST171, water molecules determined in the cryo-EM structure of the ST171-bound complex were retained in the preparation of the ST171-bound system. Missing residues in ECL2 were modeled using MODELLER (*85*). The long and flexible ICL3 (intracellular loop 3) was modeled in the binary complex using the first five amino acid residues after TM5 and the first five amino acid residues before TM6. For the ternary complex, ICL3 was not modeled. The open termini, as well as the terminal residues at the intracellular ends of TM5 and TM6 for the ternary complexes, were capped with an acetyl group or *N*-methylamide. All titratable residues were left in their dominant protonation state at pH 7.0 except D82^2.50^. Parameter topology and coordinate files were generated using the tleap module of the AMBER22 program package (*86*). The created GPCR models were energy minimized using the PMEMD module of AMBER22 by applying 500 steps of steepest descent followed by 4500 steps of conjugated gradient. All files were converted to GROMACS input files using ParmEd. The protein structures were then aligned to the Orientation of Proteins in Membranes structure of the 5-HT_1A_R in complex with serotonin (PDB: 7E2Y). Each complex was inserted into a solvated and preequilibrated membrane of dioleoyl-phosphatidylcholine (DOPC) lipids using the GROMACS tool g_membed (*87*). Water molecules were replaced by sodium and chloride ions to result in neutral and physiological systems with 0.15 M NaCl. Final dimensions of the simulation systems were about 80 Å by 80 Å by 100 Å, containing ~65,000 atoms, including ~154 DOPC molecules, ~13,200 water molecules, ~58 sodium, and ~70 chloride ions. For all simulations, the general AMBER force field (GAFF2) was used for the ligands, the Lipid14 force field for the DOPC and cholesterol molecules, and ff14SB for the protein residues. The SPC/E water model was applied. Parameters for befiradol, ST171, and serotonin were assigned using antechamber (*86*). The structures of all ligands were optimized by Gaussian 16 using the B3LYP functional and the 6-31G(d) basis set. Charges were calculated using the HF functional and the 6-31G(d) basis set. Subsequently, atom point charges were assigned according to the RESP procedure. A formal charge of +1 was defined for all ligands. Simulations were performed using GROMACS 2023.0 (*88*). Each simulation system was energy minimized and equilibrated in the NVT ensemble at 310 K for 1 ns followed by the NPT ensemble at 310 K and 1 atm for 1 ns with harmonic restraints of 10.0 kcal mol^−1^ on the protein and ligands. In the NVT ensemble, the velocity-rescale thermostat was used. In the NPT ensemble, the Berendsen barostat, the velocity-rescale thermostat, a surface tension of 22 dyn cm^−1^, and a compressibility of 4.5 × 10^−5^ bar^−1^ were applied. Subsequently, the system was further equilibrated in the NPT ensemble at 310 K and 1 atm for 25 ns with restraints on the protein backbone and ligand atoms in which the restraints were reduced every 5 ns in a stepwise fashion to 10.0, 5.0, 1.0, 0.5, and 0.1 kcal mol^−1^ Å^−2^. Production simulations were performed for a duration of 2 μs. Five replicas were initiated for each ligand in both the binary and ternary complexes. Bond lengths to hydrogen atoms were constrained using the LINCS algorithm. Periodic boundary conditions were applied. A cutoff of 12.0 Å was used for Lennard-Jones interactions and the short-range electrostatic interactions. Long-range electrostatics were computed using the particle mesh Ewald method with a fourth-order interpolation scheme and fast Fourier transform grid spacing of 1.6 Å. A continuum model correction for energy and pressure was applied to long-range van der Waals interactions. The equations of motion were integrated with a time step of 2 fs. During the production simulation of the ternary complexes, all residues within 5 Å of the G protein interface were restrained to the initial structure using 5.0 kcal mol^−1^ Å^−2^ harmonic restraints applied to nonhydrogen atoms. Using such restraints instead of the intracellular binding partner reduces the overall system size, enabling faster simulations while ensuring that the receptor maintains a conformation corresponding to the ternary complex throughout the simulation.

Multiple walker metadynamics simulations were conducted using GROMACS 2021.4 software patched with PLUMED (*89*). Starting frames for the 32 individual walkers were taken from the trajectories of three independent 2-μs runs of unbiased MD simulations of the befiradol-bound binary complex model after the dismission of its ligand. The dihedral angle between the N, C_α_, C_β_, and C_γ_ atoms of the F112^3.28^ side chain was selected as a collective variable and was subject to a bias potential, which enabled a reconstruction of the free energy surface. Gaussian hills with a height of 0.239 kcal mol^−1^ were applied every 1.0 ps. The hill width was set to 0.1 rad. Rescaling of the Gaussian function was done with a bias factor of 5.

Analysis of the trajectories was performed using Visual Molecular Dynamics and CPPTRAJ. Plots were created using Matplotlib 3.6.3 and Seaborn 0.12.

### Statistical analysis

The number of independent repeats for each experiment is indicated in the figure legends. If not noted otherwise, data are indicated as the means with error bars depicting the SEM of biological replicates. There were no preestablished sample exclusion criteria, and data points were not excluded from the analysis. Statistical analysis of the in vivo assays was performed with the tests implemented in GraphPad Prism, as indicated in the legend of Fig. 3 and fig. S6.

## Supplementary Materials

### This PDF file includes:

Supplementary Text Figs. S1 to S11 Tables S1 to S5 References
