# Supplementary material for: Discovery of a functionally selective serotonin receptor (5-HT1AR) agonist for the treatment of pain
Source: Sci Adv. 2025 Jun 18;11(25):eadv9267. doi: 10.1126/sciadv.adv9267 (PMC12175894; doi:10.1126/sciadv.adv9267)
Supplement: Supplementary file 1 — Supplementary Text Figs. S1 to S11 Tables S1 to S5 References [file sciadv.adv9267_sm.pdf]

Science Advances
AAAS

Supplementary Materials for

Discovery of a functionally selective serotonin receptor (5-HT_{1A}R) agonist for the treatment of pain

Annika Ullrich *et al.*

Corresponding author: Peter Gmeiner, peter.gmeiner@fau.de; Dorothee Weikert, dorothee.weikert@fau.de; Bettina Böttcher, bettina.boettcher@uni-wuerzburg.de; Allan I. Basbaum, allan.basbaum@ucsf.edu

Sci. Adv. **11**, eadv9267 (2025)
DOI: 10.1126/sciadv.adv9267

This PDF file includes:

Supplementary Text
Figs. S1 to S11
Tables S1 to S5
References

Supplementary Text

<u>Chemical Synthesis of ST162, ST171 and analogs thereof</u>

The synthesis of **ST162** (fig. S1A) started from 4-methoxy-2-nitroaniline [**1**]. Catalytic hydrogenation furnished the respective diamine **2**, which was cyclized with CDI to give the benzimidazolone **3**. After cleavage of the methyl ether with BBr_3, the resulting phenol **4** was alkylated to give the respective bromopropyl ether **5**, before nucleophilic substitution with 2-methoxyphenoxyethylamine (*90*) in the final step. The synthesis of **ST171** and its analogs **TA12**, **TA13**, **TA14** and **TA48** (fig. S1B) started from 2*H*-benzo[*b*][1,4]oxazin-3(4*H*)-one (**6**), which was oxidized with [bis(trifluoroacetoxy)iodo] benzene to give the respective hydroxyl analogue **7**. Alkylation with different dibromoalkanes afforded the bromoalkyl ethers **8**, **9** and **10**. In the final step, nucleophilic substitution with 2-methoxyphenoxyalkylamine or 2-methylthiophenoxyethylamine afforded the desired secondary amines.

General conditions

Reagents and dry solvents were purchased in the highest available purity grade and used without further purification. MS was run on a BRUKER ESQUIRE 2000 ion-trap mass spectrometer or on a BRUKER amaZon SL mass spectrometer using ESI as the ionization source. HR-MS was performed on a Bruker Daltonic microTOF II focus TOF-MS spectrometer using ESI as the ionization source. NMR spectra were obtained on a Bruker Avance 360 or a Bruker Avance 600 spectrometer at 300 K. ^1H and ^{13}C chemical shifts are given in ppm (δ) relative to TMS in the solvents indicated. IR spectra were performed on a Jasco FT/IR 4100 spectrometer using a film of substance on a NaCl plate. Purification by flash column chromatography was conducted using silica gel 60 (40-63 µm mesh, Merck, Germany) and eluents as binary mixtures with the volume ratios indicated. Purification with preparative HPLC was conducted on an Agilent 1100 preparative series HPLC system combined with a VWD detector. As an HPLC column, a MACHEREY-NAGEL Varioprep VP 250/10 Nucleodur C18 HTec (10 x 250 mm, 5 µm) was used (flow rate 4 mL/min, λ = 254 nm). TLC analyses were performed using Merck 60 F_{254} aluminum sheets and analyzed by UV light (254 nm). Additionally, a ninhydrin spray reagent was used for the detection of aminergic compounds. Analytical HPLC was performed on an AGILENT 1200 series HPLC system employing a DAD detector. As HPLC column, a ZORBAX ECLIPSE XDB-C8 (4.6 x 150 mm, 5 µm) was used. The purity of all test compounds was determined to be >96% using HPLC with the following binary solvent systems:

System 1: MeOH/H2O + 0.1% HCOOH, flow 0.5 ml/min; gradient: 10% MeOH for 3 min, 10% - 100% MeOH in 15 min, 100% MeOH for 6 min, 100% - 10% MeOH in 3 min, 10% MeOH for 3 min.

System 2: ACN/H2O + 0.1% TFA, flow 0.5 ml/min; gradient: 10% ACN for 3 min, 10% - 90% ACN in 15 min, 90% ACN for 6 min, 90% - 10% ACN in 3 min, 10% ACN for 3 min.

4-Methoxybenzene-1,2-diamine [**2**]

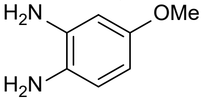

A suspension of 4-methoxy-2-nitroaniline (1.00 g, 5.95 mmol) and palladium on charcoal 10% wt. (100 mg) in methanol (10 mL) was stirred for 24 h under hydrogen atmosphere with light protection at room temperature. The suspension was filtered through celite and the solvent was evaporated. The crude product was purified by flash chromatography (CH_2Cl_2/methanol 99:1 + 0.2% NH_3) to give **2** as brown solid (645 mg, 78% yield). ESI *m/z* 138.9 $[M+1]^+$; IR (NaCl) ν (cm^{-1}): 3338, 3288, 2954, 2925, 1628, 1515, 1451, 1295, 1248, 1202, 1166, 1032, 955; ^1H-NMR ($CDCl_3$, 600 MHz) δ (ppm): 3.24 (s(b), 4H), 3.73 (s, 3H), 6.26 (dd, J = 8.4, 2.8 Hz, 1H), 6.32 (d, J = 2.8 Hz, 1H), 6.64 (d, J = 8.4 Hz, 1H); ^{13}C-NMR ($CDCl_3$, 360 MHz) δ (ppm): 55.53, 102.93, 104.05, 118.33, 127.3, 137.07, 154.6.

5-Methoxy-1,3-dihydro-2H-benzo[d]imidazol-2-one [**3**]

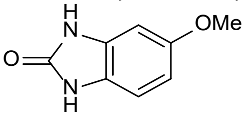

A solution of **2** (400 mg, 2.89 mmol) and 1,1'-carbonyldiimidazole (516 mg, 3.18 mmol) in DMF (11.4 mL) was stirred at 80 °C for 3 h. After the reaction has cooled to room temperature, the solution was concentrated in vacuo, 1 M HCl solution was added and the aqueous phase was extracted with ethyl acetate. The combined organic layers were washed with brine, dried over Na_2SO_4 and evaporated. The crude product was purified by flash chromatography (CH_2CL_2/methanol 98:2) to give **3** as a light brown solid (325 mg, 68% yield). ESI-MS *m/z* 164.9 $[M+1]^+$; IR (NaCl) ν (cm^{-1}): 3397, 3005, 2863, 1759, 1616, 1506, 1366, 1195, 1156, 1106, 1022, 887, 776; ^{1}H-NMR (DMSO-d_6, 600 MHz) δ (ppm): 3.69 (s, 3H), 6.49 – 6.52 (m, 2H) 6.8 (d, J = 8 Hz, 1H), 10.36 (s, 1H), 10.5 (s, 1H); ^{13}C-NMR (DMSO-d_6, 600 MHz) δ (ppm): 55.39, 95.25, 106.04, 108.67, 123.54, 130.48, 154.31, 155.61.

5-Hydroxy-1,3-dihydro-2H-benzo[d]imidazol-2-one [**4**]

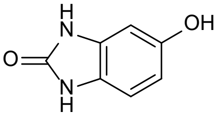

A solution of **3** (211 mg, 1.29 mmol) in CH_2Cl_2 (5.3 mL) was cooled to – 78 °C and put under argon atmosphere. 1 M BBr_3 solution in CH_2Cl_2 (3.85 mL, 3.85 mmol) was added dropwise over 20 minutes. The mixture was kept at – 78 °C for 1 h, then allowed to warm to room temperature and stirred for another 2 h. The reaction was quenched by slowly adding satured $NaHCO_3$ solution at -10 °C and pH was kept < 7. The mixture was extracted with ethyl acetate. The combined organic layers were washed with brine, dried over Na_2SO_4 and evaporated to give **4** as a reddish solid (113 mg, 59% yield). ESI-MS *m/z* 150.9 $[M+1]^+$; IR (NaCl) ν (cm^{-1}): 3353, 3096, 2998, 1727, 1598, 1479, 1366, 1241, 1023, 883, 815, 760, 611; ^{1}H-NMR (DMSO-d_6, 600 MHz) δ (ppm): 6.32 (dd, J = 8.3, 2.3 Hz, 1H), 6.37 (d, J = 2.3 Hz, 1H), 6.67 (d, J = 8.3 Hz, 1H), 8.85 (s, 1H), 10.19 (s, 1H), 10.30 (s, 1H); ^{13}C-NMR (DMSO-d_6, 600 MHz) δ (ppm): 96.39, 107.07, 108.67, 122.21, 130.42, 151.92, 155.55.

5-(3-Bromopropoxy)-1,3-dihydro-2H-benzo[d]imidazol-2-one [**5**]

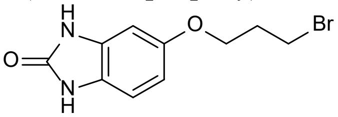

To a suspension of **4** (106 mg, 0.71 mmol) and anhydrous K_2CO_3 (117 mg, 0.85 mmol) in ethanol (1 mL) was added 1,3-dibromopropane (0.18 mL, 1.77 mmol) dropwise and the resulting mixture was refluxed for 3.5 h. After the reaction has cooled to room temperature, water was added and the aqueous phase was extracted with ethyl acetate. The combined organic layers were washed with brine, dried over Na_2SO_4 and evaporated. The crude product was purified by flash chromatography (CH_2Cl_2/methanol 96:4 + 0.2% NH_3) to give **5** as a light yellow solid (57 mg, 30% yield). ESI-MS *m/z* 271.2 $[M+1]^+$; IR (NaCl) ν (cm^{-1}): 3434, 3235, 3024, 2925, 1754, 1708, 1642, 1510, 1468, 1254, 1170, 1025, 767; ^{1}H-NMR (DMSO-d_6, 600 MHz) δ (ppm): 2.18 – 2.23 (m, 2H), 3.66 (t, J = 6.6 Hz, 2H), 4.01 (t, J = 6 Hz, 2H), 6.51 – 6.55 (m, 2H), 6.79 (d, J = 8.4 Hz, 1H), 10.36 (s, 1H), 10.49 (s, 1H); ^{13}C-NMR (DMSO-d_6, 600 MHz) δ (ppm): 31.38, 31.93, 65.93, 96.16, 106.92, 108.68, 123.8, 130.45, 153.32, 155.58.

5-(3-((2-(2-Methoxyphenoxy)ethyl)amino)propoxy)-1,3-dihydro-2H-benzo[d]imidazol-2-one x TFA [**ST162**]

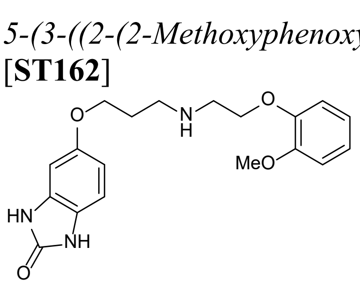

A solution of **5** (44 mg, 0.16 mmol), 2-methoxyphenoxyethylamine (69 mg, 0.41 mmol) and KI (27 mg, 0.16 mmol) in CH_3CN (2 mL) was refluxed under a nitrogen atmosphere for 4.5 h. After the reaction has cooled to room temperature, water was added and the aqueous phase was extracted with CH_2Cl_2. The combined organic layers were washed with brine, dried over Na_2SO_4 and evaporated. The crude product was purified by preparative HPLC (10-70% CH_3CN/ 0.1% aq. trifluoroacetic acid) to give **ST 162** as a white solid (24 mg, 31% yield). ESI-MS *m/z* 358.5 $[M+1]^+$; HR-MS calculated: 358.17613, found: 358.17578; IR (NaCl) ν

(cm^{-1}): 3411, 2962, 2921, 2848, 1740, 1636, 1503, 1369, 1256, 1205, 1027, 809, 613; ^{1}H-NMR (DMSO-*d_6*, 360 MHz) δ (ppm): 2.04 – 2.14 (m, 2H), 3.15 – 3.27 (m, 2H), 3.76 (s, 3H, OCH_3), 4.01 (t, *J* = 6 Hz, 2H), 4.24 (t, *J* = 4.8 Hz, 2H), 6.49 – 6.57 (m, 2H), 6.81 (d, *J* = 8.3 Hz, 1H) 6.86 – 7.06 (m, 4H), 8.99 (s(b), 2H), 10.39 (s, 1H), 10.54 (s, 1H); ^{13}C-NMR (DMSO-*d_6*, 360 MHz) δ (ppm): 25.62, 44.96, 46.1, 55.46, 65.02, 65.4, 96.14, 106.89, 108.71, 112.38, 115.03, 120.73, 122.36, 123.83, 130.47, 147.07, 149.48, 153.26, 155.65; HPLC system 1: t_R = 14.9 min, purity: 99%; system 2: t_R = 9.7 min, purity: 99%.

6-Hydroxy-2H-benzo[b][1,4]oxazin-3(4H)-one [**7**]

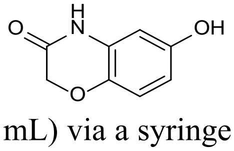

To a refluxing solution of 2*H*-benzo[*b*][1,4]oxazin-3(4*H*)-one (200 mg, 1.34 mmol) in trifluoroacetic acid (3.6 mL) was added a solution of [bis(trifluoroacetoxy)iodo]benzene (692 mg, 1.61 mmol) in trifluoroacetic acid (7.2 mL) via a syringe at once and the mixture was stirred for 10 min. After the reaction has cooled to room temperature, it was transferred slowly in an ice cold saturated $NaHCO_3$ solution. The pH was adjusted to 4 – 5 with 1 M HCl and the aqueous phase was extracted with ethyl acetate. Combined organic layers were washed with brine, dried over Na_2SO_4 and evaporated. The crude product was purified by flash chromatography (hexane/ethyl acetate 4:1) to give **7** as an off white solid (100 mg, 45% yield). ESI-MS *m/z* 165.8 [M+1]$^+$; IR (NaCl) ν (cm^{-1}): 3152, 3055, 2915, 1683, 1620, 1498, 1416, 1309, 1203, 1047, 847, 818, 725; ^{1}H-NMR (DMSO-*d_6*, 600 MHz) δ (ppm): 4.42 (s, 2H), 6.27 (dd, *J* = 8.7, 2.8 Hz, 1H), 6.36 (d, *J* = 2.8 Hz, 1H), 6.72 (d, *J* = 8.7 Hz, 1H), 9.13 (s, 1H, benzo[*b*]oxazinone-OH), 10.52 (s, 1H).

6-(2-Bromoethoxy)-2H-benzo[b][1,4]oxazin-3(4H)-one [**8**]

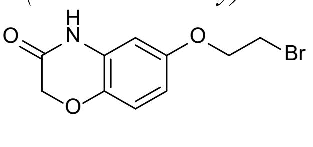

Compound **8** was synthesized according to the protocol of **5** by adding 1,2-dibromoethane (90 µl, 1.0 mmol) to a mixture of **7** (68 mg, 0.41 mmol) and K_2CO_3 (57 mg, 0.41 mmol) in ethanol (5 mL) and let it stir under reflux conditions for 2 h. After the reaction has cooled to room temperature water was added and the mixture was extracted with ethyl acetate. The combined organic layers were washed with brine, dried over Na_2SO_4 and evaporated. The crude product was purified by flash chromatography (*n*-hexane/ethyl acetate 15:3) to give 8 as white solid (71 mg, 63% yield). ESI-MS *m/z* 272.4; 274.4 [M+H]$^+$; ^{1}H-NMR (400 MHz, DMSO-*d_6*) δ 10.64 (s, 1H), 6.87 (d, *J* = 8.6 Hz, 1H), 6.52 (dd, *J* = 8.6, 2.9 Hz, 1H), 6.49 (d, *J* = 2.7 Hz, 1H), 4.49 (s, 2H), 4.24 – 4.20 (m, 2H), 3.78 – 3.74 (m, 2H); ^{13}C-NMR (101 MHz, DMSO-*d_6*) δ 165.76, 154.06, 137.86, 128.27, 117.05, 108.69, 103.17, 68.74, 67.32, 32.01.

6-(3-Bromopropoxy)-2H-benzo[b][1,4]oxazin-3(4H)-one [**9**]

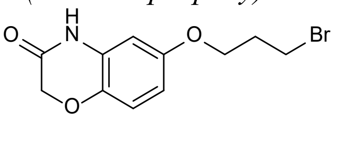

Compound **9** was prepared according to the protocol of **5** by refluxing a suspension of **7** (65 mg, 0.39 mmol), anhydrous K_2CO_3 (65 mg, 0.47 mmol) and 1,3-dibromopropane (0.10 mL, 0.98 mmol) in ethanol (0.8 mL) for 2 h. The crude product was purified by flash chromatography (hexane/ethyl acetate 2:1) to give **9** as a white wax (67 mg, 59% yield). ESI-MS *m/z* 285.9 [M+1]$^+$; IR (NaCl) ν (cm^{-1}): 3214, 3062, 2956, 1695, 1609, 1516, 1387, 1316, 1265, 1211, 1049, 898, 758; ^{1}H-NMR (DMSO-*d_6*, 600 MHz) δ (ppm): 2.18 – 2.23 (m, 2H), 3.64 (t, *J* = 6.5 Hz, 2H), 3.98 (t, *J* = 6 Hz, 2H), 4.48 (s, 2H), 6.48 (d, *J* = 2.9 Hz, 1H), 6.5 (dd, *J* = 8.7, 2.9 Hz, 1H), 6.85 (d, *J* = 8.7 Hz, 1H), 10.6 (s, 1H); ^{13}C-NMR (DMSO-*d_6*, 600 MHz) δ (ppm): 31.25, 31.76, 65.72, 66.82, 102.46, 108.20, 116.55, 128.02, 137.33, 153.63, 165.21.

6-(4-Bromobutoxy)-2H-benzo[b][1,4]oxazin-3(4H)-one [**10**]

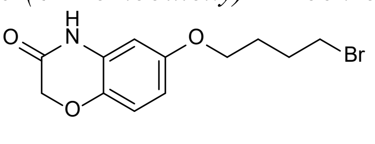

To a suspension of **7** (68 mg, 0.41 mmol) and anhydrous K_2CO_3 (68 mg, 0.49 mmol) in ethanol (5 mL) was added 1,4-dibromobutane (0.12 mL, 1.0 mmol) dropwise and the resulting mixture was refluxed for 2 h. After cooling to room temperature water was added and the aqueous phase was extracted with ethyl acetate. The organic layers were combined and washed with brine. After drying over

Na_2SO_4 the organic solvent was evaporated and the crude product was purified by flash chromatography (*n*-hexane/ethyl acetate 4:1) to give **10** as an off-white solid (66 mg, 53% yield). ESI-MS *m/z* 300.9; 302.9 $[M+H]^+$; ^{1}H-NMR (400 MHz, DMSO-*d6*) δ 10.61 (s, 1H), 6.85 (d, *J* = 8.6 Hz, 1H), 6.48 (dd, *J* = 8.6, 2.9 Hz, 1H), 6.46 (d, *J* = 2.7 Hz, 1H), 4.48 (s, 2H), 3.91 (t, *J* = 6.2 Hz, 2H), 3.59 (t, *J* = 6.7 Hz, 2H), 1.98 – 1.90 (m, 2H), 1.84 – 1.73 (m, 2H); ^{13}C-NMR (101 MHz, DMSO) δ 165.28, 153.84, 137.21, 128.02, 116.54, 108.13, 102.47, 67.03, 66.86, 34.87, 29.08, 27.42.

6-(3-((2-(2-Methoxyphenoxy)ethyl)amino)propoxy)-2H-benzo[b][1,4]oxazin-3(4H)-one x TFA [**ST171**]

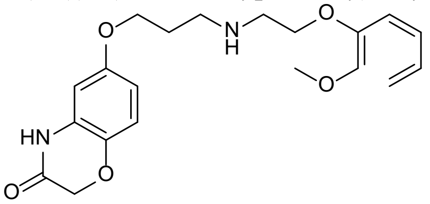

A solution of **9** (66 mg, 0.23 mmol), 2-methoxyphenoxyethylamine (96 mg, 0.58 mmol) (*90*), and KI (58 mg, 0.35 mmol) in CH_3CN (3.3 mL) was refluxed under nitrogen atmosphere for 5 h. After the reaction has cooled to room temperature, water was added and the aqueous phase was extracted with CH_2Cl_2. The combined organic layers were washed with brine, dried over Na_2SO_4 and evaporated. The crude product was purified by preparative HPLC (10-100% methanol/ 0.1% aq. trifluoroacetic acid) to give **ST171** as a white solid (64 mg, 74% yield). ESI-MS *m/z* 373.1 $[M+1]^+$; HR-MS calculated: 373.17580, found: 373.17540; IR (NaCl) ν (cm^{-1}): 3431, 3065, 2922, 1685, 1610, 1507, 1396, 1326, 1256, 1203, 1126, 1054, 797; ^{1}H-NMR ($CDCl_3$, 600 MHz) δ (ppm): 2.24 – 2.31 (m, 2H), 3.41 (t, *J* = 5.8 Hz, 2H), 3.49 (t, *J* = 4.7 Hz, 2H), 3.80 (s, 3H), 4.08 (t, *J* = 5.2 Hz, 2H), 4.29 – 4.34 (m, 4H), 6.40 – 6.45 (m, 2H), 6.76 (d, *J* = 8.6 Hz, 1H), 6.88 – 6.97 (m, 3H), 7.01 – 7.06 (m, 1H), 8.73 (s, 1H), 9.85 (s(b), 2H); ^{13}C-NMR ($CDCl_3$, 600 MHz) δ (ppm): 25.76, 46.53, 47.55, 55.75, 65.72, 66.43, 67.24, 103.21, 108.8, 112.3 116.79, 116.96, 121.47, 123.8, 127.01, 138.04, 146.66, 149.91, 153.46, 165.6; HPLC system 1: t_R = 16.5 min, purity: 98%; system 2: t_R = 11.6 min, purity: 98%.

3-(2-Methoxyphenoxy)propyl-1-amine [**11**]

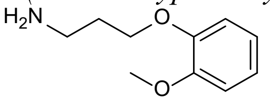

A mixture of 2-methoxyphenol (2.98 mL, 26.9 mmol), acrylonitrile (17.9 mL, 270 mmol), K_2CO_3 (187 mg, 1.35 mmol), and *tert*-butanol (0.24 mL, 2.7 mmol) was heated to reflux under argon atmosphere. After 4 h K_2CO_3 (187 g, 1.35 mmol) was added, and the reaction was stirred for further 48 h under reflux conditions. After cooling to room temperature phosphoric acid 85% (0.12 mL, 2.2 mmol) was added dropwise and the reaction was stirred for further 30 min. Subsequently, ethyl acetate was added, and the mixture was washed with 1 M NaOH solution. The resulting aqueous phase was extracted with ethyl acetate again and the combined organic layers were washed with 1 M NaOH and 10% phosphoric acid solution, dried over Na_2SO_4 and evaporated to give 3-(2-methoxyphenoxy)propanenitrile as light brown solid (2.49 g, 52% yield). ESI-MS *m/z* 177.8 $[M+H]^+$; ^{1}H-NMR (400 MHz, $CDCl_3$) δ 7.05 – 6.97 (m, 1H), 6.95 – 6.87 (m, 3H), 4.25 (t, *J* = 6.7 Hz, 2H), 3.87 (s, 3H), 2.85 (t, *J* = 6.7 Hz, 2H).

A solution of the obtained 3-(2-methoxyphenoxy)propanenitrile (1.40 g, 7.90 mmol) in anhydrous THF (6 mL) was added dropwise a 1 M solution of borane tetrahydrofuran complex in THF (8.75 mL, 8.75 mmol). The reaction mixture was heated to 65°C for 15 h under argon atmosphere. After cooling to 0°C a 5 M NaOH solution (9.7 mL) was added slowly, and the reaction was heated up to 65°C again for further 4 h. After the reaction has cooled to room temperature ethyl acetate was added and the organic phase was washed with water and brine. After drying over Na_2SO_4 the organic solvent was evaporated in vacuo to give **11** as yellowish oil (874 mg, 61% yield). ESI-MS *m/z* 181.8 $[M+H]^+$; ^{1}H-NMR (400 MHz, $CDCl_3$) δ 6.95 – 6.83 (m, 4H), 4.11 (t, *J* = 6.2 Hz, 2H), 3.86 (s, 3H), 2.93 (t, *J* = 6.7 Hz, 2H), 2.02 – 1.93 (m, 2H), 1.65 (s, 2H); ^{13}C-NMR (151 MHz, $CDCl_3$) δ 149.47, 148.33, 121.25, 120.91, 113.38, 111.72, 67.58, 55.90, 39.59, 29.40.

N-(2-(2-(Methylthio)phenoxy)ethyl)acetamide [**12**]

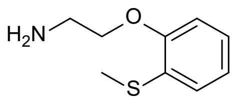

A mixture of 2-(methylthio)phenol (0.26 mL, 2.1 mmol) and 2-methyl-2-oxazoline (0.18 mL, 2.1 mmol) was stirred in a sealed tube at 160°C overnight under argon atmosphere. The crude product was purified by flash chromatography (isohexane/ ethyl acetate 3:1) to yield *N*-(2-(2-(methylthio)phenoxy)ethyl)acetamide as yellow oil (0.29 g, 61% yield). ESI-MS *m/z* 225.8 $[M+H]^+$; ^{1}H-NMR (400 MHz, DMSO-d_6) δ 8.04 (s, 1H), 7.18 – 7.08 (m, 2H), 7.00 – 6.94 (m, 2H), 4.01 (t, *J* = 6.0 Hz, 2H), 3.40 (q, *J* = 5.9 Hz, 2H), 2.37 (s, 3H), 1.83 (s, 3H).
A solution of the obtained *N*-(2-(2-(methylthio)phenoxy)ethyl)acetamide (200 mg, 887 µmol) in MeOH (5.0 mL) was added carefully HCl_{conc} (5.0 mL). The resulting mixture was refluxed for 24 h. After the solution had cooled to room temperature 5 M NaOH solution was added to adjust the pH to 9 - 10 and the resulting aqueous phase was extracted with ethyl acetate. The organic layers were combined, dried over Na_2SO_4 and evaporated in vacuo to yield **12** as yellow oil (154 mg, 95% yield). ESI-MS *m/z* 183.9 $[M+H]^+$; ^{1}H-NMR (400 MHz, DMSO-*d6*) δ 7.16 – 7.08 (m, 2H), 7.00 – 6.91 (m, 2H), 3.97 (t, J = 5.6 Hz, 2H), 2.90 – 2.85 (m, 2H), 2.37 (s, 3H); ^{13}C-NMR (101 MHz, DMSO-*d6*) δ 155.21, 127.57, 125.86, 125.35, 121.68, 112.11, 71.35, 41.40, 13.87.

6-(3-((3-(2-Methoxyphenoxy)propyl)amino)propoxy)-2H-benzo[b][1,4]oxazin-3(4H)-one [**TA12**]

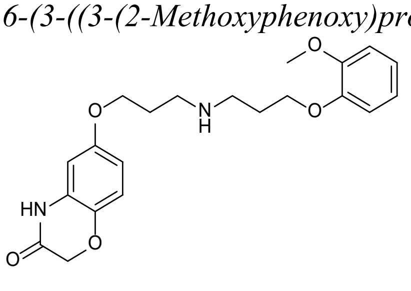

A suspension of **9** (15 mg, 52 µmol), **11** (24 mg, 0.13 mmol) and KI (13 mg, 79 µmol) in acetonitrile (3.3 mL) was heated in a sealed tube to reflux for 5 h under argon atmosphere. After the reaction had cooled to room temperature water was added and the aqueous phase was extracted with CH_2Cl_2. The combined organic layers were washed with brine, dried over Na_2SO_4 and evaporated under vacuo. The crude product was purified by preparative HPLC (10 - 90% CH_3CN/ 0.1% aq. trifluoroacetic acid) to give **TA12** as white TFA salt (15 mg, 57% yield). ESI-MS *m/z* 387.2 $[M+H]^+$; HR-MS calculated: 387.1914 $[M+H]^+$, found: 387.1912 $[M+H]^+$; ^{1}H-NMR (600 MHz, CDCl3) δ 9.34 (s, 2H), 8.88 (s, 1H), 7.01 – 6.96 (m, 1H), 6.92 – 6.85 (m, 3H), 6.77 (d, J = 8.8 Hz, 1H), 6.44 (d, J = 2.7 Hz, 1H), 6.40 (dd, J = 8.8, 2.8 Hz, 1H), 4.35 (s, 2H), 4.17 (t, J = 5.5 Hz, 2H), 4.06 (t, J = 5.6 Hz, 2H), 3.84 (s, 3H), 3.41 – 3.38 (m, 2H), 3.35 – 3.33 (m, 2H), 2.34 – 2.25 (m, 4H); ^{13}C-NMR (151 MHz, CDCl3) δ 165.96, 153.72, 149.34, 147.20, 137.81, 126.92, 122.77, 121.18, 116.99, 114.56, 111.76, 109.51, 102.84, 69.00, 67.17, 65.31, 55.83, 47.53, 45.42, 26.05, 24.98; HPLC system 1: t_R = 15.3 min, purity: 97%; system 2: t_R = 14.3 min, purity: 98%.

6-(4-((2-(2-Methoxyphenoxy)ethyl)amino)butoxy)-2H-benzo[b][1,4]oxazin-3(4H)-one [**TA13**].

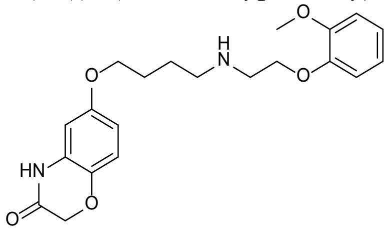

Compound **TA13** was prepared according to the protocol of **TA12** by refluxing a suspension of **10** (15 mg, 50 µmol), 2-(2-methoxyphenoxy)ethylamine (21 mg, 0.12 mmol) (*90*), and KI (12 mg, 75 µmol) in acetonitrile (3.3 mL) under argon atmosphere for 2.5 h. After cooling to room temperature water was added and the aqueous phase was extracted with CH_2Cl_2. The combined organic layers were washed with brine, dried over Na_2SO_4 and evaporated. Purification of the crude product by preparative HPLC (10 - 90% CH_3CN/ 0.1% aq. trifluoroacetic acid) yielded **TA13** as colorless TFA salt (13 mg, 52% yield). ESI-MS *m/z* 387.2 $[M+H]^+$; HR-MS calculated: 387.1915 $[M+H]^+$, found: 387.1917 $[M+H]^+$; ^{1}H-NMR (600 MHz, DMSO-*d6*) δ 10.65 (s, 1H), 8.82 (s, 2H), 7.05 – 7.01 (m, 2H), 7.00 – 6.97 (m, 1H), 6.92 – 6.89 (m, 1H), 6.88 – 6.84 (m, 1H), 6.50 – 6.48 (m, 2H), 4.48 (s, 2H), 4.22 (t, J = 5.2 Hz, 2H), 3.91 (t, J = 5.8 Hz, 2H), 3.78 (s, 3H), 3.37 – 3.32 (m, 2H), 3.14 – 3.07 (m, 2H), 1.88 – 1.71 (m, 4H); ^{13}C-NMR (151 MHz, DMSO-*d6*) δ 165.68, 154.23, 149.89, 147.51, 137.66, 128.49, 122.75, 121.15, 116.95, 115.40, 112.81, 108.51, 102.92, 67.61, 67.29, 65.41, 55.90, 47.47, 46.34, 26.24, 22.80; HPLC system 1: t_R = 16.1 min, purity: 98%; system 2: t_R = 15.2 min, purity: 99%.

6-(2-((2-(2-Methoxyphenoxy)ethyl)amino)ethoxy)-2H-benzo[b][1,4]oxazin-3(4H)-one [**TA14**]

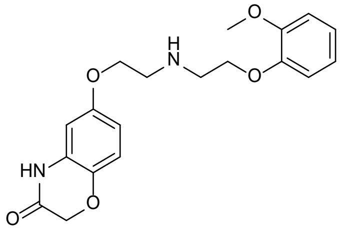

Compound **TA14** was prepared as described for **TA12**, by stirring a suspension of **8** (39 mg, 0.14 mmol), 2-(2-methoxyphenoxy)ethaylamine (60 mg, 0.36 mmol) (*90*), and KI (36 mg, 0.21 mmol) in acetonitrile (3.3 mL) in a sealed tube under argon atmosphere and reflux conditions for 2.5 h. After the reaction has cooled to room temperature water was added and the resulting aqueous phase was extracted with CH_2Cl_2. The combined organic layers were washed with brine, dried over Na_2SO_4 and evaporated in vacuo. The crude product was purified by preparative HPLC (10 - 90% CH_3CN/ 0.1% aq. trifluoroacetic acid) to give **TA14** as white TFA salt (32 mg, 48% yield). ESI-MS *m/z* 359.1 $[M+H]^+$; HR-MS calculated: 359.1602 $[M+H]^+$, found: 359.1599 $[M+H]^+$; ^{1}H-NMR (400 MHz, DMSO-*d6*) δ 10.75 (s, 1H), 8.86 (s, 2H), 7.07 – 7.03 (m, 1H), 7.02 – 6.97 (m, 2H), 6.93 – 6.92 (m, 1H), 6.91 – 6.88 (m, 1H), 6.60 – 6.53 (m, 2H), 4.51 (s, 2H), 4.25 (t, J = 5.1 Hz, 2H), 4.19 (t, J = 5.0 Hz, 2H), 3.78 (s, 3H), 3.48 (t, J = 4.9 Hz, 2H), 3.43 (t, J = 5.1 Hz, 2H); ^{13}C-NMR (101 MHz, DMSO-*d6*) δ 165.28, 153.03, 149.43, 147.02, 137.74, 128.11, 122.37, 120.72, 116.60, 114.96, 112.32, 108.12, 102.89, 66.82, 64.94, 63.80, 55.46, 46.51, 46.31; HPLC system 1: t_R = 15.3 min, purity: 98%; system 2: t_R = 14.4 min, purity: 97%.

6-(3-((2-(2-(Methylthio)phenoxy)ethyl)amino)propoxy)-2H-benzo[b][1,4]oxazin-3(4H)-one [**TA48**]

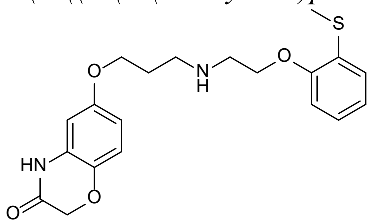

Compound **TA48** was prepared as described for **TA13** by refluxing a mixture of **9** (70 mg, 0.32 mmol), **12** (0.14 g, 0.79 mmol) and KI (78 mg, 0.47 mmol) in acetonitrile (3.8 mL) under argon atmosphere for 4 h. After cooling to room temperature water was added and the aqueous phase was extracted with ethyl acetate. The organic layers were combined, washed with brine, dried over $MgSO_4$ and evaporated in vacuo. The crude product was purified by preparative HPLC (10 - 90% CH_3CN/ 0.1% aq. trifluoroacetic acid) to give **TA48** as white TFA salt (62 mg, 39% yield). ESI-MS *m/z* 389.0 $[M+H]^+$; HR-MS calculated: 389.1530 $[M+H]^+$, found: 389.1531 $[M+H]^+$; ^{1}H-NMR (600 MHz, DMSO-*d6*) δ 10.68 (s, 1H), 8.72 (s, 2H), 7.21 – 7.15 (m, 2H), 7.07 – 7.03 (m, 2H), 6.88 (d, J = 8.7 Hz, 1H), 6.50 (dd, J = 8.7, 2.9 Hz, 1H), 6.48 (d, J = 2.8 Hz, 1H), 4.50 (s, 2H), 4.30 (t, J = 5.1 Hz, 2H), 3.99 (t, J = 5.9 Hz, 2H), 3.46 – 3.41 (m, 2H), 3.31 – 3.25 (m, 2H), 2.38 (s, 3H), 2.11 – 2.06 (m, 2H); ^{13}C-NMR (151 MHz, DMSO-*d6*) δ 165.12, 153.74, 153.41, 137.22, 127.89, 127.28, 125.32, 125.05, 122.14, 116.38, 112.10, 107.90, 102.36, 66.69, 64.94, 64.62, 45.85, 45.16, 25.54, 13.34; HPLC system 1: t_R= 15.8 min, purity: 98%; system 2: t_R= 14.6 min, purity: 99%.

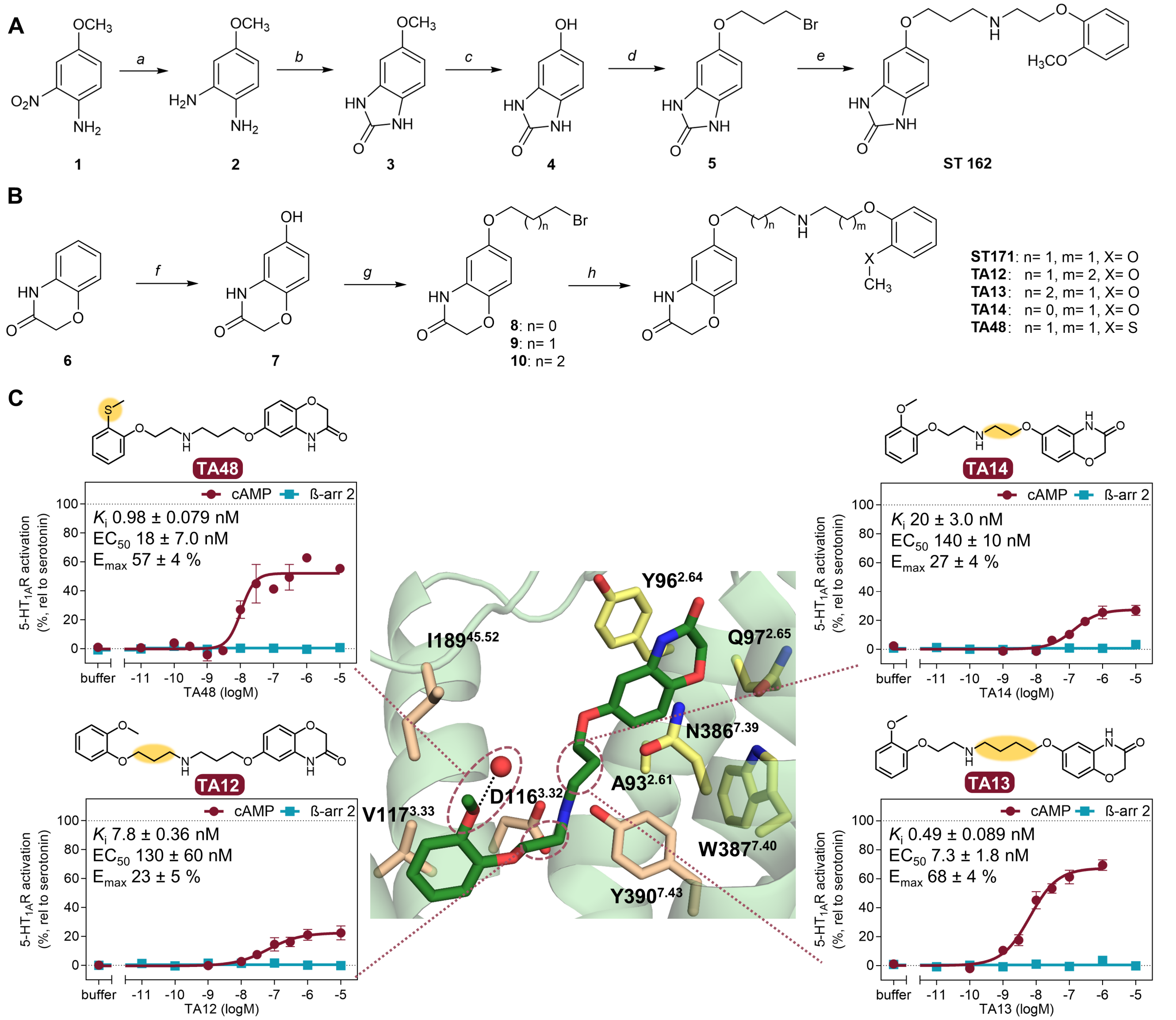

Fig. S1. Chemical synthesis of ST162 and ST171 and structure activity relationships of analogs thereof.

(**A**) Synthesis of **ST162**, reagents and conditions: (a) H_2, Pd/C, methanol, rt, 24 h (78%); (b) 1,1'-carbonyldiimidazole, DMF, Ar, 90°C, 21 h, (68%); (c) 1 M BBr_3 in CH_2Cl_2, CH_2Cl_2, Ar, -78°C-rt, 3h (59%); (d) 1,3-dibromopropane, K_2CO_3, ethanol, reflux, 3.5 h (30%); (e) 2-methoxyphenoxyethylamine (*90*), KI, CH_3CN, reflux, 4.5 h (31%). (**B**) Chemical synthesis of **ST171** and its analogs **TA12**, **TA13**, **TA14** and **TA48**, feagents and conditions: f) [bis(trifluoroacetoxy)iodo] benzene, trifluoroacetic acid, reflux, 10 min (45%); (g) 1,3-dibromoalkane, K_2CO_3, ethanol, reflux, 3.5 h (53-63%); (h) 2-methoxyphenoxyethylamine (*90*), 3-(2-methoxyphenoxy)propylamine [**11**], or 2-(2-methylthiophenoxy)ethylamine [**12**], KI, CH_3CN, reflux, 2.5-5 h (39-74%). (**C**) SAR study with different modifications of the ST171 scaffold, with affinities (K_i) determined in displacement assays with 5-HT_{1A}R and [^{3}H]WAY600,135, inhibition of cAMP accumulation determined with the CAMYEL biosensor, and recruitment of β-arrestin 2 measured with the Pathhunter assay. Substitution of ST171's ether moiety with a thioether in **TA48** resulted in a decrease in both potency and efficacy that can be attributed to the relatively diminished hydrogen-bond accepting capacity of the thioether. This observation suggests the potential involvement of the methoxy group in ligand binding, particularly in the formation of a water-

mediated hydrogen bond. Modifying the positioning of the benzoxazinone moiety through linker unit shortening (**TA14**) or extension (**TA13**) leads to a substantial reduction in both potency and efficacy, thereby affirming the essential role of the benzoxazinone moiety in ligand binding. Insertion of a CH_2 unit between the head group and the secondary amine (**TA12**) leads to reduced binding affinity and receptor activation relative to ST171, highlighting the importance of the salt bridge to D116$^{3.32}$. None of the tested compounds showed recruitment of β-arrestin 2 (PathHunter assay, E_{max} < 5%, n = 3). Data show mean ± SEM of *n* = 3 (binding, arrestin recruitment) or *n* = 4 (cAMP) individual experiments.

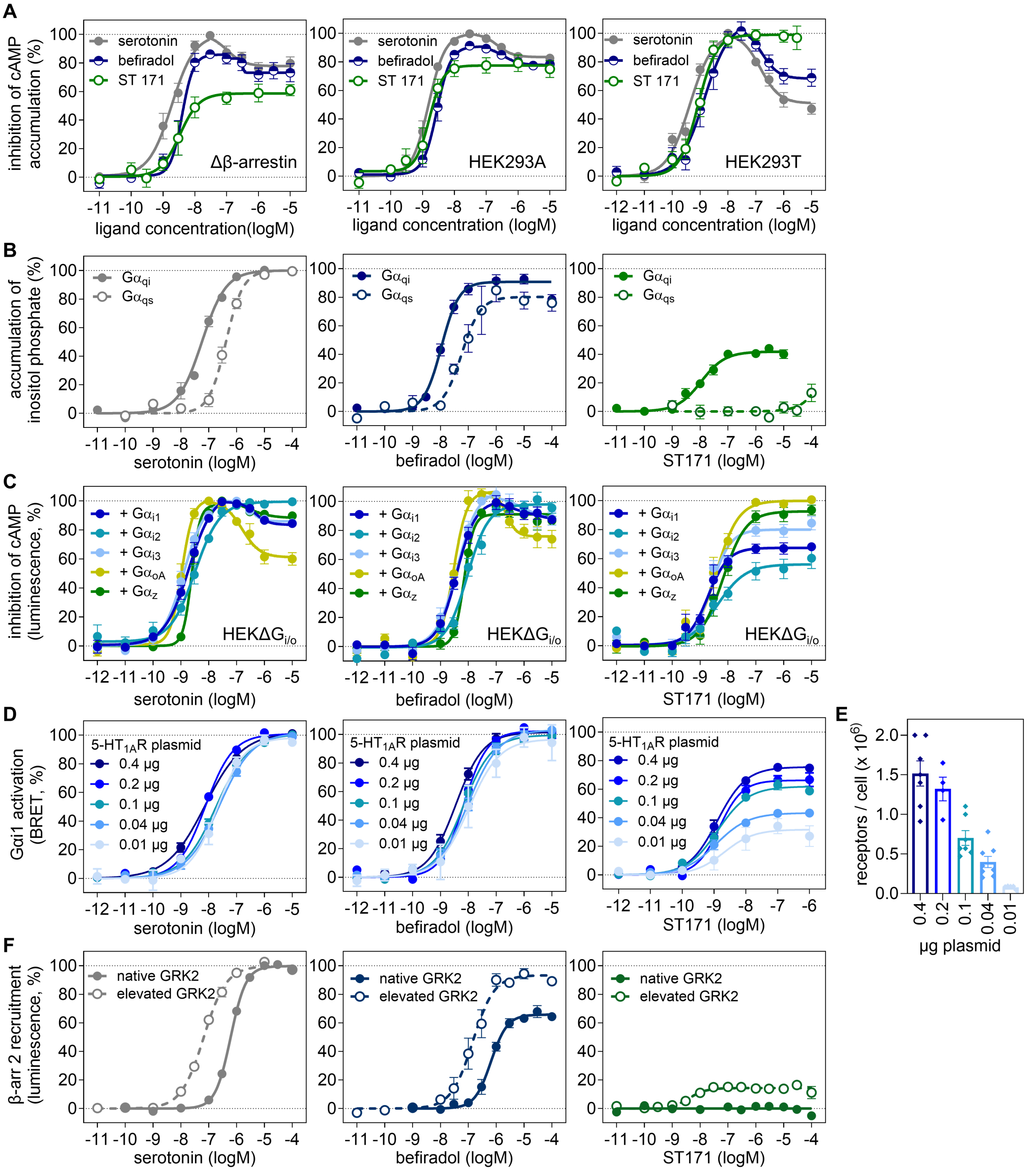

Fig. S2. Extended 5-HT$_{1A}$R activation profiles of serotonin, befiradol, and ST171 in different assays and cell lines.

(**A**) Inhibition of cAMP accumulation in HEK293A cells deficient of β-arrestins, HEK293A cells, and HEK293T cells recorded with the GloSensor (*n* = 5-7, as triplicates (duplicates for HEK293T)). (**B**) Gα_i and Gα_s signaling determined in an inositol phosphate accumulation assay with hybrid Gα_{qi} and Gα_{qs} proteins (*n* = 6-8, in duplicates) indicates activation of $G_{i/o}$ and G_s by serotonin and befiradol (serotonin: $EC_{50}[G_{qi}]$ = 58 ± 9.4 nM, $EC_{50}[G_{qs}]$ = 420 ± 70 nM; befiradol: $EC_{50}[G_{qi}]$ = 12 ± 1.3 nM, $E_{max}[G_{qi}]$ = 92

± 3%; $EC_{50}[G_{qs}]$ = 53 ± 3.7 nM, $E_{max}[G_{qs}]$ = 83 ± 6%), but only activation of Gi for ST171 ($EC_{50}[G_{qi}]$ = 13 ± 2.9 nM, $E_{max}[G_{qi}]$ = 43 ± 2%). (**C**) Inhibition of cAMP accumulation by serotonin, befiradol, and ST171 in HEK293AΔ$G_{i/o}$ cells is rescued by co-transfection with $G\alpha_{i1}$, $G\alpha_{i2}$, $G\alpha_{i3}$, $G\alpha_{z}$, or $G\alpha_{oA}$. Data was obtained in 5 independent experiments with the GloSensor in triplicates. (**D**) Activation of a $G\alpha_{i1}$ BRET biosensor in HEK293T cells expressing different amounts of 5-HT_{1A}R according to the transfection with 0.01-0.4 µg of 5-HT_{1A}R plasmid/culture plate, (*n* = 5-17, as duplicates). (**E**) 5-HT_{1A}R densities determined in radioligand saturation experiments with [^{3}H]WAY100,635 in parallel to the BRET $G\alpha_{i1}$ activation assay. Data (mean ± SEM and individual data points) were obtained in *n* = 4-8 independent experiments each performed in triplicates. (**F**) *β*-arrestin 2 recruitment with native and elevated GRK2 levels was determined in the sensitive PathHunter assay (*n* = 7-12, as duplicates). The 9-fold (serotonin) and 4-fold (befiradol) increase of potency, respectively, and an approximately 30% increase in efficacy for befiradol underline the relevance of GRK2 (serotonin, native GRK2 EC_{50} = 620 ± 35 nM, elevated GRK2 EC_{50} 67 ± 4.8 nM; befiradol native GRK2 EC_{50} = 690 ± 120 nM, E_{max} = 67 ± 3%, elevated GRK2 EC_{50} = 180 ± 47 nM, E_{max} = 96 ± 3%). The functional selectivity of ST171 for $G_{i/o}$ protein activation over β-arrestin 2 recruitment is confirmed by the weak partial agonist effect only observed in the presence of elevated GRK2 levels (EC_{50} = 5.5 ± 1.4 nM, E_{max} = 15 ± 1%). (A-D, F) All data was normalized to the maximum response of serotonin and is indicated as mean ± SEM of *n* independent experiments.

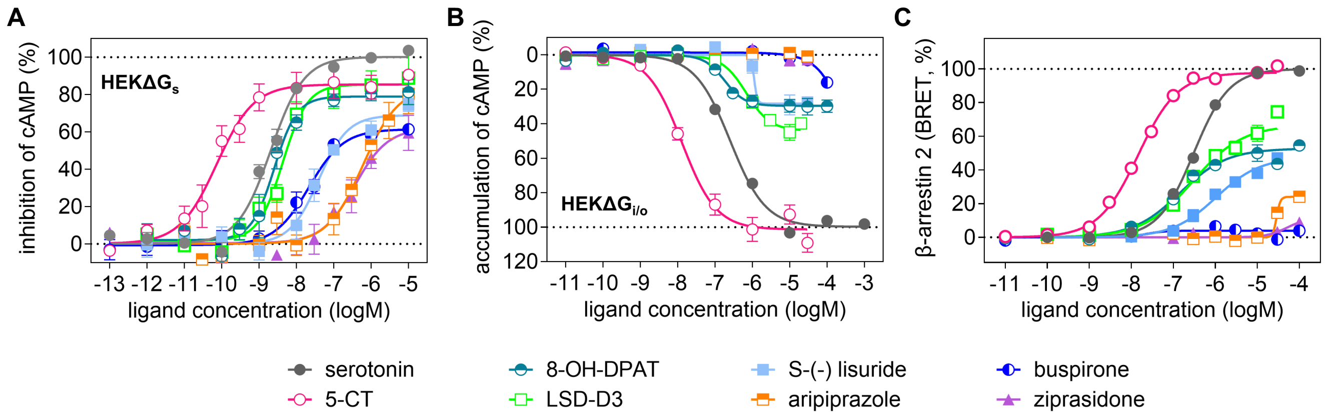

Fig. S3. Signaling profile of selected reference ligands at 5-HT$_{1A}$R.

(**A**) 5-HT$_{1A}$R mediated inhibition of cAMP accumulation in HEK293A cells deficient of Gα_s subunits was measured with the GloSensor (n = 5-8, in triplicates), (**B**) accumulation of cAMP in HEK293A cells deficient of G$\alpha_{i/o}$ subunits was recorded with the GloSensor (n=5-7, in triplicates). (**C**) Recruitment of β-arrestin 2 labeled with RlucII was assessed in the presence of GRK2 by enhanced bystander BRET to membrane-anchored GFP (CAAX-rGFP, n = 3-6, in duplicates). (A-C) All data was normalized to the maximum response of serotonin and is indicated as mean ± SEM.

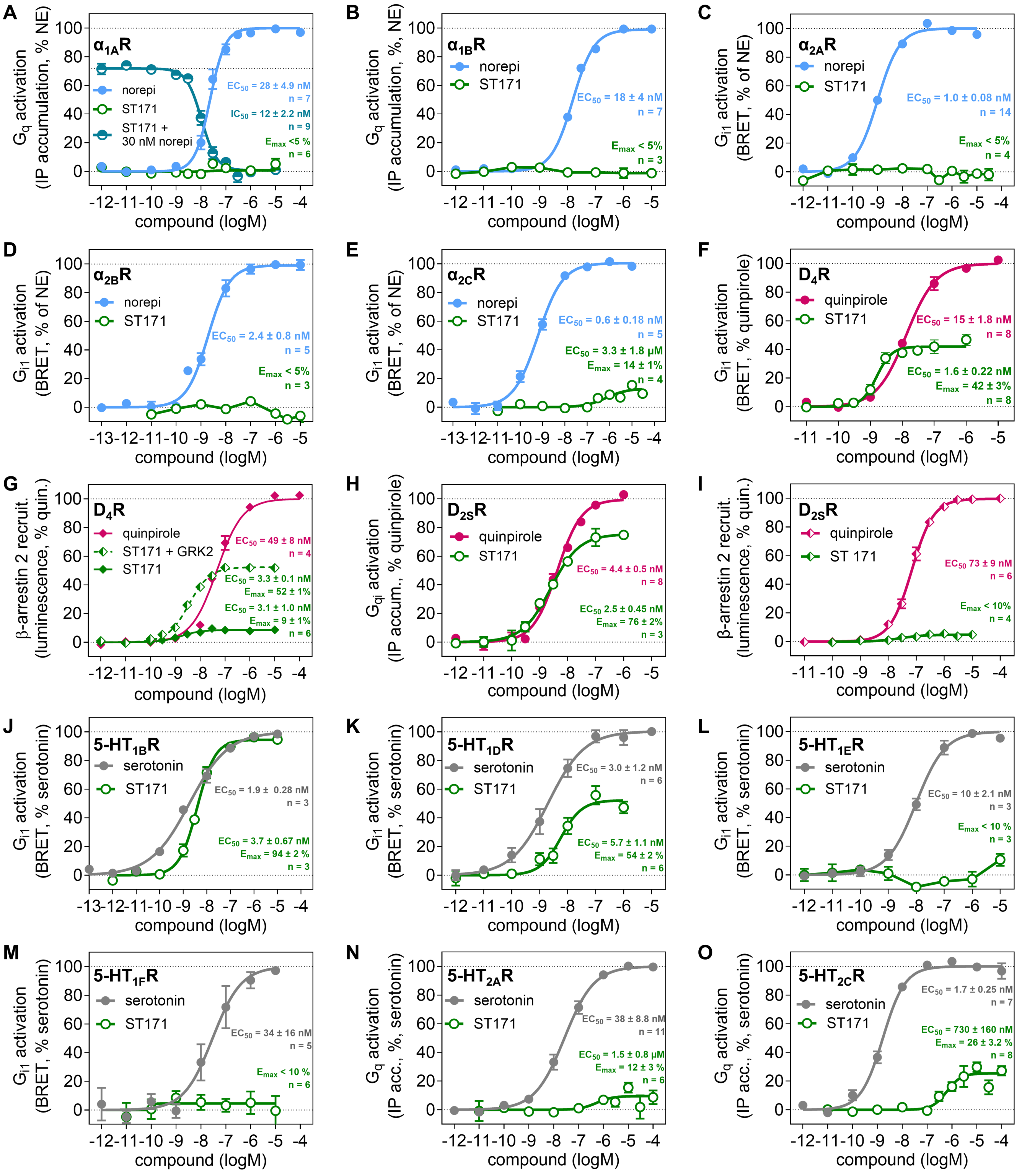

Fig. S4. ST171 activity profile for selected aminergic GPCRs.

(**A**, **B**) In IP accumulation assays with α_{1A}R or α_{1B}R, ST171 is devoid of agonistic activity. ST171's antagonism at α_{1A}R is confirmed in co-incubation experiments with 30 nM norepinephrine. (**C**-**E**) Gα_{i1} activation by α_{2A}R, α_{2B}R, and α_{2C}R was assessed by BRET (Gα_{i1}-RlucII, GβGγ-GFP10 sensor). (**F**, **G**) Gα_{i1} activation and β-arrestin 2 recruitment by D4R was assessed by BRET (Gα_{i1}-RlucII, GβGγ-GFP10) and the enzyme fragment complementation assay (Pathhunter) in the absence and presence of GRK2,

respectively. The experiments revealed partial agonist activity of ST171 at D_4R. (**H**, **I**) ST171 is a partial agonist for G protein activation at $D_{2S}R$ (measured by an IP accumulation assay with a hybrid G_{qi} protein) and has little activity in the enzyme fragment complementation-based β-arrestin 2 recruitment assay. (**J-M**) ST171's activity at other $5\text{-}HT_1R$ subtypes was measured by BRET ($G\alpha_{i1}$-RlucII, GβGγ-GFP10 sensor). (**N**, **O**) ST171-mediated activation of the $G\alpha_q$-coupled $5\text{-}HT_{2A}R$ and $5\text{-}HT_{2C}R$ subtypes was measured in IP accumulation assays in HEK293T cells and revealed low potency partial agonist activity. (A-O) All data are represented as mean ± SEM of 3-14 independent experiments, with the individual number *n* indicated in the subpanels.

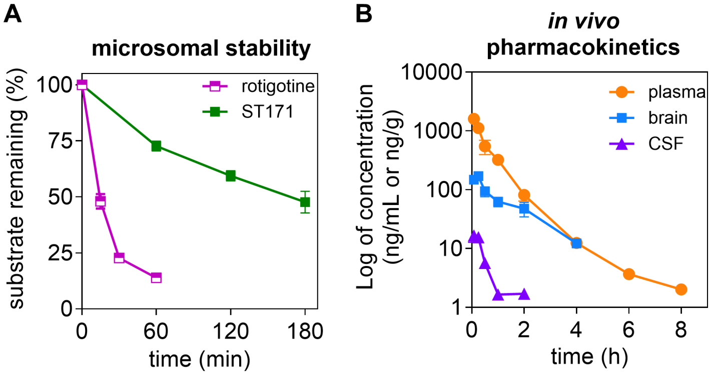

C

matrix	T_{max} * (h)	C_{max} ($ng \cdot mL^{-1}$)	AUC_{last} # ($h \cdot ng \cdot mL^{-1}$)	$T_{1/2}$ § (h)
plasma	0.08	1604	1037	0.9
brain	0.25	170	220	–
CSF	0.08	16.7	8.57	–

* Time of maximum concentration;
# area under the curve of concentration versus time;
§ half-life.

Fig. S5. Metabolic stability and pharmacokinetic properties of ST171.

(**A**) Metabolic stability of ST171 was assessed *in vitro* with male rat liver microsomes. While the reference compound rotigotine (*91*) is rapidly metabolized (< 15% intact substrate remaining after 1 h), ST171 shows decent metabolic stability (> 70% intact substrate after 1 h, > 45% after 3 h incubation). Similar to the reported metabolism of the FDA-approved α/β-blocker carvedilol (*92*), which shares the 2-methoxyphenoxyethylamine moiety with ST171, HPLC-MS analysis of the reaction mixtures revealed hydroxylation and demethylation as the most likely biotransformation reactions. (**B**, **C**) Graphic and tabular representation of *in vivo* pharmacokinetic studies with ST171 in male C57BL/6 mice. After i.p. administration of a 10 mg/kg dose, ST171 rapidly penetrates into the brain. ST171 is also detected in the CSF (C_{max} ~40 nM), serving as an approximate for the free drug concentration within the brain (*93*). (A, B) Data show mean ± SEM of three independent experiments.

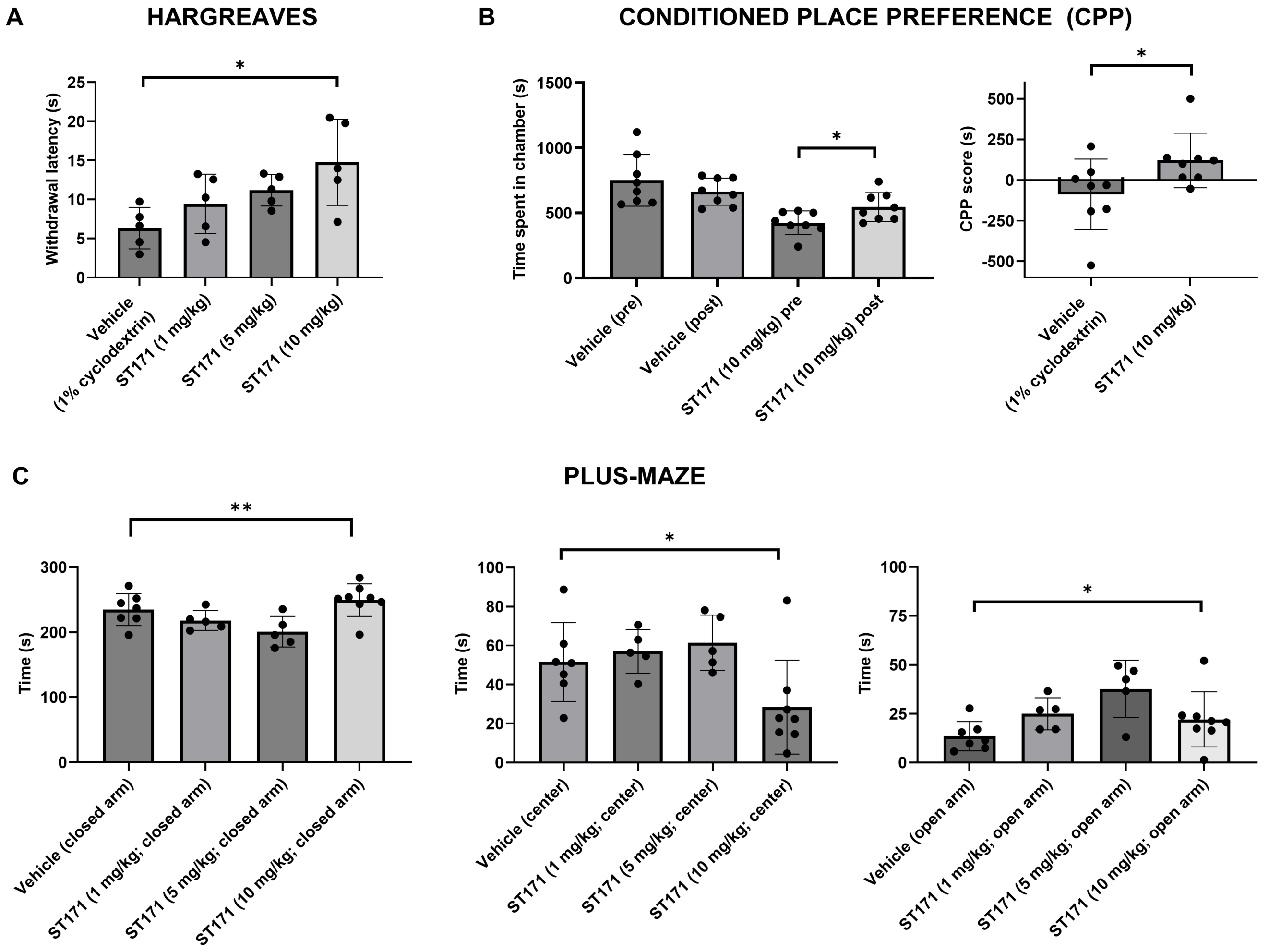

Fig. S6. Analgesic doses of ST171 are rewarding and anxiolytic.

(**A**) Dose response for ST171-induced analgesia in the Hargreaves test. One-way ANOVA with Dunnett's post-hoc test, *$p<0.05$. (**B**) In naïve mice, the 10 mg/kg analgesic dose of ST171 is rewarding in the CPP assay; mice spent more time in the chamber associated with the compound than with the vehicle control. Unpaired Student's t-test; *$p<0.05$. (**C**) In the Plus Maze assay, ST171 exerted anxiolytic effects that were dose-dependent; mice injected with 1 or 5 mg/kg of ST171 spent more time in the center and open arms of the apparatus and correspondingly less time in the closed arms. One-way ANOVA with Dunnett's post-hoc test, *$p<0.05$, **$p<0.01$.

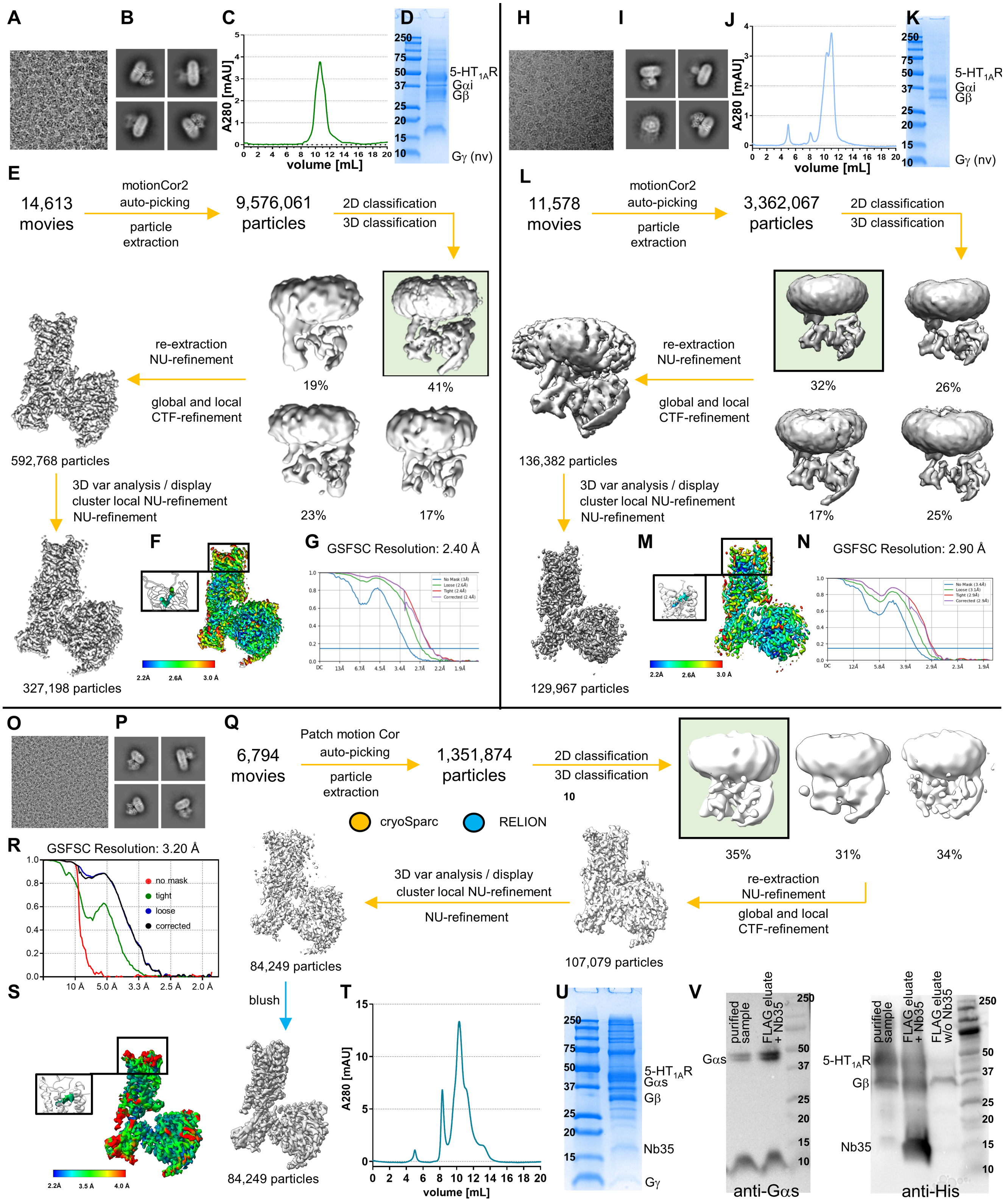

Fig. S7. Data processing and complex analytics for the cryo-EM structures of 5-HT$_{1A}$R. (**A-G**) ST171 / 5-HT$_{1A}$R-G$_{i1}$, (**H-N**) befiradol / 5-HT$_{1A}$R-G$_{i1}$, (**O-T**) befiradol / 5-HT$_{1A}$R-G$_s$ complex. (**A**, **H**, **O**) Motion corrected and dose weighted average of movies. (**B**, **I**, **P**) Examples of selected class averages. (**C**, **J**, **T**) Size-exclusion chromatography profiles of the concentrated samples used for cryo-EM. (**D**, **K**, **U**) Coomassie-stained SDS-PAGE analysis of the receptor complexes. (**E**, **L**, **Q**) Cryo-EM

single particle analysis workflow, the green subsets were selected for the subsequent steps. (**F**, **M**, **S**) Maps filtered and colored according to local resolution with the local resolution of the ligands shown as close up. (**G**, **N**, **R**) FSC curves of the 3D reconstructions. (**V**) Western blot with rabbit anti-$G\alpha_S$ and mouse anti-His specific antibodies at different stages of the purification shows Flag-10xHis-5-$HT_{1A}R$, $G\alpha_s$ (untagged), 8xHis-Gβ, and Nb35-6xHis. Lanes are labeled as 'purified sample' – complex purified by SEC (5-$HT_{1A}R$, $G\alpha_S G\beta G\gamma$, Nb35), suitable for cryo-EM; 'FLAG eluate + Nb35' – concentrated elution fraction from FLAG resin complexed with separately purified Nb35-6xHis; 'FLAG eluate w/o Nb35' – for reference: elution fractions from a FLAG resin purification of the complex (5-$HT_{1A}R$ + $G\alpha_S G\beta G\gamma$) without addition of Nb35.

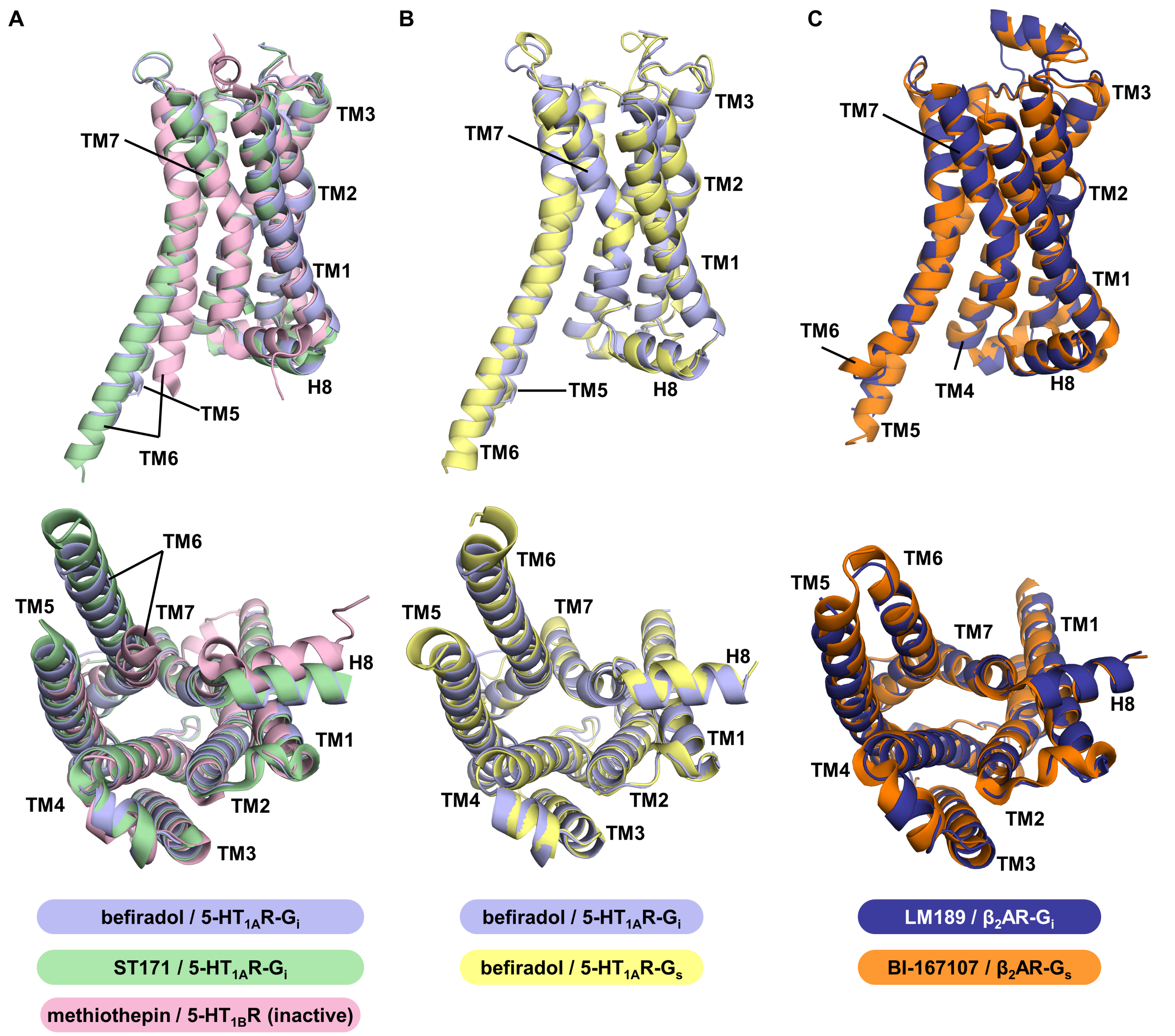

Fig. S8. Comparison of the 5-HT$_{1A}$R structures with inactive state 5-HT$_{1B}$R and β_2-AR coupled to G$_i$ and G$_s$ proteins.

(**A**) The overlay of the ST171- and befiradol-bound 5-HT$_{1A}$R protein structures obtained in complex with the G$_{i1}$ protein and the inactive state of 5-HT$_{1B}$R bound to the inverse agonist methiothepin (pdb 5V54) (*61*) viewed from the side and the intracellular surface reveals the typical active-state like outward-rotation of TM6 for the two G$_i$-bound complexes. G proteins and ligands were omitted for clarity. (**B**) Comparison of befiradol-bound 5-HT$_{1A}$R structures obtained with the G$_{i1}$ or the G$_s$ protein shows the high similarity between the two complexes. (**C**) Overlay of the LM189-bound cryo-EM structure of the β_2-AR obtained in complex with its secondary coupling partner G$_i$ (pdb 9BUY) (*62*) and the X-ray structure of the β_2-AR obtained with the full agonist BI-167107 in complex with the preferred coupling partner G$_s$ (pdb 3SN6) (*94*) also reveals high similarity of the two receptor structures. Ligands and G proteins were omitted for clarity.

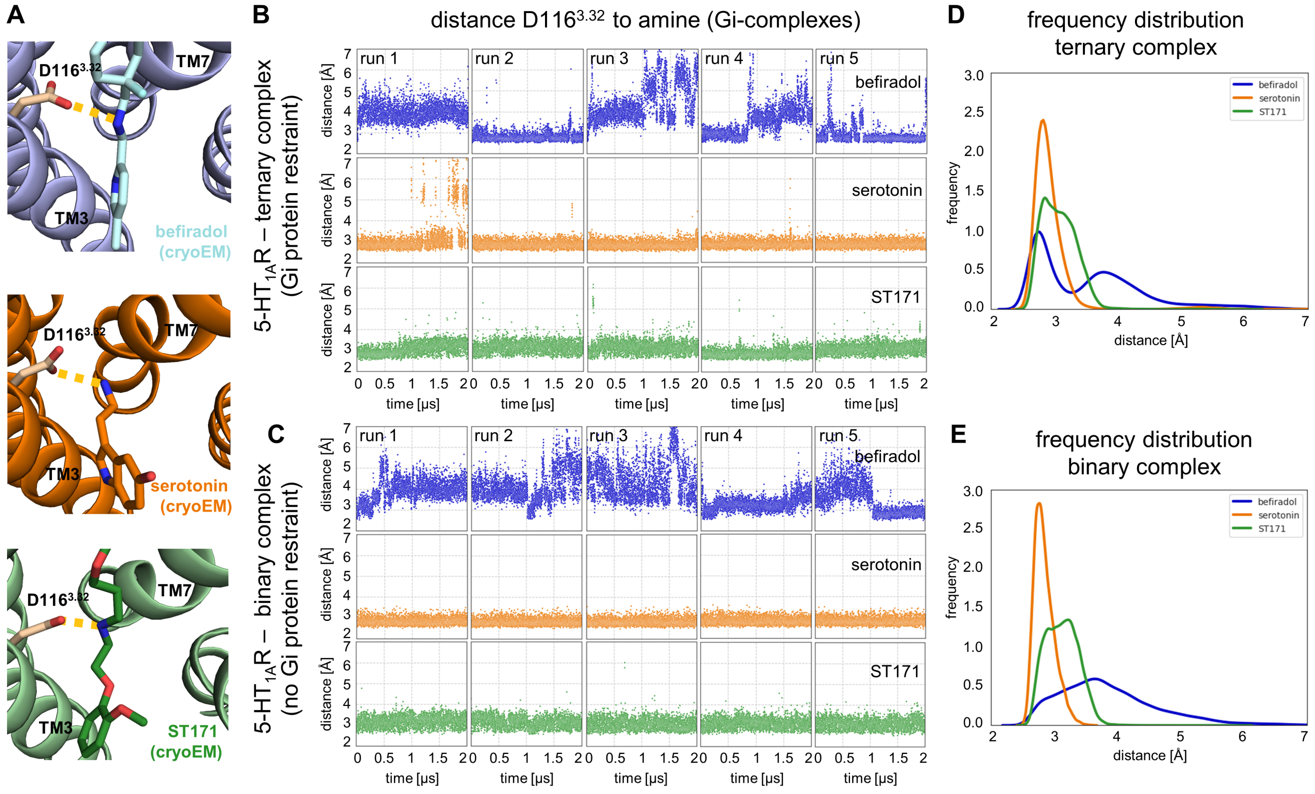

Fig. S9. Stability analysis of the binding poses regarding the orthosteric interaction with D116$^{3.32}$. (**A**) Distance between D116$^{3.32}$ (nearest oxygen atom of the carboxy function) and the nitrogen of the basic amine of the ligands in the experimental 5-HT$_{1A}$R-G$_i$ cryo-EM structures for befiradol (pdb 8PKM), serotonin (pdb 7E2Y), and ST171 (pdb 8PJK). (**B**, **C**) Progression of the distance between D116$^{3.32}$ and the basic amine of the respective ligand for 5 x 2 µs simulations of the ligand-bound 5-HT$_{1A}$R (**B**) in ternary complexes (simulation restraint on the G$_i$ protein interface) and (**C**) binary 5-HT$_{1A}$R complexes and (**D**, **E**) probability distribution based on the combined trajectory frames. A Gaussian kernel density estimation was used. The distance was determined between the center of mass of the carboxy function of D116$^{3.32}$ and the nitrogen atom of the basic amine of the ligand. The plots (B, C) depict the transition of the distance from low to high values in the befiradol-bound ternary complex compared to the respective binary complex, suggesting a dynamic behavior of befiradol within the binding pocket.

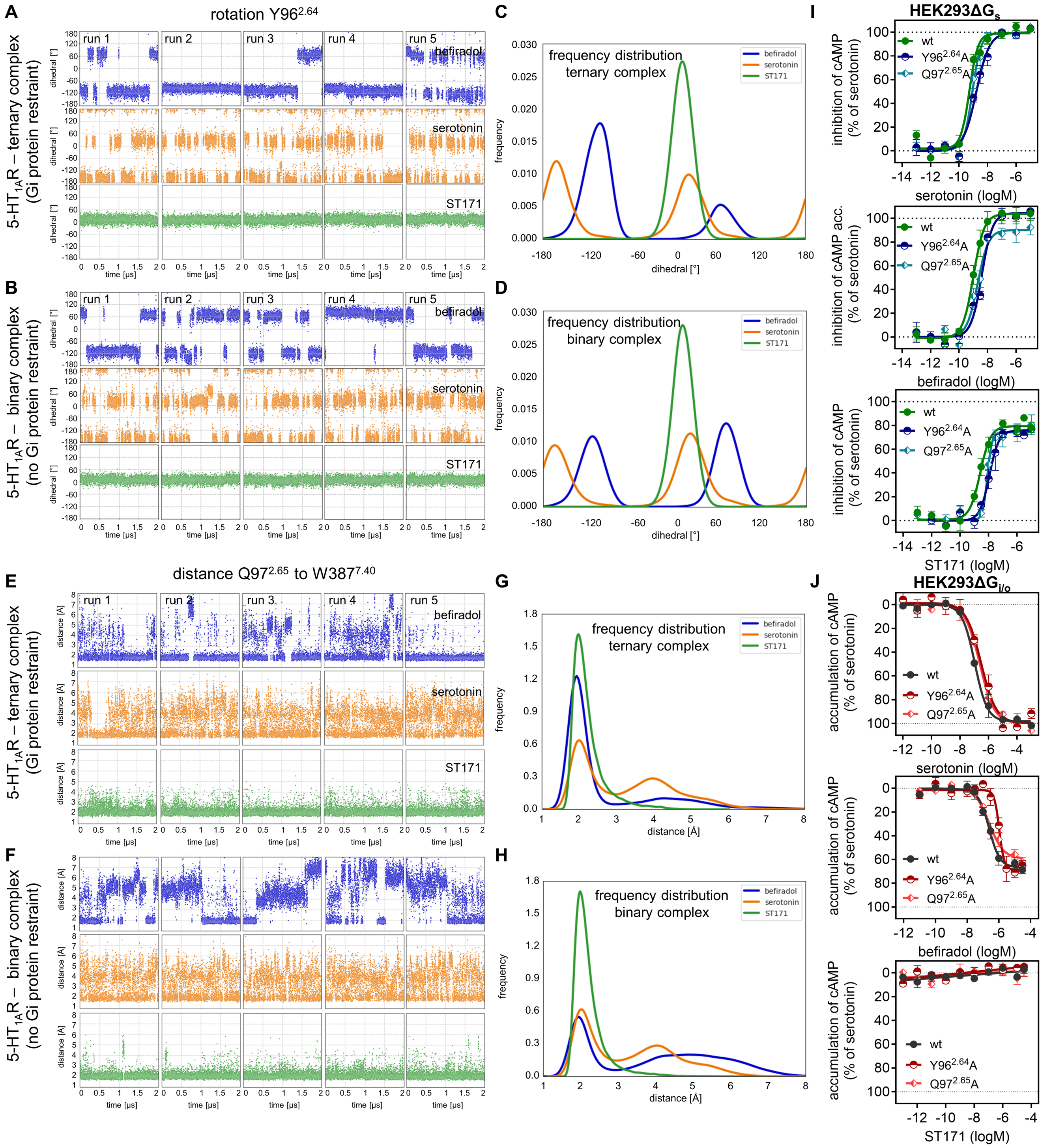

Fig. S10. Befiradol and ST171 differentially stabilize the conformations of Y96$^{2.64}$, Q97$^{2.65}$, and W387$^{7.40}$.

(**A**,**B**) Progression of the torsion angle -Cα-Cβ-Cγ-Cδ- of the Tyr96$^{2.64}$ side chain over the course of 5 independent simulations of the respective ligand-bound 5-HT$_{1A}$R complexes (pdb befiradol: 8PKM; ST171: 8PJK; serotonin: 7E2Y) for (**A**) ternary complexes (simulation restraint on the G$_i$ protein interface), (**B**) binary complexes, and (**C**,**D**) resulting probability distributions based on the combined trajectory frames. The von Mises kernel was utilized as a weighted kernel density estimator. Due to the ambiguity of Cδ in tyrosine side chains, the Cδ that pointed towards or was closest to the extracellular

side was used for analysis. Following IUPAC recommendations (*95*), positive values indicate counterclockwise rotation of the bond between C_γ and C_δ relative to the C_α and C_β bond, while negative values indicate clockwise rotation. (**E**,**F**) The distance between the amide group of Q97$^{2.65}$ and NH W387$^{7.40}$ was monitored over the course of 5 independent simulations of the respective ligand-bound (**E**) ternary complex (restraint on the G_i protein interface) and (**F**) binary complex (without G_i protein restraint). (**G**,**H**) The probability distribution based on the combined trajectory frames from all simulations shows that shorter distances between Q97$^{2.65}$ and W387$^{7.40}$ in the befiradol-bound receptor are more likely if the receptor is in the ternary complex. (**I**) Inhibition of cAMP accumulation by serotonin, befiradol, and ST171 for 5-HT_{1A}R mutants Y96$^{2.64}$A and Q97$^{2.65}$A in comparison to the wildtype was measured with the cAMP GloSensor in HEK293ΔG_s cells. (**J**) Accumulation of cAMP by serotonin, befiradol, and ST171 at the 5-HT_{1A}R mutants Y96$^{2.64}$A and Q97$^{2.65}$A was measured in comparison to the wildtype with the cAMP GloSensor in HEK293Δ$G_{i/o}$ cells. (I, J) Data show mean ± SEM of n = 3 independent experiments, each performed in triplicates.

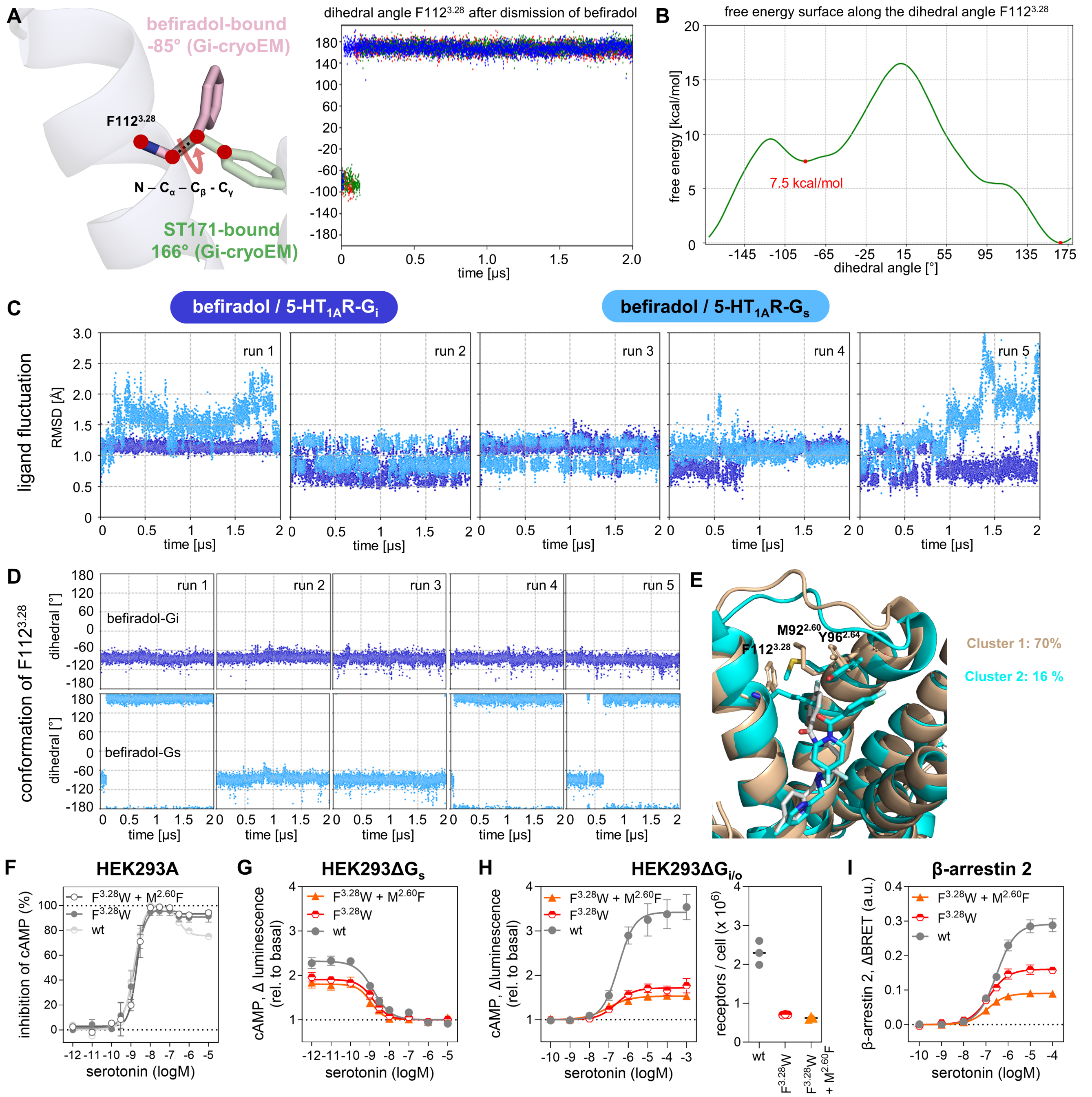

Fig. S11. Importance of the gatekeeper residue F112$^{3.28}$ in the extended binding pocket for befiradol.

(**A**) Comparison of the rotamers of F112$^{3.28}$ in the ST171-bound and the befiradol-bound cryo-EM structures of the 5-HT$_{1A}$R in complex with G$_i$, and measurement of the dihedral angle of F112$^{3.28}$ over 3 independent runs in an unbiased MD simulation (2 µs) of the befiradol-bound binary complex model after dismission of befiradol. (**B**) Free energy profile along the dihedral angle of F112$^{3.28}$ obtained by metadynamics simulations (6.7 µs) of the befiradol-bound binary complex model after dismission of befiradol. (**C**) The stability of the befiradol-conformation observed by cryo-EM of the 5-HT$_{1A}$R in complex with either the G$_i$ or G$_s$ protein is monitored in 5 x 2 µs MD simulations with the ternary complexes (restraint on the G protein interface). The simulations confirm the general stability of the distinct ligand conformations observed in the presence of the G$_i$ and G$_s$ protein, respectively, but suggest

higher variability in the G_s-like binding pose. (**D**) Analysis of the conformation of the gatekeeper residue F112$^{3.28}$ in the ternary complexes of 5-HT$_{1A}$R with befiradol and the G_i or G_s protein reveals high stability of the residue's conformation in the G_i-bound complex. In part a closure of the subpocket by rotation of F112$^{3.28}$ is observed for the G_s-bound complex, suggesting that the two G protein subtypes differentially stabilize the receptor-ligand complex. (**E**) A cluster analysis of befiradol's pose throughout the simulations of the ternary G_s-complex confirms the pose observed by cryo-EM but also reveals a second receptor/ligand conformation with distinct orientations of the ligand, F112$^{3.28}$, and Y96$^{2.64}$ that resemble the conformations observed in the presence of ST171. (**F**) Functional assessment of serotonin at the 5-HT$_{1A}$R wildtype compared to the F112$^{3.28}$W and the F112$^{3.28}$W + M92$^{2.60}$F mutants in the cAMP Glosensor assay in HEK293A cells reveals nearly identical potencies. The almost sigmoid concentration-response curves suggest limited coupling of the 5-HT$_{1A}$R mutants to G_s proteins. Data is shown as mean ± SEM for n = 3-8 independent experiments. (**G-I**) Although the extended binding pocket between TM2, TM3 and ECL1 is not addressed by serotonin, the residue substitutions F112$^{3.28}$W and M92$^{2.60}$F modestly influence (**G**) Gi protein activation (HEK293ΔG_s cells) and (**H**) substantially impair the receptor's capacity to signal via G_s proteins (HEK293Δ$G_{i/o}$ cells). Data were normalized to the basal luminescence of each experiment and are shown ± SEM of n = 3-6 independent experiments. (**H**)The mutations also negatively influence 5-HT$_{1A}$R expression as determined in radioligand binding experiments with [^{3}H]WAY100,635. Data are shown as mean and individual data points of three independent experiments. (**I**) The residue substitutions F112$^{3.28}$W and M92$^{2.60}$F reduce serotonin's ability to induce the recruitment of β-arrestin 2 as determined by bystander BRET in the presence of GRK2. Data is shown as ligand-induced change (ΔBRET) ± SEM of n = 3 independent experiments.

Table S1. Binding affinity for ST171, serotonin, and befiradol to the serotonin receptor subtypes 5-HT1AR (incl. mutants), 5-HT2AR, and 5-HT6R.

compound	K_i [nM ± SEM]* and number of experiments (*n*)[†]		
	ST171	serotonin	befiradol
5-HT1AR			
wt	0.41 ± 0.06 (*11*)	220 ± 52 (*8*)	15 ± 3.4 (*7*)
Y96$^{2.64}$A	63 ± 17 (*9*)	800 ± 200 (*8*)	200 ± 41 (*7*)
Q97$^{2.65}$A	63 ± 13 (*10*)	530 ± 110 (*7*)	64 ± 12 (*6*)
F112$^{3.28}$W	0.16 ± 0.05 (*4*)	140 ± 29 (*3*)	240 ± 63 (*5*)
F112$^{3.28}$W + M92$^{2.60}$F	0.044 ± 0.008 (*5*)	53 ± 8.6 (*5*)	330 ± 46 (*5*)
5-HT2AR	280 ± 30 (*10*)	130 ± 22 (*6*)	15,000 ± 3,700 (*4*)
5-HT6R	3300 ± 300 (*4*)	140 ± 37 (*7*)	>50,000 (*3*)

* Radioligand displacement experiments with membranes from transiently transfected HEK 293T cells.
[†] Number of individual experiments each done in triplicate.

Table S2. ST171, serotonin, and befiradol differentially modulate the intracellular cAMP concentration via wildtype and mutant 5-HT$_{1A}$R.

	cell system	compound	functional activity				
			EC_{50}^{left} [nM±SEM] ‡	E_{max} [%±SEM] #	EC_{50}^{right} [nM±SEM] ‖	E_{sat} [%±SEM]¶	n#
wildtype	CHOK1*	ST171	0.88 ± 0.30	87 ±1	- - -	- - -	3
		serotonin	3.6 ± 0.6 nM	100	240 ± 70	64 ± 3	4
		befiradol	1.8 ± 0.3	91 ± 3	110 ± 1	65 ± 4	5
	HEK Δβ-arrestin†	ST171	3.5 ± 1.0	60 ± 3	- - -	- - -	7
		serotonin	1.9 ± 0.2	100	140 ± 44	77 ± 3	7
		befiradol	4.7 ± 0.9	89 ± 4	330 ± 120	73 ± 5	7
	HEK293A†	ST171	3.4 ± 0.8	86 ± 5	- - -	- - -	12
		serotonin	1.5 ± 0.2	100	350 ± 40	78 ± 2	15
		befiradol	2.8 ± 0.4	93 ± 2	300 ± 53	75 ± 3	12
	HEK293T†	ST171	0.90 ± 0.2	100 ± 3	- - -	- - -	7
		serotonin	0.40 ± 0.1	100	170 ± 33	50 ± 5	6
		befiradol	1.7 ± 0.6	111 ± 6	180 ± 57	71 ± 6	5
F112$^{3.28}$W	HEK293A†	ST171	1.7 ± 0.5	77 ± 9	- - -	- - -	4
		serotonin	1.6 ± 0.4	100	160 ± 73	91 ± 4	4
		befiradol	25 ± 8	96 ± 4	2700 ± 1200	90 ± 4	4
F112$^{3.28}$W + M92$^{2.60}$F	HEK293A†	ST171	1.7 ± 0.4	77 ± 5	- - -	- - -	4
		serotonin	1.7 ± 0.7	100	120 ± 8	94 ± 1	4
		befiradol	104 ± 27	92 ± 7	- - -	- - -	4

*,† Functional activity was determined in different cells lines transiently transfected with 5-HT$_{1A}$ and the * CAMYEL or † GloSensor biosensor for cAMP. ‡ Mean EC_{50} value representing the potency for the inhibition of cAMP accumulation. § Mean of the maximum activity for the inhibition of cAMP accumulation relative to the maximum effect of serotonin [=100%]. ‖ Mean EC_{50} expressing the accumulation of cAMP. ¶ Mean efficacy at saturating ligand concentrations relative to the maximum effect of inhibition of cAMP accumulation by serotonin. # Number of individual experiments.

Table S3. Statistics for 5-HT_{1A}R-mediated modulation of cAMP concentrations in Gα_s and G$\alpha_{i/o}$ deficient HEK cells, and β-arrestin 2 recruitment.

5-HT_{1A}R	ligand	inhibition of cAMP accumulation * HEK293AΔG_s		cAMP accumulation HEK293AΔ$G_{i/o}$ *		β-arrestin 2 recruitment HEK293T †	
		EC_{50} [nM ± SEM]	E_{max} ‡ [% ± SEM]	EC_{50} [nM ± SEM]	E_{max} ‡ [% ± SEM]	EC_{50} [nM ± SEM]	E_{max} ‡ [% ± SEM]
wildtype	serotonin	1.9 ± 0.2	100 (21§)	260 ± 23	100 (*16*)	310 ± 16	100 (*14*)
	befiradol	3.1 ± 0.5	87 ± 3 (*12*)	330 ± 52	74 ± 3 (*10*)	150 ± 5	79 ± 1 (*13*)
	ST171	5.3 ± 0.8	74 ± 3 (12)	- - -‖	< 5 (*5*)	- - -‖	< 5 (*11*)
	5-CT	0.087 ± 0.030	86 ± 8 (*5*)	14 ± 2	102 ± 4 (*5*)	14 ± 1	98 ± 2 (*6*)
	8-OH-DPAT	2.5 ± 0.6	79 ± 4 (*5*)	210 ± 49	31 ± 3 (*6*)	160 ± 8	53 ± 3 (*6*)
	LSD-d3	4.4 ± 0.4	87 ± 8 (*5*)	900 ± 100	48 ± 5 (*5)*	450 ± 170	70 ± 7 (*6*)
	(*S*)-(-)lisuride	40 ± 11	68 ± 4 (*5*)	1800 ± 480	29 ± 4 (*5*)	930 ± 90	46 ± 3 (*5*)
	aripiprazole	460 ± 55	90 ± 9 (*5*)	- - -‖	< 5 (*5*)	- - -‖	24 ± 3 (*3*) @ 100 μM
	buspirone	16 ± 4	58 ± 1 (*5*)	- - -‖	< 5 (*6*)	- - -‖	< 5 (*5*)
	ziprasidone	370 ± 140	57 ± 7 (*5*)	- - -‖	< 5 (*6*)	- - -‖	< 5 (*3*)
F112$^{3.28}$W	serotonin	1.5 ± 0.4	100 (*3*)	310 ± 120	100 (4)	140 ± 9	100 (*4*)
	befiradol	34 ± 13	95 ± 5 (*3*)	- - -‖	85 ± 12 (4) @ 30 μM	460 ± 60	86 ± 3 (*4*)
	ST171	2.2 ± 0.3	69 ± 8 (*3*)	- - -‖	< 5 (*4*)	- - -‖	< 5 (*4*)
F112$^{3.28}$W + M92$^{2.60}$F	serotonin	1.1 ± 0.2	100 (*3*)	91 ± 28	100 (*4*)	130 ± 14	100 (*5*)
	befiradol	100 ± 20	99 ± 7 (*3*)	- - -‖	67 ± 12 (*4*) @30 μM	1200 ± 160	83 ± 4 (*5*)
	ST171	1.8 ± 0.6	85 ± 11 (*3*)	- - -‖	< 5 (*4*)	- - -‖	< 5 (*5*)
Y96$^{2.64}$A	serotonin	1.8 ± 0.6	100 (*3*)	340 ± 93	100 (*3*)	n.d.¶	n.d.¶
	befiradol	3.9 ± 0.5	104 ± 5 (*3*)	960 ± 130	69 ± 5 (*3*)	n.d.¶	n.d.¶
	ST171	14 ± 3.5	76 ± 2 (*3*)	- - -‖	< 5 (*3*)	n.d.¶	n.d.¶
Q97$^{2.65}$A	serotonin	0.97 ± 0.35	100 (*3*)	250 ± 39	100 (*3*)	n.d.¶	n.d.¶
	befiradol	2.1 ± 0.8	91 ± 7 (*3*)	280 ± 54	60 ± 2 (*3*)	n.d.¶	n.d.¶
	ST171	8.0 ± 0.9	79 ± 6 (*3*)	- - -‖	< 5 (*3*)	n.d.¶	n.d.¶

* Functional activity was determined in different knockout cell lines transiently transfected with 5-HT_{1A}R wildtype or indicated receptor mutants and the GloSensor biosensor for cAMP. † Recruitment of β-arrestin 2-RlucII to 5-HT_{1A}R wt or mutants was determined in HEK293T cells co-transfected with GRK2 and CAAX-rGFP. ‡ Mean of efficacy relative to the maximum effect of serotonin [=100%]. § Number of individual experiments. ‖ No (complete) sigmoid concentration-response curve was observed, preventing reliable calculation of E_{max} and EC_{50}. ¶ n.d. not determined.

Table S4. Receptor expression level and type of coexpressed of G$\alpha_{i/o}$ subtype affect the relative efficacy of ST171 but not serotonin or befiradol.

Test system	ST171		serotonin		befiradol	
	EC_{50} [nM ± SEM]	E_{max} [% ± SEM]	EC_{50} [nM ± SEM]	E_{max} [%]	EC_{50} [nM ± SEM]	E_{max} [% ± SEM]
HEK293AΔ$G_{i/o}$*						
+ Gα_{i1}	2.5 ± 0.7 (*5*)	68 ± 3	1.8 ± 0.4 (*5*)	100	3.9 ± 0.5 (*5*)	100 ± 5
+ Gα_{i2}	4.4 ± 1.0 (*5*)	57 ± 7	2.8 ± 0.2 (*5*)	100	9.2 ± 1.2 (*5*)	97 ± 4
+ Gα_{i3}	2.5 ± 0.6 (*5*)	80 ± 5	1.3 ± 0.3 (*5*)	100	3.9 ± 0.7 (*5*)	105 ± 6
+ Gα_{oA}	4.1 ± 1.0(*5*)	100 ± 3	1.2 ± 0.3 (*5*)	100	3.2 ± 0.2 (*5*)	107 ± 3
+ Gα_{z}	7.0 ± 1.3 (*5*)	93 ± 4	2.6 ± 0.3 (*5*)	100	8.4 ± 2.0 (*5*)	95 ± 2
HEK293T† (5-HT_{1A}R · 10^6/cell) ‡						
+ 0.4 µg (1.5 ± 0.16)	1.9 ± 0.31 (*17*)	77 ± 3	8.7 ± 1.0 (*16*)	100	4.5 ± 0.77 (12)	102 ± 3
+ 0.2 µg (1.3 ± 0.15)	1.6 ± 0.17 (8)	66 ± 4	7.5 ± 1.5 (8)	100	7.1 ± 0.90 (*5*)	102 ± 2
+ 0.1 µg (0.70 ± 0.094)	1.6 ± 0.27 (15)	62 ± 2	19 ± 2.8 (*14*)	100	6.9 ± 1.3 (*10*)	101 ± 4
+ 0.04 µg (0.40 ± 0.070)	1.2 ± 0.23 (12)	45 ± 2	27 ± 5.6 (*12*)	100	11 ± 2.2 (*10*)	103 ± 3
+ 0.01 µg (0.081 ± 0.009)	19 ± 10 (*8*)	37 ± 5	24 ± 6.3 (*6*)	100	11 ± 6.7 (*4*)	99 ± 9

* Functional activity was determined in HEK293AΔ$G_{i/o}$ cells transiently co-transfected with 5-HT_{1A}R, one Gα_i subunits (Gα_{i1}, Gα_{i2}, Gα_{i3}, Gα_{oA}, or Gα_z) and the cAMP GloSensor in (*n*) independent experiments, each performed in triplicates, EC_{50} indicates potency for the inhibition of cAMP accumulation, E_{max} indicates efficacy of the inhibition of cAMP accumulation, relative to the maximum inhibitory effect of serotonin [=100%]. † Functional activity was determined with a Gα_{i1}RLuc2/Gγ_2-GFP10 BRET biosensor, with EC_{50} indicating the potency and E_{max} the efficacy relative to the maximum effect of serotonin [=100%] from (*n*) independent experiments. ‡ Amount of transfected cDNA per culture dish and resulting 5-HT_{1A}R density expressed determined by whole cell saturation binding experiments with [^{3}H]WAY600,135.

Table S5. Binding affinity for ST171 for a set of class A GPCRs reveals its selectivity for 5-HT_{1A}R.

GPCR family*	subtype	ST171 affinity			experimental conditions		
		K_i [nM ± SD]	pK_i	n†	radioligand	K_D‡	B_{max}§
serotonin	5-HT_{1A}	0.41 ± 0.19	9.433	11	[^{3}H]WAY100635	0.080	3700
	5-HT_{2A}	280 ± 95	6.585	10	[^{3}H]ketanserin	0.29	1400
	5-HT_6	3100 ± 5800	5.511	4	[^{3}H]N-methyl-LSD	2.3	800
dopamine	D_1	260 ± 170	6.680	4	[^{3}H]SCH23390	0.29	4000
	D_{2long}‖	21 ± 13	7.637	6	[^{3}H]spiperone	0.078	1300
	D_{2short}‖	8.5 ± 5.0	8.007	6	[^{3}H]spiperone	0.080	6800
	D_3‖	22 ± 11	7.583	7	[^{3}H]spiperone	0.16	6300
	D_4‖	0.17 ± 0.09	9.742	6	[^{3}H]spiperone	0.20	1200
	D_5	740 ± 410	6.188	4	[^{3}H]SCH23390	0.26	1400
adrenergic	α_{1A}	0.49 ± 0.24	9.361	9	[^{3}H]prazosine	0.35	3900
	α_{1B}	6.7 ± 2.0	8.191	4	[^{3}H]prazosine	0.11	3600
	α_{2A}	260 ± 210	6.690	4	[^{3}H]RX821002	0.48	1500
	α_{2B}	110 ± 60	6.989	4	[^{3}H]RX821002	1.75	3900
	α_{2C}	28 ± 12	7.587	4	[^{3}H]RX821002	0.60	7000
	β_1	1700 ± 350	5.788	2	[^{3}H]CGP12177	0.080	3500
	β_2	100 ± 11	6.992	4	[^{3}H]CGP12177	0.095	1500
muscarinic	M_1	10000 ± 920	4.986	2	[^{3}H]NMS	0.050	5600
	M_2	18000 ± 9200	4.789	2	[^{3}H]NMS	0.18	1500
	M_3	49000 ± 28000	4.382	3	[^{3}H]NMS	0.18	3500
opioid	μOR	8600 ± 2100	5.074	2	[^{3}H]diprenorphine	0.080	1800
	δOR	24000 ± 3500	4.631	2	[^{3}H]diprenorphine	0.19	2500
	κOR	6200 ± 850	5.202	2	[^{3}H]diprenorphine	0.070	4500
orexin	OX_1	>50000	<4.300	2	[^{3}H]SB674042	0.62	8300
	OX_2	>50000	<4.300	2	[^{3}H]EMPA	1.1	7000
neurotensin	NTS_1	>50000	<4.300	2	[^{3}H]NT(8-13)¶	0.70	10000
	NTS_2	>50000	<4.300	2	[^{3}H]NT(8-13)¶	1.8	700

* Radioligand displacement experiments with membranes from HEK 293T cells transiently transfected with the appropriate receptor. † Number of individual experiments each done in triplicate. ‡ Binding affinity for the used radioligand as K_D value in nM. § Receptor density as B_{max} in fmol/mg protein. ‖ Dopamine D_{2long}, D2$_{short}$, D_3 and $D_{4.4}$ receptors were stably expressed in CHO cells. ¶ [^{3}H]NT8-13 was custom synthesized by Novandi, Södertälje, Sweden.

REFERENCES AND NOTES

1. R. Benyamin, A. M. Trescot, S. Datta, R. Buenaventura, R. Adlaka, N. Sehgal, S. E. Glaser, R. Vallejo, Opioid complications and side effects. *Pain Physician* **11**, S105–S120 (2008).

2. S. L. Walsh, K. L. Preston, G. E. Bigelow, M. L. Stitzer, Acute administration of buprenorphine in humans: Partial agonist and blockade effects. *J. Pharmacol. Exp. Ther.* **274**, 361–372 (1995).

3. A. Manglik, H. Lin, D. K. Aryal, J. D. McCorvy, D. Dengler, G. Corder, A. Levit, R. C. Kling, V. Bernat, H. Hübner, X.-P. Huang, M. F. Sassano, P. M. Giguère, S. Löber, D. Da, G. Scherrer, B. K. Kobilka, P. Gmeiner, B. L. Roth, B. K. Shoichet, Structure-based discovery of opioid analgesics with reduced side effects. *Nature* **537**, 185–190 (2016).

4. H. Wang, F. Hetzer, W. Huang, Q. Qu, J. Meyerowitz, J. Kaindl, H. Hübner, G. Skiniotis, B. K. Kobilka, P. Gmeiner, Structure-based evolution of G protein-biased μ-opioid receptor agonists. *Angew. Chem. Int. Ed. Engl.* **61**, e202200269 (2022).

5. D. E. Nichols, C. D. Nichols, Serotonin receptors. *Chem. Rev.* **108**, 1614–1641 (2008).

6. P. Celada, A. Bortolozzi, F. Artigas, Serotonin 5-HT_{1A} receptors as targets for agents to treat psychiatric disorders: Rationale and current status of research. *CNS Drugs* **27**, 703–716 (2013).

7. A. L. Warren, D. Lankri, M. J. Cunningham, I. C. Serrano, L. F. Parise, A. C. Kruegel, P. Duggan, G. Zilberg, M. J. Capper, V. Havel, S. J. Russo, D. Sames, D. Wacker, Structural pharmacology and therapeutic potential of 5-methoxytryptamines. *Nature* **630**, 237–246 (2024).

8. P. Xu, S. Huang, H. Zhang, C. Mao, X. E. Zhou, X. Cheng, I. A. Simon, D.-D. Shen, H.-Y. Yen, C. V. Robinson, K. Harpsøe, B. Svensson, J. Guo, H. Jiang, D. E. Gloriam, K. Melcher, Y. Jiang, Y. Zhang, H. E. Xu, Structural insights into the lipid and ligand regulation of serotonin receptors. *Nature* **592**, 469–473 (2021).

9. Z. Chen, J. Yu, H. Wang, P. Xu, L. Fan, F. Sun, S. Huang, P. Zhang, H. Huang, S. Gu, B. Zhang, Y. Zhou, X. Wan, G. Pei, H. E. Xu, J. Cheng, S. Wang, Flexible scaffold-based cheminformatics approach for polypharmacological drug design. *Cell* **187**, 2194–2208.e22 (2024).

10. J. Gjerstad, A. Tjølsen, K. Hole, The effect of 5-HT_{1A} receptor stimulation on nociceptive dorsal horn neurones in rats. *Eur. J. Pharmacol.* **318**, 315–321 (1996).

11. R. Bardoni, Serotonergic modulation of nociceptive circuits in spinal cord dorsal horn. *Curr. Neuropharmacol.* **17**, 1133–1145 (2019).

12. M. J. Millan, Descending control of pain. *Prog. Neurobiol.* **66**, 355–474 (2002).

13. Q. Q. Liu, X. X. Yao, S. H. Gao, R. Li, B. J. Li, W. Yang, R. J. Cui, Role of 5-HT receptors in neuropathic pain: Potential therapeutic implications. *Pharmacol. Res.* **159**, 104949 (2020).

14. A. Dray, Inflammatory mediators of pain. *Br. J. Anaesth.* **75**, 125–131 (1995).

15. K. Okamoto, H. Imbe, Y. Morikawa, M. Itoh, M. Sekimoto, K. Nemoto, E. Senba, 5-HT_{2A} receptor subtype in the peripheral branch of sensory fibers is involved in the potentiation of inflammatory pain in rats. *Pain* **99**, 133–143 (2002).

16. N. M. E. Carmichael, M. P. Charlton, J. O. Dostrovsky, Activation of the 5-$HT_{1B/D}$ receptor reduces hindlimb neurogenic inflammation caused by sensory nerve stimulation and capsaicin. *Pain* **134**, 97–105 (2008).

17. K. P. Zeitz, N. Guy, A. B. Malmberg, S. Dirajlal, W. J. Martin, L. Sun, D. W. Bonhaus, C. L. Stucky, D. Julius, A. I. Basbaum, The 5-HT_3 subtype of serotonin receptor contributes to nociceptive processing via a novel subset of myelinated and unmyelinated nociceptors. *J. Neurosci.* **22**, 1010 (2002).

18. M. Sasaki, H. Obata, K. Kawahara, S. Saito, F. Goto, Peripheral 5-HT_{2A} receptor antagonism attenuates primary thermal hyperalgesia and secondary mechanical allodynia after thermal injury in rats. *Pain* **122**, 130–136 (2006).

19. V. Kayser, B. Aubel, M. Hamon, S. Bourgoin, The antimigraine 5-$HT_{1B/1D}$ receptor agonists, sumatriptan, zolmitriptan and dihydroergotamine, attenuate pain-related behaviour in a rat model of trigeminal neuropathic pain. *Br. J. Pharmacol.* **137**, 1287–1297 (2002).

20. T. Nikai, A. I. Basbaum, A. H. Ahn, Profound reduction of somatic and visceral pain in mice by intrathecal administration of the anti-migraine drug, sumatriptan. *Pain* **139**, 533–540 (2008).

21. A. Dogrul, M. H. Ossipov, F. Porreca, Differential mediation of descending pain facilitation and inhibition by spinal 5HT-3 and 5HT-7 receptors. *Brain Res.* **1280**, 52–59 (2009).

22. M. J. Millan, L. Seguin, P. Honoré, S. Girardon, K. Bervoets, Pro- and antinociceptive actions of serotonin (5-HT)1A agonists and antagonists in rodents: Relationship to algesiometric paradigm. *Behav. Brain Res.* **73**, 69–77 (1995).

23. L. Bardin, F. C. Colpaert, Role of spinal 5-HT_{1A} receptors in morphine analgesia and tolerance in rats. *Eur. J. Pain* **8**, 253–261 (2004).

24. H.-J. Jeong, V. A. Mitchell, C. W. Vaughan, Role of 5-HT_1 receptor subtypes in the modulation of pain and synaptic transmission in rat spinal superficial dorsal horn. *Br. J. Pharmacol.* **165**, 1956–1965 (2012).

25. K. Sałat, M. Kołaczkowski, A. Furgała, A. Rojek, J. Śniecikowska, M. A. Varney, A. Newman-Tancredi, Antinociceptive, antiallodynic and antihyperalgesic effects of the 5-HT_{1A} receptor selective agonist, NLX-112 in mouse models of pain. *Neuropharmacology* **125**, 181–188 (2017).

26. L. Bardin, M. Bardin, J. Lavarenne, A. Eschalier, Effect of intrathecal serotonin on nociception in rats: Influence of the pain test used. *Exp. Brain Res.* **113**, 81–87 (1997).

27. R. Nadeson, C. S. Goodchild, Antinociceptive role of 5-HT_{1A} receptors in rat spinal cord. *Br. J. Anaesth.* **88**, 679–684 (2002).

28. C. Schmauss, D. L. Hammond, J. W. Ochi, T. L. Yaksh, Pharmacological antagonism of the antinociceptive effects of serotonin in the rat spinal cord. *Eur. J. Pharmacol.* **90**, 349–357 (1983).

29. N. Mjellem, A. Lund, P. K. Eide, R. Størkson, A. Tjølsen, The role of 5-HT_{1A} and 5-HT_{1B} receptors in spinal nociceptive transmission and in the modulation of NMDA induced behaviour. *Neuroreport* **3**, 1061–1064 (1992).

30. X. Khawaja, Quantitative autoradiographic characterisation of the binding of [^{3}H]WAY-100635, a selective 5-HT$_{1A}$ receptor antagonist. *Brain Res.* **673**, 217–225 (1995).

31. H. K. Kia, M.-C. Miquel, M.-J. Brisorgueil, G. Daval, M. Riad, S. E. Mestikawy, M. Hamon, D. Vergé, Immunocytochemical localization of serotonin$_{1A}$ receptors in the rat central nervous system. *J. Comp. Neurol.* **365**, 289–305 (1996).

32. G. Daval, D. Vergé, A. I. Basbaum, S. Bourgoin, M. Hamon, Autoradiographic evidence of serotonin1 binding sites on primary afferent fibres in the dorsal horn of the rat spinal cord. *Neurosci. Lett.* **83**, 71–76 (1987).

33. A. Levit Kaplan, R. T. Strachan, J. M. Braz, V. Craik, S. Slocum, T. Mangano, V. Amabo, H. O'Donnell, P. Lak, A. I. Basbaum, B. L. Roth, B. K. Shoichet, Structure-based design of a chemical probe set for the 5-HT$_{5A}$ serotonin receptor. *J. Med. Chem.* **65**, 4201–4217 (2022).

34. X.-J. Xu, F. Colpaert, Z. Wiesenfeld-Hallin, Opioid hyperalgesia and tolerance versus 5-HT$_{1A}$ receptor-mediated inverse tolerance. *Trends Pharmacol. Sci.* **24**, 634–639 (2003).

35. D. J. Haleem, Serotonin-1A receptor dependent modulation of pain and reward for improving therapy of chronic pain. *Pharmacol. Res.* **134**, 212–219 (2018).

36. J. Ren, X. Ding, J. J. Greer, 5-HT$_{1A}$ receptor agonist befiradol reduces fentanyl-induced respiratory depression, analgesia, and sedation in rats. *Anesthesiology* **122**, 424–434 (2015).

37. A. Newman-Tancredi, L. Bardin, A. Auclair, F. Colpaert, R. Depoortère, M. A. Varney, NLX-112, a highly selective 5-HT$_{1A}$ receptor agonist, mediates analgesia and antidepressant-like activity in rats via spinal cord and prefrontal cortex 5-HT$_{1A}$ receptors, respectively. *Brain Res.* **1688**, 1–7 (2018).

38. K. Deseure, S. Bréand, F. C. Colpaert, Curative-like analgesia in a neuropathic pain model: Parametric analysis of the dose and the duration of treatment with a high-efficacy 5-HT$_{1A}$ receptor agonist. *Eur. J. Pharmacol.* **568**, 134–141 (2007).

39. R. Fisher, A. Hikima, R. Morris, M. J. Jackson, S. Rose, M. A. Varney, R. Depoortere, A. Newman-Tancredi, The selective 5-HT_{1A} receptor agonist, NLX-112, exerts anti-dyskinetic and anti-parkinsonian-like effects in MPTP-treated marmosets. *Neuropharmacology* **167**, 107997 (2020).

40. J. Lyu, S. Wang, T. E. Balius, I. Singh, A. Levit, Y. S. Moroz, M. J. O'Meara, T. Che, E. Algaa, K. Tolmachova, A. A. Tolmachev, B. K. Shoichet, B. L. Roth, J. J. Irwin, Ultra-large library docking for discovering new chemotypes. *Nature* **566**, 224–229 (2019).

41. E. A. Fink, J. Xu, H. Hübner, J. M. Braz, P. Seemann, C. Avet, V. Craik, D. Weikert, M. F. Schmidt, C. M. Webb, N. A. Tolmachova, Y. S. Moroz, X.-P. Huang, C. Kalyanaraman, S. Gahbauer, G. Chen, Z. Liu, M. P. Jacobson, J. J. Irwin, M. Bouvier, Y. Du, B. K. Shoichet, A. I. Basbaum, P. Gmeiner, Structure-based discovery of nonopioid analgesics acting through the α_{2A}-adrenergic receptor. *Science* **377**, eabn7065 (2022).

42. A. Newman-Tancredi, R. Y. Depoortère, M. S. Kleven, M. Kołaczkowski, L. Zimmer, Translating biased agonists from molecules to medications: Serotonin 5-HT_{1A} receptor functional selectivity for CNS disorders. *Pharmacol. Ther.* **229**, 107937 (2022).

43. K. Sałaciak, K. Pytka, Biased agonism in drug discovery: Is there a future for biased 5-HT_{1A} receptor agonists in the treatment of neuropsychiatric diseases?. *Pharmacol. Ther.* **227**, 107872 (2021).

44. P. Linciano, C. Sorbi, A. Comitato, A. Lesniak, M. Bujalska-Zadrożny, A. Pawłowska, A. Bielenica, J. Orzelska-Górka, E. Kędzierska, G. Biała, S. Ronsisvalle, S. Limoncella, L. Casarini, E. Cichero, P. Fossa, G. Satała, A. J. Bojarski, L. Brasili, R. Bardoni, S. Franchini, Identification of a potent and selective 5-HT_{1A} receptor agonist with *in vitro* and *in vivo* antinociceptive activity. *ACS Chem. Neurosci.* **11**, 4111–4127 (2020).

45. J. R. Raymond, Y. V. Mukhin, T. W. Gettys, M. N. Garnovskaya, The recombinant 5-HT_{1A} receptor: G protein coupling and signalling pathways. *Br. J. Pharmacol.* **127**, 1751–1764 (1999).

46. R. E. West, J. Moss, M. Vaughan, T. Liu, T. Y. Liu, Pertussis toxin-catalyzed ADP-ribosylation of transducin. Cysteine 347 is the ADP-ribose acceptor site. *J. Biol. Chem.* **260**, 14428–14430 (1985).

47. Y. Ono, K. Kawakami, G. Nakamura, S. Ishida, J. Aoki, A. Inoue, Generation of Gαi knock-out HEK293 cells illuminates Gαi-coupling diversity of GPCRs. *Commun. Biol.* **6**, 112 (2023).

48. W. Stallaert, E. T. van der Westhuizen, A. M. Schönegge, B. Plouffe, M. Hogue, V. Lukashova, A. Inoue, S. Ishida, J. Aoki, C. Le Gouill, M. Bouvier, Purinergic receptor transactivation by the β_2-adrenergic receptor increases intracellular Ca^{2+} in nonexcitable cells. *Mol. Pharmacol.* **91**, 533–544 (2017).

49. M. O'Hayre, K. Eichel, S. Avino, X. Zhao, D. J. Steffen, X. Feng, K. Kawakami, J. Aoki, K. Messer, R. Sunahara, A. Inoue, M. von Zastrow, J. S. Gutkind, Genetic evidence that β-arrestins are dispensable for the initiation of β_2-adrenergic receptor signaling to ERK. *Sci. Signal.* **10**, eaal3395 (2017).

50. Y. Namkung, C. Le Gouill, V. Lukashova, H. Kobayashi, M. Hogue, E. Khoury, M. Song, M. Bouvier, S. A. Laporte, Monitoring G protein-coupled receptor and β-arrestin trafficking in live cells using enhanced bystander BRET. *Nat. Commun.* **7**, 12178 (2016).

51. Y. Toyoda, A. Zhu, F. Kong, S. Shan, J. Zhao, N. Wang, X. Sun, L. Zhang, C. Yan, B. K. Kobilka, X. Liu, Structural basis of α_{1A}-adrenergic receptor activation and recognition by an extracellular nanobody. *Nat. Commun.* **14**, 3655 (2023).

52. S. D. Shields, W. A. Eckert, A. I. Basbaum, Spared nerve injury model of neuropathic pain in the mouse: A behavioral and anatomic analysis. *J. Pain* **4**, 465–470 (2003).

53. M. Papp, P. Willner, 8-OH-DPAT-induced place preference and place aversion: Effects of PCPA and dopamine antagonists. *Psychopharmacology* **103**, 99–102 (1991).

54. J. L. Neisewander, S. A. McDougall, S. L. Bowling, M. T. Bardo, Conditioned taste aversion and place preference with buspirone and gepirone. *Psychopharmacology* **100**, 485–490 (1990).

55. J. C. Cole, R. J. Rodgers, Ethological evaluation of the effects of acute and chronic buspirone treatment in the murine elevated plus-maze test: Comparison with haloperidol. *Psychopharmacology* **114**, 288–296 (1994).

56. S. M. Remy, R. Schreiber, M. Dalmus, J. De Vry, Somatodendritic 5-HT_{1A} receptors are critically involved in the anxiolytic effects of 8-OH-DPAT. *Psychopharmacology* **125**, 89–91 (1996).

57. S. Pellow, S. E. File, Anxiolytic and anxiogenic drug effects on exploratory activity in an elevated plus-maze: A novel test of anxiety in the rat. *Pharmacol. Biochem. Behav.* **24**, 525–529 (1986).

58. S. Pellow, A. L. Johnston, S. E. File, Selective agonists and antagonists for 5-hydroxytryptamine receptor subtypes, and interactions with yohimbine and FG 7142 using the elevated plus-maze test in the rat. *J. Pharm. Pharmacol.* **39**, 917–928 (1987).

59. W. H. Powell, L. E. Annett, R. Depoortere, A. Newman-Tancredi, M. M. Iravani, The selective 5-HT_{1A} receptor agonist NLX-112 displays anxiolytic-like activity in mice. *Naunyn Schmiedeberg's Arch. Pharmacol.* **395**, 149–157 (2022).

60. W. I. Weis, B. K. Kobilka, The molecular basis of G protein–coupled receptor activation. *Annu. Rev. Biochem.* **87**, 897–919 (2018).

61. W. Yin, X. E. Zhou, D. Yang, P. W. de Waal, M. Wang, A. Dai, X. Cai, C.-Y. Huang, P. Liu, X. Wang, Y. Yin, B. Liu, Y. Zhou, J. Wang, H. Liu, M. Caffrey, K. Melcher, Y. Xu, M.-W. Wang, H. E. Xu, Y. Jiang, Crystal structure of the human 5-HT_{1B} serotonin receptor bound to an inverse agonist. *Cell Discov.* **4**, 12 (2018).

62. M. Casiraghi, H. Wang, P. C. Brennan, C. Habrian, H. Hübner, M. F. Schmidt, L. Maul, B. Pani, S. M. F. M. Bahriz, B. Xu, N. Staffen, T. E. Assafa, B. Chen, E. White, R. K. Sunahara, A. Inoue, Y. K. Xiang, R. J. Lefkowitz, E. Y. Isacoff, N. Nucci, P. Gmeiner, M. T. Lerch, B. K. Kobilka, Structure and dynamics determine G protein coupling specificity at a class A GPCR. *Sci. Adv.* **11**, eadq3971 (2025).

63. A. J. Kooistra, S. Kuhne, I. J. P. de Esch, R. Leurs, C. de Graaf, A structural chemogenomics analysis of aminergic GPCRs: Lessons for histamine receptor ligand design. *Br. J. Pharmacol.* **170**, 101–126 (2013).

64. H. S. Biswal, S. Wategaonkar, Nature of the N−H···S hydrogen bond. *J. Phys. Chem. A* **113**, 12763–12773 (2009).

65. Q. Qu, W. Huang, D. Aydin, J. M. Paggi, A. B. Seven, H. Wang, S. Chakraborty, T. Che, J. F. DiBerto, M. J. Robertson, A. Inoue, C.-M. Suomivuori, B. L. Roth, S. Majumdar, R. O. Dror, B. K. Kobilka, G. Skiniotis, Insights into distinct signaling profiles of the μOR activated by diverse agonists. *Nat. Chem. Biol.* **19**, 423–430 (2023).

66. A. Brenchat, L. Romero, M. García, M. Pujol, J. Burgueño, A. Torrens, M. Hamon, J. M. Baeyens, H. Buschmann, D. Zamanillo, J. M. Vela, 5-HT_7 receptor activation inhibits mechanical hypersensitivity secondary to capsaicin sensitization in mice. *Pain* **141**, 239–247 (2009).

67. D. W. Gil, C. V. Cheevers, K. M. Kedzie, C. A. Manlapaz, S. Rao, *E. Tang*, J. E. Donello, α-1-Adrenergic receptor agonist activity of clinical α-adrenergic receptor agonists interferes with α-2-mediated analgesia. *Anesthesiology* **110**, 401–407 (2009).

68. T. A. Gee, N. C. Weintraub, D. Lu, C. E. Phelps, E. Navratilova, M. L. Heien, F. Porreca, A pain-induced tonic hypodopaminergic state augments phasic dopamine release in the nucleus accumbens. *Pain* **161**, 2376–2384 (2020).

69. H. Hübner, C. Haubmann, W. Utz, P. Gmeiner, Conjugated enynes as nonaromatic catechol bioisosteres: Synthesis, binding experiments, and computational studies of novel dopamine receptor agonists recognizing preferentially the D_3 subtype. *J. Med. Chem.* **43**, 756–762 (2000).

70. L. I. Jiang, J. Collins, R. Davis, K.-M. Lin, D. DeCamp, T. Roach, R. Hsueh, R. A. Rebres, E. M. Ross, R. Taussig, I. Fraser, P. C. Sternweis, Use of a cAMP BRET sensor to characterize a novel regulation of cAMP by the sphingosine 1-phosphate/G_{13} pathway. *J. Biol. Chem.* **282**, 10576–10584 (2007).

71. J. Köckenberger, O. Fischer, A. Konopa, S. Bergwinkl, S. Mühlich, P. Gmeiner, R. J. Kutta, H. Hübner, M. Keller, M. R. Heinrich, Synthesis, characterization, and application of muscarinergic M_3 receptor ligands linked to fluorescent dyes. *J. Med. Chem.* **65**, 16494–16509 (2022).

72. C. Galés, J. J. J. Van Durm, S. Schaak, S. Pontier, Y. Percherancier, M. Audet, H. Paris, M. Bouvier, Probing the activation-promoted structural rearrangements in preassembled receptor–G protein complexes. *Nat. Struct. Mol. Biol.* **13**, 778–786 (2006).

73. D. Möller, A. Banerjee, T. C. Uzuneser, M. Skultety, T. Huth, B. Plouffe, H. Hübner, C. Alzheimer, K. Friedland, C. P. Müller, M. Bouvier, P. Gmeiner, Discovery of G protein-biased dopaminergics with a pyrazolo[1,5-*a*]pyridine substructure. *J. Med. Chem.* **60**, 2908–2929 (2017).

74. S. R. Chaplan, F. W. Bach, J. W. Pogrel, J. M. Chung, T. L. Yaksh, Quantitative assessment of tactile allodynia in the rat paw. *J. Neurosci. Methods* **53**, 55–63 (1994).

75. I. Singh, A. Seth, C. B. Billesbølle, J. Braz, R. M. Rodriguiz, K. Roy, B. Bekele, V. Craik, X.-P. Huang, D. Boytsov, V. M. Pogorelov, *P. Lak*, H. O'Donnell, W. Sandtner, J. J. Irwin, B. L. Roth, A. I. Basbaum, W. C. Wetsel, A. Manglik, B. K. Shoichet, G. Rudnick, Structure-based discovery of conformationally selective inhibitors of the serotonin transporter. *Cell* **186**, 2160–2175.e17 (2023).

76. D. L. Juarez-Salinas, J. M. Braz, K. A. Hamel, A. I. Basbaum, Pain relief by supraspinal gabapentin requires descending noradrenergic inhibitory controls. *Pain Rep.* **3**, e659 (2018).

77. Y.-L. Liang, P. Zhao, C. Draper-Joyce, J.-A. Baltos, A. Glukhova, T. T. Truong, L. T. May, A. Christopoulos, D. Wootten, P. M. Sexton, S. G. B. Furness, Dominant negative G proteins enhance formation and purification of agonist-GPCR-G protein complexes for structure determination. *ACS Pharmacol. Transl. Sci.* **1**, 12–20 (2018).

78. S. Q. Zheng, E. Palovcak, J.-P. Armache, K. A. Verba, Y. Cheng, D. A. Agard, MotionCor2: Anisotropic correction of beam-induced motion for improved cryo-electron microscopy. *Nat. Methods* **14**, 331–332 (2017).

79. A. Punjani, J. L. Rubinstein, D. J. Fleet, M. A. Brubaker, cryoSPARC: Algorithms for rapid unsupervised cryo-EM structure determination. *Nat. Methods* **14**, 290–296 (2017).

80. D. Kimanius, L. Dong, G. Sharov, T. Nakane, S. H. W. Scheres, New tools for automated cryo-EM single-particle analysis in RELION-4.0. *Biochem. J.* **478**, 4169–4185 (2021).

81. D. Liebschner, P. V. Afonine, M. L. Baker, G. Bunkoczi, V. B. Chen, T. I. Croll, B. Hintze, L.-W. Hung, S. Jain, A. J. McCoy, N. W. Moriarty, R. D. Oeffner, B. K. Poon, M. G. Prisant, R. J. Read, J. S. Richardson, D. C. Richardson, M. D. Sammito, O. V. Sobolev, D. H. Stockwell, T. C. Terwilliger, A. G. Urzhumtsev, L. L. Videau, C. J. Williams, P. D. Adams, Macromolecular structure determination using X-rays, neutrons and electrons: Recent developments in Phenix. *Acta Crystallogr. Sect. D Struct. Biol.* **75**, 861–877 (2019).

82. E. F. Pettersen, T. D. Goddard, C. C. Huang, G. S. Couch, D. M. Greenblatt, E. C. Meng, T. E. Ferrin, UCSF Chimera—A visualization system for exploratory research and analysis. *J. Comput. Chem.* **25**, 1605–1612 (2004).

83. P. Emsley, B. Lohkamp, W. G. Scott, K. Cowtan, Features and development of Coot. *Acta Crystallogr. Sect. D Biol. Crystallogr.* **66**, 486–501 (2010).

84. P. D. Adams, P. V. Afonine, G. Bunkóczi, V. B. Chen, I. W. Davis, N. Echols, J. J. Headd, L.-W. Hung, G. J. Kapral, R. W. Grosse-Kunstleve, A. J. McCoy, N. W. Moriarty, R. Oeffner, R. J. Read, D. C. Richardson, J. S. Richardson, T. C. Terwilliger, P. H. Zwart, PHENIX: A comprehensive Python-based system for macromolecular structure solution. *Acta Crystallogr. Sect. D Biol. Crystallogr.* **66**, 213–221 (2010).

85. A. Šali, T. L. Blundell, Comparative protein modelling by satisfaction of spatial restraints. *J. Mol. Biol.* **234**, 779–815 (1993).

86. D. A. Case, H. M. Aktulga, K. Belfon, I. Y. Ben-Shalom, J. T. Berryman, S. R. Brozell, D. S. Cerutti, I. T.E. Cheatham, G. A. Cisneros, V. W. D. Cruzeiro, T. A. Darden, R. E. Duke, G. Giambasu, M. K. Gilson, H. Gohlke, A. W. Goetz, R. Harris, S. Izadi, S. A. Izmailov, K. Kasavajhala, M. C. Kaymak, E. King, A. Kovalenko, T. Kurtzman, T. S. Lee, S. LeGrand, P. Li,

C. Lin, J. Liu, T. Luchko, R. Luo, M. Machado, V. Man, M. Manathunga, K. M. Merz, Y. Miao, O. Mikhailovskii, G. Monard, H. Nguyen, K. A. O'Hearn, A. Onufriev, F. Pan, S. Pantano, R. Qi, A. Rahnamoun, D. R. Roe, A. Roitberg, C. Sagui, S. Schott-Verdugo, A. Shajan, J. Shen, C. L. Simmerling, N. R. Skrynnikov, J. Smith, J. Swails, R. C. Walker, J. Wang, J. Wang, H. Wei, R. M. Wolf, X. Wu, Y. Xiong, Y. Xue, D. M. York, S. Zhao, P. A. Kollman, "Amber 2022" (University of California, San Francisco, 2022).

87. M. G. Wolf, M. Hoefling, C. Aponte-Santamaría, H. Grubmüller, G. Groenhof, g_membed: Efficient insertion of a membrane protein into an equilibrated lipid bilayer with minimal perturbation. *J. Comput. Chem.* **31**, 2169–2174 (2010).

88. D. Van Der Spoel, E. Lindahl, B. Hess, G. Groenhof, A. E. Mark, H. J. C. Berendsen, GROMACS: Fast, flexible, and free. *J. Comput. Chem.* **26**, 1701–1718 (2005).

89. The PLUMED consortium, Promoting transparency and reproducibility in enhanced molecular simulations. *Nat. Methods* **16**, 670–673 (2019).

90. C. Materne, I. Khan, "Method for preparing 2-alkoxyphenoxyethanamines from 2-alkoxyphenoxyethylacetamides," WO 03095416(A1)2003 (2003).

91. W. Cawello, M. Braun, H. Boekens, Absorption, disposition, metabolic fate, and elimination of the dopamine agonist rotigotine in man: Administration by intravenous infusion or transdermal delivery. *Drug Metab. Dispos.* **37**, 2055–2060 (2009).

92. G. H. B. Nardotto, E. B. Coelho, M. P. Marques, V. L. Lanchote, Chiral analysis of carvedilol and its metabolites hydroxyphenyl carvedilol and O-desmethyl carvedilol in human plasma by liquid chromatography-tandem mass spectrometry: Application to a clinical pharmacokinetic study. *J. Chromatogr. B Analyt. Technol. Biomed. Life Sci.* **1015–1016**, 173–180 (2016).

93. J. Watson, S. Wright, A. Lucas, K. L. Clarke, J. Viggers, S. Cheetham, P. Jeffrey, R. Porter, K. D. Read, Receptor occupancy and brain free fraction. *Drug Metab. Dispos.* **37**, 753–760 (2009).

94. S. G. F. Rasmussen, B. T. DeVree, Y. Zou, A. C. Kruse, K. Y. Chung, T. S. Kobilka, F. S. Thian, P. S. Chae, E. Pardon, D. Calinski, J. M. Mathiesen, S. T. A. Shah, J. A. Lyons, M.

Caffrey, S. H. Gellman, J. Steyaert, G. Skiniotis, W. I. Weis, R. K. Sunahara, B. K. Kobilka, Crystal structure of the β2 adrenergic receptor–Gs protein complex. *Nature* **477**, 549–555 (2011).

95. G. P. Moss, Basic terminology of stereochemistry (IUPAC Recommendations 1996). *Pure Appl. Chem.* **68**, 2193–2222 (1996).
